# Specific TBC Domain-Containing Proteins Control the ER-Golgi-Plasma Membrane Trafficking of GPCRs

**DOI:** 10.1016/j.celrep.2019.05.033

**Published:** 2019-07-09

**Authors:** Zhe Wei, Maoxiang Zhang, Chunman Li, Wei Huang, Yi Fan, Jianhui Guo, Mostafa Khater, Mitsunori Fukuda, Zheng Dong, Gang Hu, Guangyu Wu

**Affiliations:** 1 Department of Pharmacology and Toxicology, Medical College of Georgia, Augusta University, Augusta, GA 30912, USA; 2 Department of Integrative Life Sciences, Graduate School of Life Sciences, Tohoku University, Sendai, Miyagi, Japan; 3 Department of Cell Biology and Anatomy, Medical College of Georgia, Augusta University, Augusta, GA 30912, USA; 4 Charlie Norwood VA Medical Center, Augusta, GA 30904, USA; 5 Department of Pharmacology, Nanjing University of Chinese Medicine, Nanjing, China; 6 These authors contributed equally; 7 Lead Contact

## Abstract

G-protein-coupled receptors (GPCRs) constitute the largest superfamily of cell surface signaling proteins. However, the molecular mechanisms underlying their cell surface delivery after synthesis remain poorly understood. Here, we screen the TBC domain-containing proteins, putative Rab GTPase-activating proteins (GAPs), in the intracellular trafficking of GPCRs and identify several TBC proteins that activity-dependently regulate the anterograde transport, *en route* from the endoplasmic reticulum to the Golgi or from the Golgi to the cell surface, of several prototypic GPCR members without affecting other plasma membrane proteins. We also show that TBC1D6 functions as a GAP for Rab26, physically associates with Rab26, and attenuates Rab26 interaction with GPCRs. Furthermore, both overexpression and depletion of TBC1D6 inhibit the post-Golgi traffic of GPCRs. These data demonstrate important roles of the TBC proteins in forward trafficking of nascent GPCRs and reveal regulatory mechanisms of GPCR targeting to the functional destination.

## In Brief

Wei et al. report that several TBC proteins specifically and activity-dependently regulate ER-Golgi-plasma membrane transport of nascent GPCRs. They also show that TBC1D6 is a GAP for Rab26 and controls GPCR post-Golgi traffic. Their results reveal crucial roles of TBC proteins in and provide regulatory mechanisms of GPCR trafficking.

## Graphical Abstract

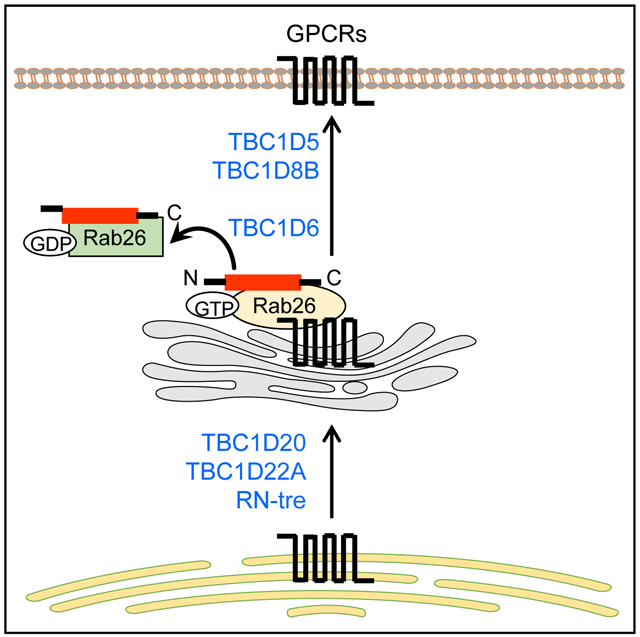

## INTRODUCTION

Rab GTPases are the master regulators of vesicle-mediated membrane traffic and regulate almost every trafficking step involved in endocytosis and exocytosis (Pfeffer, 2017; Stenmark, 2009). More than 60 Rab GTPases have been identified in mammals, forming the largest branch of the Ras-related GTPase. As with all GTPases, the function of Rab GTPases is crucially regulated by their cycle between an inactive GDP-bound state and an active GTP-bound state that are under control by two proteins: a guanine nucleotide exchange factor (GEF) that accelerates the release of GDP from Rabs and a GTPase-activating protein (GAP) that enhances the intrinsically low GTP hydrolysis rate of GTP-bound Rab. In the GDP-bound state, Rab GTPases are extracted from membranes by GDP dissociation inhibitors that regulate both Rab expression in the cytoplasm and recruitment onto the correct subcellular location. In the GTP-bound state, Rab GTPases can bind to diverse downstream effectors to ensure proper vesicle sorting, motility, tethering, and fusion with the appropriate membranes (Grosshans et al., 2006).

Recent studies have shown that Tre2-Bub2-Cdc16 (TBC) domain-containing proteins are specific GAPs for Rab GTPases (Barr and Lambright, 2010; Frasa et al., 2012; Fukuda, 2011). TBC domain is a conserved catalytic domain that consists of ~200 amino acid residues. Since the identification of the TBC domain in yeast Rab GAPs (Albert et al., 1999), a number of TBC proteins have been characterized in mammalian cells. Based on the sequence homology of the TBC domain, more than 40 TBC proteins are predicted to exist in humans. Some TBC proteins have been demonstrated to act on specific Rabs and to regulate specific trafficking steps and cellular processes same as their respective target Rabs (Davey et al., 2012; Fuchs et al., 2007; Goueli et al., 2012; Haas et al., 2005, 2007; Hsu et al., 2010; Itoh and Fukuda, 2006; Itoh et al., 2011; Peralta et al., 2010; Yoshimura et al., 2007). The most TBC proteins use a dual Arg-Gln-finger catalytic mechanism to inactivate Rab substrates (Du and Novick, 2001; Pan et al., 2006) that is largely different from the single Arg finger mechanism used by many other small GTPase GAPs.

G-protein-coupled receptors (GPCRs) modulate a wide variety of physiological and pathological functions and are the therapeutic targets of approximately one-third of the drugs on the market (Hauser et al., 2017; Hilger et al., 2018; Pierce et al., 2002). For most GPCRs, the plasma membrane represents the most important functional destination where they bind to their respective ligands and activate cognate heterotrimeric G proteins, arrestins, and other signaling proteins that in turn activate downstream effectors. However, as compared with extensively studied internalization, recycling, and degradation pathways (Hanyaloglu and von Zastrow, 2008; Marchese et al., 2008), the molecular mechanisms underlying the anterograde transport of nascent GPCRs to the plasma membrane, *en route* from the endoplasmic reticulum (ER) where they are synthesized and through the Golgi where the receptors attain fully matured statues, remain poorly understood.

A number of Rab GTPases have been well studied to regulate the cell surface transport, internalization, recycling, degradation, and nuclear translocation of a number of GPCRs (Bhosle et al., 2016; Dale et al., 2004; Fan et al., 2003; Filipeanu et al., 2006; Li et al., 2012, 2017; Wang and Wu, 2012; Wu et al., 2003). However, the functions of TBC proteins in any trafficking processes of the GPCR superfamily have not been investigated. In this study, we identify six TBC proteins to activity-dependently regulate the anterograde ER-Golgi-cell surface traffic of GPCR members. We also show that TBC1D6 controls the post-Golgi transport of GPCRs via inactivating Rab26 and inhibiting Rab26 interaction with the receptors. These results provide a previously unappreciated function of TBC proteins, by virtue of their abilities to regulate both Rab GTPase activation and Rab-GPCR interaction, in the intracellular trafficking of GPCR members and reveal regulatory mechanisms of targeting to the functional destination of nascent GPCRs, a poorly explored area in the study of the GPCR superfamily.

## RESULTS

### Six TBC Proteins Regulate the Cell Surface Delivery and Signaling of α_2B_-Adrenergic Receptor (AR) in an Activity-Dependent Manner

**Figure 1.**
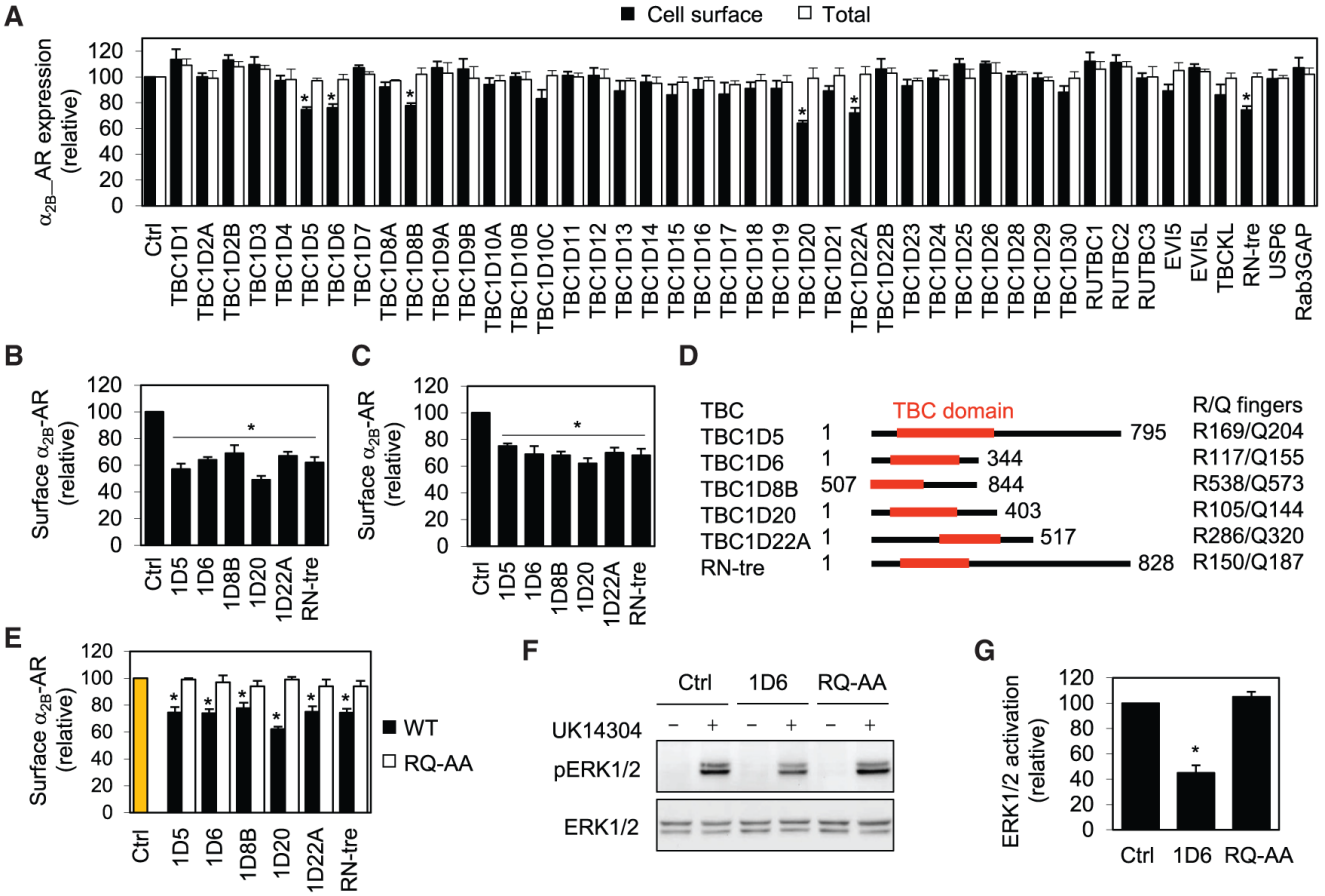
Six TBC Proteins Regulate the Cell Surface Expression of α_2B_-AR in an Activity-Dependent Fashion (A) Screening for TBC proteins involved in the surface transport of α_2B_-AR. HEK293 cells stably expressing HA-α_2B_-AR were transfected with the control vector pEGFP-C1 (Ctrl) or individual GFP-TBC proteins. The cell surface expression of α_2B_-AR was determined by intact cell ligand binding using [^3^H]-RX821002. The mean value of specific radioligand binding was 23,432 ± 1,432 cpm from cells transfected with the control vector. (B) Inhibition of the surface transport of inducibly expressed α_2B_-AR by TBC proteins. Stable HEK293 cells inducibly expressing HA-α_2B_-AR were transfected with pEGFP-C1 (Ctrl) or GFP-TBC proteins, and the cell surface expression of the receptor was measured by radioligand binding after incubation with doxycycline (40 ng/mL) for 24 h. In a typical experiment, the maximal specific binding of ^3^H-RX821002 are 21,562 cpm/well (24-well dish) in control cells. (C) Inhibition of the surface expression of endogenous α_2_-AR by TBC proteins in MCF-7 cells. The mean value of specific ligand binding was 762 ± 143 cpm in control cells. (D) A diagram showing the locations of the TBC domain (red) in six TBC proteins. The right panel indicates the exact positions of the Arg and Gln fingers. (E) Effect of six TBC proteins and their RQ-AA mutants on the cell surface expression of α_2B_-AR as measured by intact cell ligand binding. (F) Effect of TBC1D6 and its RQ-AA mutant on α_2B_-AR-mediated ERK1/2 activation. HEK293 cells stably expressing α_2B_-AR were transfected with the pEGFP-C1 vector (Ctrl) or GFP-TBC1D6. The cells were stimulated with UK14304 at 1 μM for 5 min and ERK1/2 activation was determined by immunoblotting. (G) Quantitative data shown in (F). The quantitative data are presented as mean ± SE (n = 3–4). *p < 0.05 versus Ctrl.

As an initial approach to study the possible functions of TBC proteins in regulating GPCR trafficking, we used α_2B_-AR, a prototypic GPCR, as a model to screen for TBC proteins involved in the anterograde cell surface transport using stable HEK293 cells expressing N-terminal hemagglutinin (HA)-tagged α_2B_-AR. Forty-three TBC domain-containing proteins and Rab3GAP, the known Rab3 GAP without a TBC domain, were tagged with GFP and their expression was visualized by confocal microscopy (Figure S1A). The effects of TBC proteins on the cell surface transport of α_2B_-AR at steady state were measured by intact live cell ligand binding using the cell nonpermeable radioligand ^3^H-RX821002. This strategy identified six TBC proteins, namely TBC1D5, TBC1D6, TBC1D8B, TBC1D20, TBC1D22A, and RN-tre, which significantly inhibited the cell surface numbers of α_2B_-AR by ~30% as compared with cells expressing the control vector pEGFP-C1 (Figure 1A). In contrast, other 37 TBC proteins and Rab3GAP did not have clear effects on the cell surface expression of α_2B_-AR (Figure 1A). The expression of TBC proteins and Rab3GAP did not influence the total expression of α_2B_-AR as measured by flow cytometry following staining with HA antibodies in permeabilized cells (Figure 1A). Furthermore, expression of arrestin-3(201-409), a dominant-negative mutant of arrestin-3 that mediates agonist-stimulated internalization of a number of GPCRs including α_2B_-AR (DeGraff et al., 1999), did not reverse the effect of TBC proteins on the cell surface expression of α_2B_-AR (Figure S1B), suggesting that the reduction of α_2B_-AR expression at the cell surface induced by TBC proteins was not caused by enhanced internalization of the receptor.

We then determined the effect of the six TBC proteins on the cell surface transport of newly synthesized α_2B_-AR in inducible HEK293 cells generated by using the Tet-On 3G tetracycline inducible gene expression system. The inducible cells were first transiently transfected with individual TBC proteins and then incubated with doxycycline at a concentration of 40 ng/mL for 24 h to achieve the maximal cell surface expression of the receptor (Li et al., 2017; Zhang et al., 2016a). Expression of each of the six TBC proteins significantly attenuated the cell surface expression of ±_2B_-AR (Figure 1B). Similarly, transient expression of these TBC proteins inhibited the cell surface expression of endogenous ±_2B_-AR by 30%–50% in MCF-7 cells (Figures 1C and S1C). These data demonstrate that the TBC proteins TBC1D5, TBC1D6, TBC1D8B, TBC1D20, TBC1D22A, and RNtre are able to regulate the cell surface transport of α_2B_-AR.

The six TBC proteins identified above to regulate the cell surface transport of α_2B_-AR are canonical TBC proteins containing two catalytic residues, an Arg finger and a Gln finger (Figure 1D). To define if their inhibitory effects on α_2B_-AR transport were specifically mediated through inactivating Rab GTPases, we compared the effects of wild type TBC proteins with their respective catalytically inactive mutants in which both the Arg and Gln fingers were mutated to Ala (RQ-AA). Confocal microscopy revealed that RQ mutations did not alter the subcellular localization of these TBC proteins (Figure S1D). Immunoblotting showed that, as compared with their RQ-AA mutants, the six TBC proteins were expressed at similar levels with predicted apparent molecular weights (Figure S1E). In marked contrast to wild type TBC counterparts that blocked the cell surface transport of α_2B_-AR, expression of the RQ-AA mutants had no significant effects on α_2B_-AR expression at the cell surface measured by radioligand binding (Figure 1E). These data suggest that the function of these TBC proteins in regulating α_2B_-AR transport is dependent on their GAP activity.

We next addressed the question if TBC protein-induced reduction of the cell surface α_2B_-AR could result in a concomitant defective signaling. α_2B_-AR couples to the Gi/Go family G proteins, and its activation has been well shown to stimulate the mitogen-activated protein kinases (MAPK) ERK1/2, inhibit adenylyl cyclases, and suppress voltage-gated calcium channel (Dong et al., 2011). The activation of ERK1/2 was chosen as a functional readout, and TBC1D6 was chosen as a representative TBC protein to be studied. Consistent with its ability to inhibit α_2B_-AR cell surface transport, the expression of TBC1D6 significantly attenuated ERK1/2 activation in response to UK14304 stimulation by 55% in cells stably expressing α_2B_-AR, whereas its mutant RQ-AA had no effect (Figures 1F and 1G). To eliminate the possibility that the effect of TBC1D6 on the MAPK activation was mediated through the inhibition of the activation of G proteins and/or other signaling molecules, we measured the effect of TBC1D6 on MAPK activation by 1,3-bis(4-amino-2-methylquinolin-6-yl)urea (NSC12155) that activates MAPK via enhancing the release of Gβγ from the Gαβγ trimers (Surve et al., 2014). Treatment with NSC12155 remarkably activated MAPK that was not affected by expression of TBC1D6 and its inactive mutant (Figures S1F and S1G). These data suggest that TBC1D6 does not influence the activation of G proteins and other signaling molecules downstream of G proteins involved in the MAPK activation, and TBC proteins regulate not only α_2B_-AR trafficking but also its function in an activity-dependent fashion.

### Specific TBC Proteins Control the ER-to-Golgi and the Golgi-to-Plasma Membrane Transport of α_2B_-AR

**Figure 2.**
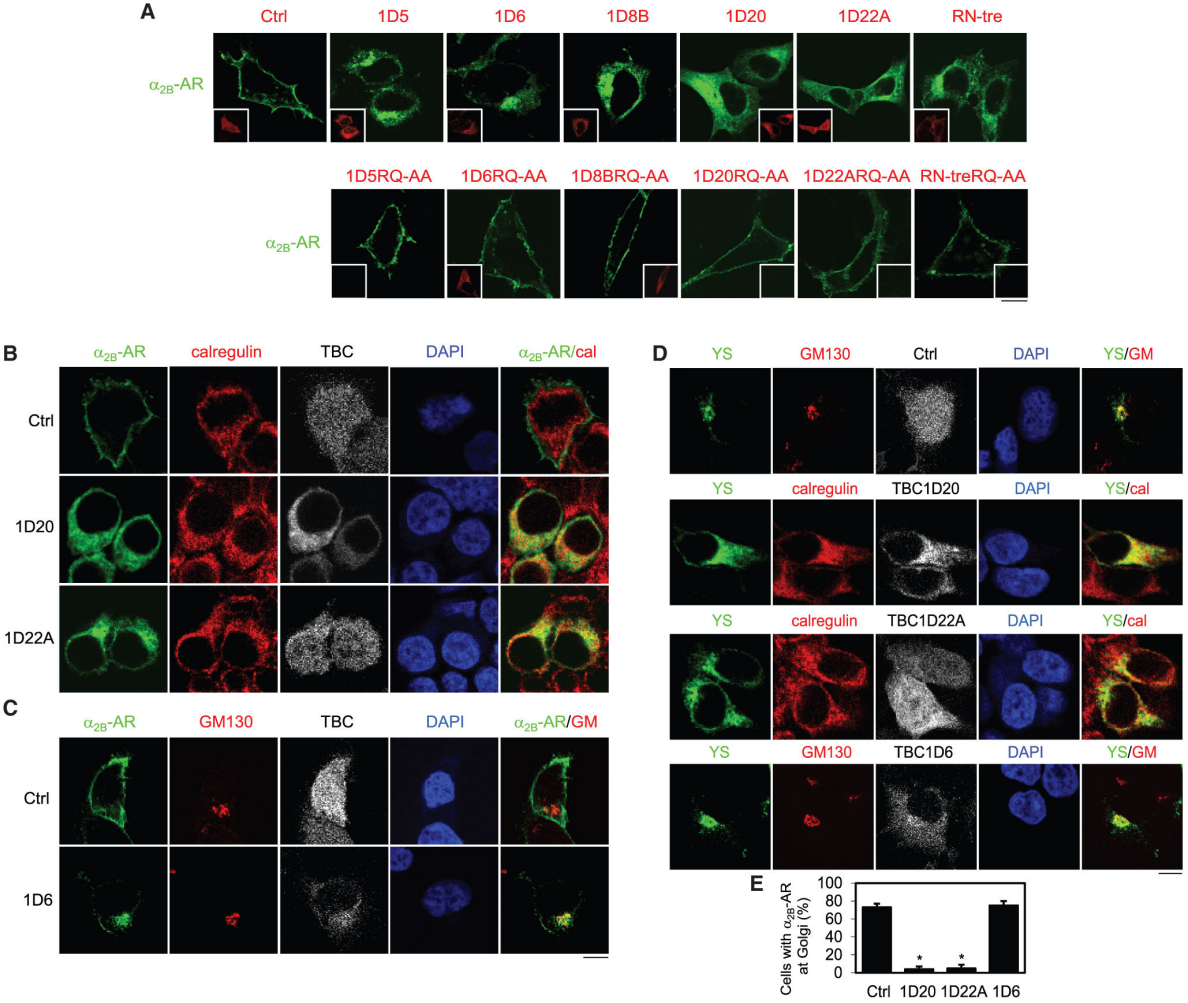
Inhibition of α_2B_-AR Transport from the ER through the Golgi to the Cell Surface by TBC Proteins (A) Effect of the six TBC proteins and their inactive RQ-AA mutants on the subcellular distribution of α_2B_-AR. HEK293 cells were transfected with α_2B_-AR-GFP together with the vector dsRed-C1 (Ctrl) or individual dsRed-TBC proteins. The subcellular distribution of α_2B_-AR was revealed by confocal microscopy. Insets show the expression of individual TBC proteins. (B) Colocalization of α_2B_-AR with the ER marker calregulin in cells expressing TBC1D20 and TBC1D22A. HEK293 cells were transfected with α_2B_-AR-GFP together with dsRed-C1 (Ctrl) or individual dsRed-tagged TBC proteins and then stained with calregulin antibodies. (C) Colocalization of α_2B_-AR with the Golgi marker GM130 in cells expressing TBC1D6. HEK293 cells were transfected with α_2B_-AR-GFP together with dsRed-C1 (Ctrl) or individual dsRed-tagged TBC proteins and then stained with GM130 antibodies. (D) Effect of TBC proteins on the ER-to-Golgi transport of the α_2B_-AR YS mutant. HEK293 cells were transfected with GFP-tagged YS mutant together with dsRed-C1 (Ctrl) or dsRed-TBC proteins. The cells were then stained with GM130 antibodies in cells expressing control vector and TBC1D6 or with calregulin antibodies in cells expressing TBC1D20 and TBC1D22A. (E) Quantitative data shown in (D). Approximately 100 cells were counted in each experiment. The quantitative data are mean ± SE (n = 3). *p < 0.05 versus Ctrl. The images shown are representatives of at least 3 experiments. Scale bars, 10 μm.

To confirm the effects of the six TBC proteins on the cell surface expression of α_2B_-AR measured by radioligand binding, confocal microscopy was utilized to directly visualize subcellular distribution of α_2B_-AR in cells expressing individual TBC proteins. For this purpose, a2B-AR was tagged with GFP, whereas the six TBC proteins were tagged with dsRed. As expected, GFP-tagged α_2B_-AR was robustly expressed at the cell surface in cells transfected with the control vector dsRed. The expression of TBC1D5, TBC1D6, TBC1D8B, TBC1D20, TBC1D22A, and RN-tre each caused a marked reduction in the cell surface presentation of α_2B_-AR that was accompanied by an extensive accumulation of the receptors in the perinuclear region, whereas the expression of TBC mutants did not alter the cell surface expression of α_2B_-AR (Figure 2A). The effects of the six TBC proteins on the subcellular distribution of α_2B_-AR were markedly different. Whereas the subcellular localizations of α_2B_-AR were similar in cells expressing TBC1D5, TBC1D6, and TBC1D8, they are apparently different from those in cells expressing TBC1D20, TBC1D22A, and RN-tre (Figure 2A).

To define the intracellular compartments in which individual TBC proteins regulate α_2B_-AR transport, we first studied the colocalization of α_2B_-AR with different intracellular organelle marker proteins in cells expressing TBC1D6, TBC1D20, and TBC1D22A. α_2B_-AR was strongly colocalized with the ER marker calregulin in cells expressing TBC1D20 and TBC1D22A (Figure 2B), whereas it was clearly colocalized with the *cis*-Golgi marker GM130 in cells expressing TBC1D6 (Figure 2C). These data suggest that TBC1D20 and TBC1D22A control α_2B_-AR transport from the ER to the Golgi, whereas likely TBC1D6 regulates α_2B_-AR transport from the Golgi to the plasma membrane.

We next took advantage of our previously characterized Golgi-localized YS mutant of α_2B_-AR (Dong and Wu, 2006) and determined if TBC1D6, TBC1D20, and TBC1D22A could block its transport from the ER to the Golgi. The mutant YS-AA was mainly retained in the Golgi in cells expressing the control vector dsRed and TBC1D6, but it was clearly arrested in the ER, unable to export to the Golgi, in cells expressing TBC1D20 and TBC1D22A (Figures 2D and 2E). This further indicates a role for TBC1D20 and TBC1D22A in the ER-to-Golgi transport of α_2B_-AR.

### Multiple TBC Proteins Selectively Modulate the Cell Surface Export of GPCRs, but Not Other Plasma Membrane Proteins

**Figure 3.**
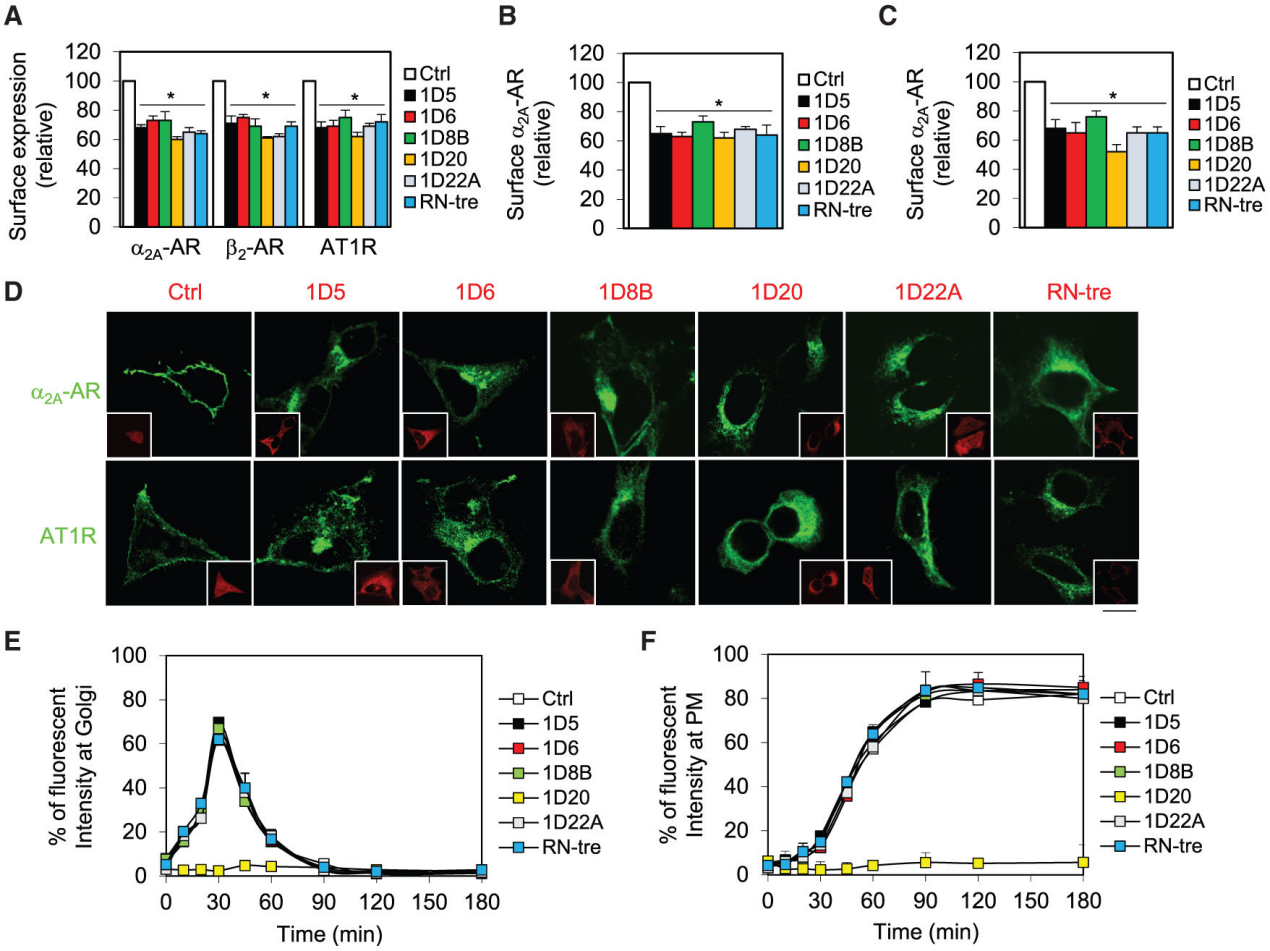
Effect of Six TBC Proteins on the Cell Surface Traffic of GPCRs and Other Plasma Membrane Proteins (A) Effect of TBC proteins on the surface expression of α_2A_-AR, β_2_-AR, and AT1R. HEK293 cells were transfected with individual receptors together with pEGFP-C1 (Ctrl) or GFP-TBC proteins and the cell surface expression of each receptor was measured by radioligand binding or flow cytometry. (B) Inhibition of the surface transport of inducibly expressed α_2A_-AR by TBC proteins. Stable HEK293 cells inducibly expressing HA-α_2A_-AR were transfected with pEGFP-C1 (Ctrl) or GFP-TBC proteins and the cell surface expression of the receptor was measured by radioligand binding after doxycycline induction for 24 h. In a typical experiment, the maximal specific binding of ^3^H-RX821002 are 24,805 cpm/well (24-well dish) in control cells. (C) Inhibition of the surface expression of endogenous α_2A_-AR by TBC proteins in HT29 cells. The mean value of specific ligand binding was 892 ± 187 cpm in control cells. (D) Effect of six TBC proteins on the subcellular distribution of α_2A_-AR and AT1R. HEK293 cells were transfected with α_2A_-AR-GFP or AT1R-GFP together with the vector dsRed-C1 (Ctrl) or individual dsRed-TBC proteins. The subcellular distribution of the receptors was revealed by confocal microscopy. (E) Effect of TBC proteins on the expression of VSVG at the Golgi. HEK293 cells were transfected with VSVGtsO45-GFP together with dsRed-C1 (Ctrl) or dsRed-tagged individual TBC proteins. The cells were cultured at 40°C for 24 h (0 min) and then shifted to 32°C for 10, 20, 30, 45, 60, 90, 120, and 180 min. The expression of VSVG at the Golgi was measured by fluorescent intensity in a total of 25–30 cells. (F) Effect of TBC proteins on the expression of VSVG at the plasma membrane (PM) as described in (E). The data are mean ± SE (n = 3–7). *p < 0.05 versus Ctrl. The images are representatives of 3 experiments. Scale bar, 10 μm.

We then sought to determine if the six TBC proteins identified above were able to regulate the other three family A GPCRs, α_2A_-AR, β_2_-AR, and angiotensin II type 1 receptor (AT1R), in transient expression systems. Similar to their effects on α_2B_-AR, the expression of TBC1D5, TBC1D6, TBC1D8B, TBC1D20, TBC1D22A, and RN-tre each significantly inhibited the cell surface expression of α_2A_-AR, β_2_-AR, and AT1R by 25%–45% as measured by radioligand binding or flow cytometry (Figure 3A). The expression of these six TBC proteins also significantly attenuated the cell surface expression of inducibly expressed α_2A_-AR in HEK293 cells (Figure 3B). Furthermore, the expression of each of these six TBC proteins reduced the cell surface expression of endogenous α_2A_-AR in HT-29 cells (Figures 3C and S2A).

We then studied the effect of the six TBC proteins on the sub-cellular localization of α_2A_-AR and AT1R. The expression of each of the six TBC proteins arrested both receptors in the perinuclear region revealed by confocal microscopy (Figure 3D). In contrast, among the six TBC proteins, only TBC1D20 was found to activity-dependently inhibit the cell surface expression of epidermal growth factor receptor (EGFR) as measured by quantification of fluorescent intensity, whereas TBC1D5, TBC1D6, TBC1D8B, TBC1D22A, and RN-tre had no effect (Figures S2B and S2C). Consistently, expression of TBC1D6 had no effect of the activation of MAPK by EGF (Figure S2D and S2E).

Similar to EGFR, the transport of vesicular stomatitis virus glycoprotein (VSVG) using its temperature-sensitive mutant (VSVGtsO45) from the ER to the Golgi and to the cell surface was markedly inhibited by TBC1D20, but not its RQ-AA mutant and other five TBC proteins (Figures 3E, 3F, and S2F). These data suggest that the actions of TBC1D5, TBC1D6, TBC1D8B, TBC1D22A, and RN-tre on the transport of GPCRs are likely specific.

**Figure 4.**
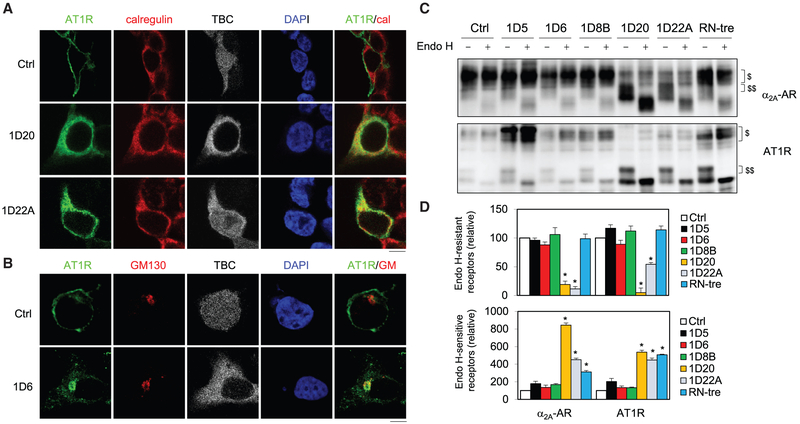
Effect of TBC Proteins on the Subcellular Distribution and Glycosylation of GPCRs (A) Colocalization of AT1R with calregulin in cells expressing TBC1D20 and TBC1D22A. (B) Colocalization of AT1R with GM130 in cells expressing TBC1D6. (C) Deglycosylation of α_2A_-AR (upper panel) and AT1R (lower panel) by Endo H treatment. $ indicates Endo H-resistant bands and $$ Endo H-sensitive bands. (D) Quantitative data showing the effect of TBC proteins on the formation of Endo H-resistant (upper panel) and Endo H-sensitive glycosylation (lower panel) of the receptors. The quantitative data are mean ± SE (n = 3–5). *p < 0.05 versus Ctrl. The images shown are representatives of 4 experiments. Scale bars, 10 μm.

Confocal microscopy revealed that, similar to α_2B_-AR, AT1R was remarkably colocalized with calregulin in cells expressing TBC1D20 and TBC1D22A (Figure 4A), whereas it was extensively colocalized with GM130 in cells expressing TBC1D6 (Figure 4B).

We next defined the effect of the six TBC proteins on the conversion from simple to complex glycosylation of GPCRs that reflects the transport of the receptors from the ER to the Golgi. α_2B_-AR does not have N-linked glycosylation sites, whereas α_2A_-AR and AT1R possess 2 and 3 N-linked glycosylation sites at their N termini, respectively, and therefore were chosen to be studied. The expression of TBC1D20, TBC1D22A, and RN-tre markedly enhanced the formation of endoglycosidase (Endo) H-sensitive glycosylation of both receptors (Figures 4C and 4D). However, TBC1D20 and TBC1D22A, but not RN-tre, inhibited the acquisition of complex N-linked sugars (Endo H-resistant) (Figures 4C and 4D) that was further confirmed by PNGase F treatment to remove both simple and complex glycosylation (Figure S3). In contrast, TBC1D5, TBC1D6, and TBC1D8B did not affect both Endo H-sensitive and -resistant glycosylation of the receptors. These data further suggest that TBC1D20, TBC1D22A, and RN-tre, but not TBC1D5, TBC1D6, and TBC1D8B, may play a role in GPCR transport from the ER to the Golgi.

### TBC1D6 Is a Specific GAP for Rab26 and Regulates Rab26 Interaction with GPCRs

Our previous studies have demonstrated that Rab26 and three Golgi-localized, γ-adaptin ear domain homology, ADP ribosylation factor-binding proteins (GGAs) regulate the Golgi-to-plasma membrane transport of GPCRs (Li et al., 2012; Zhang et al., 2016a, 2016b). They all directly interacted with α_2B_-AR, specifically its third intracellular loop (ICL3), and the RRR motif was identified as the GGA3-binding motif in the ICL3 (Figure S4A). However, mutation of the RRR motif did not affect Rab26 interaction with the ICL3 (data not shown), and progressive deletion of the ICL3 failed to identify a domain interacting with Rab26 (Figures S4B and S4C), suggesting that the conformation of the loop may be important for Rab26 interaction. Furthermore, small interfering RNA (siRNA)-mediated depletion of Rab26 inhibited the cell surface transport of α_2A_-AR, α_2B_-AR, and an α_2B_-AR mutant in which the RRR motif was mutated to Ala, whereas knockdown of GGA3 by siRNA only attenuated the cell surface expression of a2B-AR, but not the RRR mutant and α_2A_-AR (Figure S4D). In addition to α_2B_-AR, the α_2A_-AR ICL3 and the AT1R C terminus also strongly bound to Rab26 as measured in GST fusion protein pull-down assays (Figure S4E). These data demonstrate that Rab26 and GGAs differentially regulate GPCR transport (Figure S4F). These data also suggest that Rab26 and GGAs likely control the post-Golgi transport of GPCRs through distinct routes.

**Figure 5.**
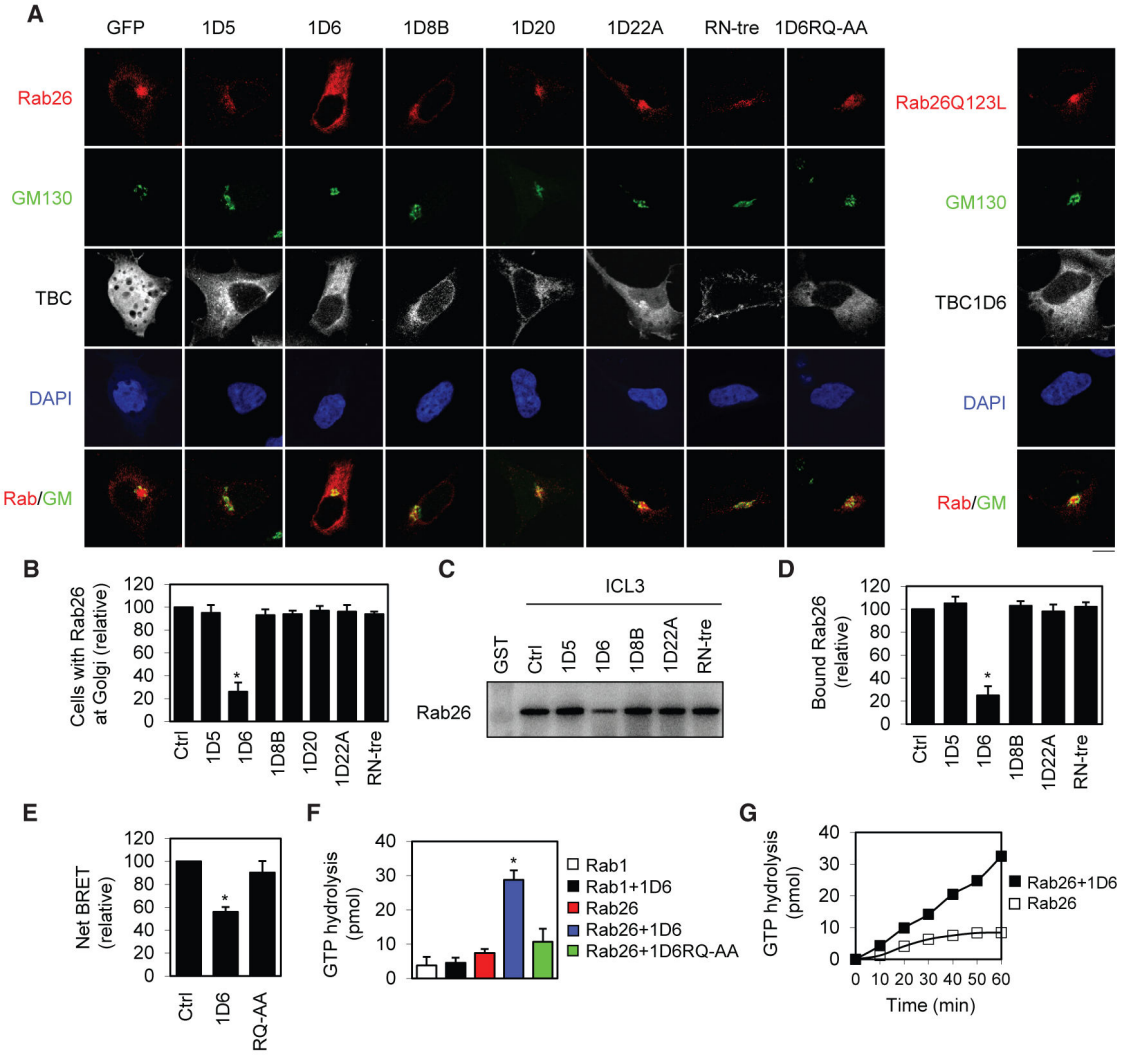
Effect of TBC Proteins on the Subcellular Localization, Interaction with α_2B_-AR, and GTP Hydrolysis of Rab26 (A) Effect of the six TBC proteins on the subcellular localization of Rab26. HEK293 cells were transfected with individual GFP-tagged TBC proteins together with dsRed-tagged Rab26 or the active mutant Rab26Q123L and then stained with GM130 antibodies. (B) Quantitative data shown in (A). The data are percentage of cells with Rab26 expression at the Golgi with a total of 50 cells counted in each experiment. (C) Effect of the six TBC proteins on Rab26 interaction with the ICL3 of α_2B_-AR in GST fusion protein pull-down assays. (D) Quantitative data shown in (C). (E) Effect of TBC1D6 and its inactive mutant on Rab26 interaction with full-length α_2B_-AR in BRET assays. (F) Effect of TBC1D6 on GTP hydrolysis by Rab26 and Rab1 measured in GAP assays. GST-tagged Rabs were loaded with [γ-^32^P]-GTP and then incubated with TBC1D6 or its QR-AA mutant. (G) Time-dependent action of TBC1D6 on GTP hydrolysis by Rab26. Similar results were obtained in 2 different experiments. The quantitative data are mean ± SE (n = 3–5). *p < 0.05 versus Ctrl. The images shown are representatives of 3 experiments. Scale bars, 10 μm.

Because the GAPs for Rab26 have not been identified, we hypothesized that at least one of the six TBC proteins identified above was a specific GAP for Rab26. To test this hypothesis, we first determined the effect of the six TBC proteins on the sub-cellular localization of Rab26 by confocal microscopy. Rab26, as well as its constitutively active mutant Q123L, localizes to the Golgi and partially colocalizes with GM130, whereas its inactive mutants are mainly expressed in cytoplasm (Figure 5A) (Li et al., 2012). The expression of TBC1D6 markedly disrupted the Golgi localization of Rab26 but had no effect on the mutant Rab26Q123L. In contrast, the expression of TBC1D6 mutant RQ-AA, as well as other five TBC proteins, did not affect the Golgi localization of Rab26 (Figures 5A and 5B). These data suggest that TBC1D6 may enhance the transition of Rab26 from an active state to an inactive state in cell.

As Rab26 interaction with the ICL3 as well as full-length α_2B_-AR was shown to highly depend on its activation (Li et al., 2012), these protein-protein interactions provide unique assays to measure Rab26 activation. The ICL3 of α_2B_-AR was generated as fusion proteins and then incubated with the total cell lysates prepared from cells expressing individual TBC proteins. As TBC1D20 has been well defined as a GAP for Rab1 and Rab2 (Haas et al., 2007; Ishida et al., 2012; Nachmias et al., 2012; Nevo-Yassaf et al., 2012; Sklan et al., 2007), it was not studied in this experiment. The expression of TBC1D6 strongly inhibited Rab26 interaction with the ICL3, whereas TBC1D5, TBC1D8B, TBC1D22A, and RN-tre had no significant effects (Figures 5C and 5D). In live cell bioluminescence resonance energy transfer (BRET) assays, the expression of TBC1D6, but not its inactive RQ-AA mutant, significantly reduced the BRET between α_2B_-AR-Rluc and Rab26-venus (Figure 5E). These data demonstrate that TBC1D6 not only inactivates Rab26 but also attenuates its interaction with α_2B_-AR.

The GAP assay was then utilized to directly measure the ability of TBC1D6 to accelerate the GTP hydrolysis by Rab GTPases. TBC1D6, but not its QR-AA mutant, markedly enhanced the GTP hydrolysis by Rab26, without affecting GTP hydrolysis by Rab1 (Figure 5F). The enhancement of Rab26-mediated GTP hydrolysis by TBC1D6 was in a time-dependent fashion (Figure 5G). These data demonstrate that TBC1D6 is a specific GAP for Rab26.

### TBC1D6 Physically Associates with Rab26

**Figure 6.**
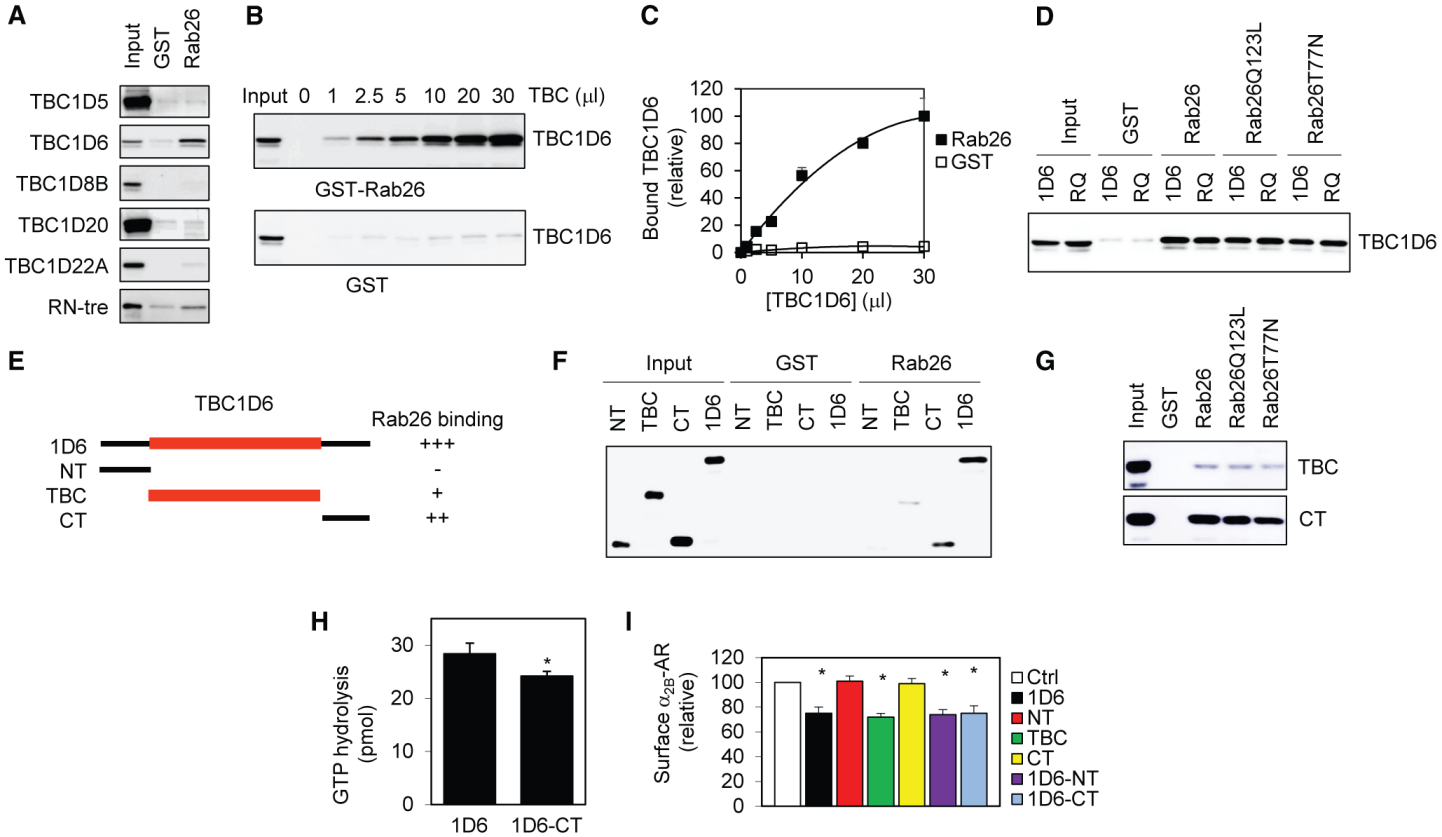
TBC1D6 Interaction with Rab26 and Identification of the Rab26-Binding Domains of TBC1D6 (A) Interaction of TBC1D6 with Rab26 in GST-Rab26 fusion protein pull-down assays. (B) Dose dependent interaction of TBC1D6 with Rab26. (C) Quantitative data shown in (B). (D) Interaction of TBC1D6 and its catalytically inactive RQ-AA mutant with Rab26 and its GTP-bound (Q123L) and GDP-bound (T77N) mutants in GST fusion protein pull-down assays. (E) Summary of Rab26 binding to full-length TBC1D6 and its N terminus (NT), TBC domain (TBC), and C terminus (CT) as shown in (F). (F) A representative blot showing Rab26 interaction with different TBC1D6 domains. (G) Interaction of the CT and the TBC domain of TBC1D6 with Rab26 and its GTP-bound (Q123L) and GDP-bound (T77N) mutants in GST fusion protein pull-down assays. (H) Effect of TBC1D6 and its mutant lacking the C terminus (1D6-CT) on GTP hydrolysis by Rab26 measured in GAP assays. (I) Effect of TBC1D6 domains on the cell surface transport of α_2B_-AR. TBC1D6 and different domains were transiently expressed in HEK293 cells stably expressing α_2B_-AR. The cell surface expression of α_2B_-AR was measured by intact cell ligand binding. Immunoblots shown are representatives of at least 3 experiments. Input represents 10% of total amount used in each reaction. The quantitative data shown are mean ± SE (n = 3–5). *p < 0.05 versus Ctrl.

Previous studies have demonstrated that TBC proteins may directly interact with their target Rabs (Dabbeekeh et al., 2007; Gallo et al., 2014; Haas et al., 2005; Itoh et al., 2006). We then sought to determine if Rab26 was able to physically associate with TBC1D6 in GST-Rab26 fusion protein pull-down assays. TBC1D6, but not TBC1D5, TBC1D18B, TBC1D20, TBC1D22A, and RN-tre, interacted with Rab26 (Figure 6A). The interaction was dependent upon TBC1D6 concentration (Figures 6B and 6C).

To determine if the interaction between Rab26 and TBC1D6 was dependent on their activation, Rab26 and its GTP- and GDP-bound mutants were generated as GST fusion proteins and incubated with the total cell lysates expressing TBC1D6 or its inactive RQ-AA mutant. Rab26 and its mutants all strongly bound to TBC1D6 and RQ-AA mutant, and the binding affinities between wild type and mutated Rab26 and TBC1D6 are seemly similar (Figure 6D). These data suggest that interaction between Rab26 and TBC1D6 is independent of their activation statuses.

To identify the domains that mediate TBC1D6 interaction with Rab26, the N terminus, the TBC domain, and the C terminus of TBC1D6 were tagged with GFP, expressed in cells (Figure S5), and incubated with GST-Rab26 fusion proteins. Both the C terminus and the TBC domain, but not the N terminus, clearly interacted with Rab26. The C-terminal interaction with Rab26 was stronger than that of the TBC domain, but both were weaker than full-length TBC1D6 (Figures 6E and 6F). Similar to full-length TBC1D6, the interaction of TBC domain and C terminus with Rab26 is independent of Rab26 activation (Figure 6G). These data suggest that TBC1D6 has multiple Rab26-binding sites.

As the C terminus strongly bound to Rab26, we sought to determine its role in regulating TBC1D6 function. We first determined if the C terminus was required for the GAP activity of TBC1D6 toward Rab26. The hydrolysis of GTP by Rab26 was moderately but significantly weaker in the presence of TBC1D6 mutant lacking the C terminus (1D6-CT) than in the presence of full-length TBC1D6 as measured in *in vitro* GAP assays (Figure 6H). These data suggest that the C-terminal binding to Rab26 is able to enhance the GAP activity of TBC1D6. We then determined the role of different domains of TBC1D6 in GPCR transport. The expression of full-length TBC1D6, TBC domain alone, TBC-CT, and TBC1D6 mutant lacking the N-terminal portion (1D6-NT) similarly inhibited the cell surface transport of α_2B_-AR, whereas the N and C termini had no effect (Figures 6I and S5). These data suggest that the TBC domain itself is sufficient to inhibit GPCR transport and that, although the C terminus is able to interact Rab26 and enhance the GAP activity of TBC1D6, it may not significantly contribute to the regulation of GPCR cell surface export at steady state.

### Depletion of TBC1D6 Inhibits the Cell Surface Expression and Signaling of GPCRs

**Figure 7.**
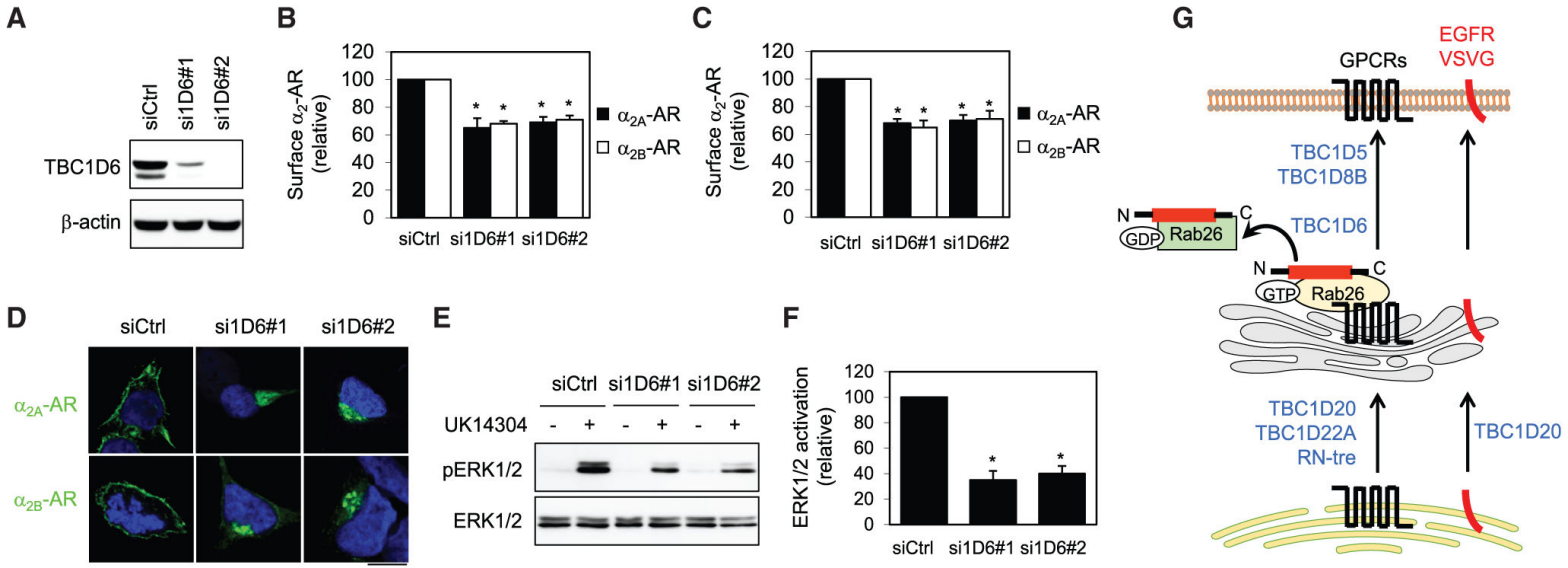
Effect of siRNA-Mediated Depletion of TBC1D6 on the Cell Surface Transport and Signaling of GPCRs (A) Western blot analysis of siRNA-mediated knockdown of TBC1D6 in HEK293 cells. (B) Effect of TBC1D6 siRNA on the surface expression of stably expressed α_2A_-AR and α_2B_-AR. (C) Effect of TBC1D6 siRNA on the surface expression of inducibly expressed α_2A_-AR and α_2B_-AR. (D) Effect of TBC1D6 siRNA on the subcellular localization of α_2A_-AR and α_2B_-AR. HEK293 cells were transfected with siRNA targeting TBC1D6 together with GFP-tagged α_2A_-AR or α_2B_-AR. (E) Effect of TBC1D6 siRNA on α_2B_-AR-mediated ERK1/2 activation in HEK293 cells stably expressing α_2B_-AR. (F) Quantitative data shown in (E). (G) A model depicting the functions of six TBC proteins, as well as Rab26, in the forward ER-Golgi-plasma membrane transport of GPCRs and other plasma membranes (see text for detail). The quantitative data are mean ± SE (n = 3–6). *p < 0.05 versus Ctrl. The images shown are representatives of 3 experiments. Scale bar, 10 μm.

The function of TBC1D6 in regulating GPCR transport was further studied by siRNA-mediated depletion strategy. Due to the low expression level of endogenous TBC1D6 in HEK293 cells and the quality of TBC1D6 antibodies, we measured the ability of TBC1D6 siRNA to deplete transiently expressed GFP-tagged TBC1D6. Two siRNA targeting TBC1D6 markedly knocked down GFP-TBC1D6 by more than 75% when compared with cells transfected with control siRNA (Figure 7A). Interestingly, similar to its overexpression, siRNA-mediated knockdown of TBC1D6 significantly attenuated the cell surface numbers of stably expressed α_2A_-AR and α_2B_-AR in HEK293 cells (Figure 7B). siRNA targeting TBC1D6 also attenuated the cell surface expression of inducibly expressed α_2A_-AR and α_2B_-AR (Figure 7C). Both α_2_-ARs were clearly arrested in the perinuclear region, most likely the Golgi, in cells expressing TBC1D6 siRNA (Figure 7D). Furthermore, expression of siRNA targeting TBC1D6 markedly inhibited α_2B_-AR-mediated ERK1/2 activation by ~65% as compared to cells expressing control siRNA (Figures 7E and 7F). These data suggest that the normal expression level of TBC1D6 is an essential, limiting factor for the anterograde transport of α_2_-ARs.

Our preceding data demonstrate that overexpression and depletion of TBC1D6 produce an inhibitory effect on the cell surface transport of GPCRs, and TBC1D6 is likely a GAP for Rab26, suggesting that proper GDP/GTP cycling of Rab26 may be a crucial event for membrane trafficking of GPCRs. To further test this possibility, we determined the effect of transient expression of GTP-bound mutant Rab26Q123L and GDP-bound mutant Rab26T77N on the cell surface transport of several GPCRs. The expression of Rab26Q123L and Rab26T77N similarly inhibited the cell surface transport of α_2A_-AR, α_2B_-AR, β_2_-AR, and AT1R (Figure S6A), but Rab26T77N did not affect the ER-Golgi-cell surface transport of VSVG (Figures S6B-S6D). As TBC1D20 was suggested to be a GAP for Rab33 (Fuchs et al., 2007; Pan et al., 2006), we determined if Rab33a and Rab33b were involved in the cell surface transport of two GPCRs, α_2A_-AR and α_2B_-AR. In contrast to Rab26, expression of Rab33a and Rab33b and their dominant-negative mutants had no significant effects on the cell surface expression of α_2A_-AR and α_2B_-AR (Figure S6E), suggesting that the function of TBC1D22A in GPCR trafficking is unlikely mediated via the inactivation of Rab33.

## DISCUSSION

In this study, we have investigated the possible functions of RabGAPs in GPCR trafficking and identified six TBC proteins, namely TBC1D5, TBC1D6, TBC1D8B, TBC1D20, TBC1D22A, and RN-tre, which clearly regulate the anterograde cell surface transport of multiple GPCRs. This function of RabGAPs was first identified in a screening assay in which we measured the effect of almost all TBC proteins identified thus far on the cell surface expression of stably expressed α_2B_-AR at steady state by radioligand binding of live cells, which was further confirmed by different cell systems in which the receptor was expressed transiently, inducibly, and endogenously. The function of these six TBC proteins in the cell surface transport of α_2B_-AR was also supported by confocal microscopy to directly visualize the sub-cellular localization of the receptor and by receptor-mediated signaling measured as ERK1/2 activation. In addition to a2B-AR, the cell surface expression of several other family A GPCRs are similarly affected by the six TBC proteins. These data demonstrate that TBC proteins are important regulators in GPCR trafficking. As the molecular mechanisms underlying the anterograde cell surface transport of GPCRs are poorly understood, these studies provide important insights into regulatory mechanisms of GPCR forward trafficking.

Our studies have also provided direct evidence implicating that TBC1D20, TBC1D22A, and RN-tre regulate the transport of GPCRs from the ER to the Golgi (Figure 7G). This became evident as enhanced expression of TBC1D20, TBC1D22A, and RN-tre induced extensive ER localization of all GPCRs studied, as well as the Golgi-localized α_2B_-AR YS mutant. This is also strongly supported by fact that TBC1D20, TBC1D22A, and RN-tre significantly enhanced the formation of simple glycosylation that occurs in the ER. In contrast, TBC1D5, TBC1D6, and TBC1D8B are more likely involved in the transport of GPCRs at the level of the Golgi (Figure 7G), as their expression caused GPCR accumulation in the Golgi without disrupting the ER exit and N-linked glycosylation of the receptors. These data clearly demonstrate that distinct TBC proteins regulate the cell surface transport of GPCRs at discrete steps (Figure 7G).

Another important finding is that we have identified TBC1D6 as a specific GAP for Rab26. First, TBC1D6, but not its catalytically inactive RQ-AA mutant and other TBC proteins involved in GPCR transport, markedly inhibited the Golgi localization of Rab26 in cell, indicative of Rab26 inactivation. Second, TBC1D6 also markedly inhibited the activation-dependent interaction of Rab26 with the receptor. These data imply a possible molecular mechanism underlying the function of Rab26 and TBC1D6 in regulating GPCR cell surface trafficking, in which TBC1D6 inactivates Rab26 and inhibits Rab26 interaction with GPCRs and subsequent formation of transport machinery (Figure 7G). Third, the direct evidence indicating that TBC1D6 is a GAP for Rab26 is that it significantly enhanced the GTP hydrolysis by Rab26. Fourth, our previous and current studies reveal that both TBC1D6 and Rab26 regulate the post-Golgi transport of GPCRs, but not other plasma membrane proteins (such as VSVG and EGFR). Fifth, TBC1D6 and Rab26 interact and the interaction is independent of their activation statuses. Interestingly, both the TBC domain and the C terminus are able to interact with Rab26 albeit with different binding affinities and the C-terminal interaction with Rab26 enhances the GAP activity of TBC1D6. Sixth, it is worthwhile to point out that the same effects produced by overexpression and depletion of TBC1D6 suggest that proper GDP/GTP cycling of its target Rabs is crucial for the promotion of membrane trafficking of GPCRs. Indeed, both Rab26 GTP- and GDP-bound mutants prevent GPCRs to transport to the cell surface. Nevertheless, these data strongly suggest that Rab26 and TBC1D6 are a pair of Rab and GAP and provide an important mechanism underlying their function in regulating the post-Golgi traffic of GPCRs (Figure 7G).

The functions of TBC proteins in regulating GPCR transport are most likely specific. The most important evidence to support this is that catalytically inactive mutants of the six TBC proteins completely reversed their inhibitory effects on the cell surface transport of α_2B_-AR, indicating that their effects on GPCR trafficking were caused by catalytic inactivation of Rab GTPases. Indeed, previous studies have shown that TBC1D20 is a GAP for Rab1 and Rab2 (Haas et al., 2007; Ishida et al., 2012; Nachmias et al., 2012; Nevo-Yassaf et al., 2012; Sklan et al., 2007) and RN-tre is a GAP for Rab43 (Fuchs et al., 2007; Haas et al., 2005, 2007). All three Rabs modulate the transport of multiple GPCRs between the ER and the Golgi (Dong and Wu, 2007; Li et al., 2017; Wu et al., 2003). We have shown here that TBC1D6 is a GAP for Rab26 that we have previously demonstrated to regulate the post-Golgi transport of GPCRs as discussed above (Li et al., 2012). Furthermore, the TBC domain of TBC1D6 is able to produce the maximal inhibitory effect on GPCR cell surface export. It should be pointed out that Rab7 (Borg Distefano et al., 2018; Seaman et al., 2009) and Rab33 (Fuchs et al., 2007; Pan et al., 2006) were suggested to be target Rabs for TBC1D5 and TBC1D22, but they unlikely regulate the forward cell surface transport of GPCRs as demonstrated in our previous (Li et al., 2017) and current studies, suggesting that the function of TBD1D5 and TBC1D22A in GPCR trafficking may not be mediated via the inactivation of these Rabs. This notion is also supported by the fact that the expression of TBC1D25 and RUTBC1, other two well-characterized Rab33 GAPs (Itoh et al., 2011; Nottingham et al., 2011), did not affect the cell surface transport of α_2B_-AR in our initial screening assay (Figure 1). In addition, TBC1D20 was the only TBC protein to inhibit the cell surface transport of EGFR and VSVG, consistent with a previous report showing that TBC1D20 blocks VSVG export from the ER (Haas et al., 2007). Altogether, these data strongly suggest the specific regulatory function of TBC proteins in GPCR forward trafficking. However, we cannot completely exclude the possibility that the inhibitory effect of TBC1D22A on GPCR transport was caused by the disruption of the Golgi structure as suggested in a previous study for Shiga toxin uptake (Fuchs et al., 2007). Nevertheless, these data suggest that specific Rabs and TBC proteins may form pairs to coordinate the ER-to-Golgi (e.g., Rab1-TBC1D20 and Rab43-RN-tre) and the Golgi-to-cell surface transport (e.g., Rab26-TBC1D6) of GPCRs (Figure 7G). Future studies to identify Rab substrates of TBC1D5, TBC1D8B, and TBC1D22A and evaluate their effect on the cell surface transport of GPCRs will further demonstrate specific function of Rab-TBC pairs in GPCR anterograde trafficking.

As the most structurally diverse superfamily of membrane proteins, GPCR trafficking, particularly their targeting to the cell surface, has been well described to play a crucial role in regulating the functionality of the receptors and its dysregulation directly causes many human diseases (Conn et al., 2007; Morello and Bichet, 2001). It is also interesting to note that recent studies have shown that TBC proteins may be associated with a variety of human diseases (Liegel et al., 2013; Vizoso et al., 2015), but the exact mechanisms remain largely unknown. Emerging evidence from the past decade studies suggest that the cell surface transport of GPCRs is tightly controlled by many factors, including specific export motifs embedded within the receptors (Dong et al., 2012; Dong and Wu, 2006; Duvernay et al., 2009; Zhang et al., 2011), receptor activity modifying proteins, chaperones, escort proteins, gatekeepers, protein kinases, and small GTPases (Chen et al., 2009; Colley et al., 1991; Doly et al., 2016; Dwyer etal., 1998; Ferreira et al., 1996; Hay and Pioszak, 2016; McLatchie et al., 1998; Sauvageau et al., 2014; Shiwarski et al., 2017; Tai et al., 1999). As shown in the current study, GPCR trafficking is tightly controlled by specific TBC proteins. Therefore, to thoroughly elucidate regulatory mechanisms of GPCR export to the functional destinations may provide an important foundation for the development of new therapeutic strategies by targeting GPCR export trafficking.

In summary, our present study has identified multiple TBC proteins as crucial regulators for GPCR trafficking, specifically anterograde cell surface transport, *en route* from the ER through the Golgi. These functions of TBC proteins are mediated through their GAP activity to inactivate target Rab GTPases and inhibit Rab interaction with the receptors. Overall, our data presented in this paper demonstrate the functional importance of the TBC family proteins in GPCR trafficking and provide important insights into regulation of GPCR targeting to the functional destination, as well as regulation of general vesicle-mediated membrane trafficking, by the TBC family proteins as Rab-specific GAPs.

## STAR★METHODS

### LEAD CONTACT AND MATERIALS AVAILABILITY

Further information and requests for resources and reagents should be directed to and will be fulfilled by the Lead Contact, Guangyu Wu (guwu@augusta.edu).

### EXPERIMENTAL MODELS AND SUBJECT DETAILS

#### Cell culture

HEK293 cells, HT-29 cells which express endogenous α_2A_-AR, and MCF-7 cells which express endogenous α_2B_-AR were cultured AT 37°C HT-29 human colon cancer cells expressing endogenously α_2A_-AR were cultured in Dulbecco’s modified Eagle’s medium (DMEM) with 10% fetal bovine serum, 100 units/ml penicillin and 100 μg/ml streptomycin.

### METHOD DETAILS

#### Plasmid constructions

GPCRs tagged with GFP at their C-termini or HA at their N-termini were generated as described previously (Dong et al., 2010,^2012^; Li et al., 2012). TBC proteins and Rab3GAP in the pEGFP-C1 vector were generated as described (Ishibashi et al., 2009; Itoh et al., 2011). TBC proteins were also cloned into the pDsRed-monomer-C1 vector at the BamH1 and XhoI (TBC1D5), EcoR1 and XhoI (TBC1D20), or EcoRI and BamHI (TBC1D6, TBC1D8B, TBC1D22A and RN-tre) restriction sites using primers (Table S1). The GST-Rab26 and GST-TBC1D6 constructs were generated in the pGEX-4T-1 vector using the primers (Table S1). TBC and Rab26 mutants were generated by using QuikChange site-directed mutagenesis. All constructs used in the present study were verified by nucleotide sequence analysis.

#### Transient transfection

Transient transfection of cells were carried out by using Lipofectamine 2000 as described previously (Wu et al., 2003). Transfection efficiency of the cells was estimated to be greater than 75% based on microscopy detecting the fluorescence of tagged proteins.

#### Generation of stable and inducible cell lines expressing α_2A_-AR and α_2B_-AR

HEK293 cell lines stably expressing HA-α_2B_-AR were generated as described previously (Li et al., 2012). Stable HEK293 cell lines inducibly expressing HA-α_2A_-AR and HA-α_2B_-AR was generated using the Tet-On 3G Tetracycline Inducible Gene Expression System (Clontech Laboratories, Inc.) as described previously (Zhang et al., 2016a). The cell lines expressing 8.3 × 10^5^ α_2A_-AR and 8.5 × 10^5^ α_2B_-AR per cell were utilized in the current study.

#### Depletion of TBC1D6 by siRNA

Two Stealth RNAi duplexes (siRNA) targeting to human TBC1D6 (NCBI:AB449887; # 1,5′-CCUGUACAAUGUGCUGCUGGCAUAU-3′; # 2, 5′-UCGUGAUGGAGUGUCACACGUUUAU-3′) and a negative control med GC duplex were purchased from Invitrogen. siRNAs targeting to Rab26 and GGA3 were described (Li et al., 2012; Zhang et al., 2016a). siRNA-mediated knockdown of TBC1D6, Rab26 and GGA3 was carried out as described previously (Wu et al., 2003). All of the experiments were performed at 72 h after the first siRNA transfection.

#### Radioligand binding of intact live cells

The cell surface expression of α_2_-AR and β_2_-AR was measured by ligand binding of intact live cells as described previously (Dong et al., 2012; Li et al., 2012) using [^3^H]-RX821002 and [^3^H]-CGP12177, respectively. For measurement of endogenous α_2_-AR, HT-29 and MCF-7 cells were cultured on 6-well dishes and transfected with TBC1D6 constructs for 36-48 h or siRNA targeting TBC1D6 for 72 h. All radioligand binding assays were performed in triplicate.

#### Flow cytometry

For measurement of receptor expression at the cell surface, HEK293 cells transfected with HA-tagged receptors were suspended in PBS containing 1% fetal bovine serum and incubated with high affinity anti-HA-fluorescein (3F10) at 2 μg/ml for 30 min at 4°C. For measurement of total receptor expression, HEK293 cells were permeabilized with 0.2% Triton X-100 in PBS for 5 min before incubation with anti-HA antibodies. The fluorescence was analyzed on a flow cytometer (Dickinson FACSCalibur) as described (Wu et al., 2003).

#### Fluorescence microscopy

Cells were fixed with 4% paraformaldehyde for 15 min, permeabilized with 0.25% Triton X-100 for 5 min, and blocked with normal donkey serum for 30 min. The cells were sequentially stained with primary antibodies (1:200 dilution for GM130 and 1:50 dilution for calregulin) and Alexa fluor-conjugated secondary antibodies (1:500 dilution). Images were captured using a Zeiss LSM780 confocal microscope equipped with a 63 × objective.

#### Deglycosylation

Deglycosylation of GPCRs by treatments with Endo H and PNGase F enzymes was performed following the manufacturer’s instructions with modifications. Briefly, cell lysates prepared from HEK293 cells transfected with individual receptors with or without TBC proteins were incubated in denaturing buffer containing 0.5% SDS and 40 mM DTT for 30 min at 37°C and the denatured proteins were digested with 1-2 μL enzymes in GlycoBuffer for 1 h at 37°C.

#### Measurement of VSVG transport from the ER through the Golgi to the plasma membrane

The transport of VSVG was measured by using its temperature sensitive mutant (VSVGtsO45) which was misfolded and retained within the ER at the restrictive temperature 40°C and correctly delivered to the Golgi and plasma membrane at the permissive temperature 32°C (Presley et al., 1997). HEK293 cells grown on coverslips in 6-well dishes were transfected with 0.25 μg of VSVGtsO45-GFP constructs together with dsRed-tagged TBC proteins. The cells were cultured for 24 h at 40°C to induce the accumulation of VSVG in the ER and then transferred to 32°C for up to 180 min to allow VSVG to transport to the Golgi and the cell surface. After the cells were fixed VSVG expression at the Golgi and the plasma membrane were quantified by fluorescent intensity using the ImageJ software. A total of 25-30 cells were quantified in three experiments.

#### Measurement of ERK1/2 activation

HEK293 cells stably expressing α_2B_-AR were cultured on 6-well dishes and transfected with TBC1D6 or siRNA targeting TBC1D6. The cells were starved for 3 h before stimulation with UK14304 at 1 μM for 5 min. Stimulation was terminated by the addition of 200 μL of ice-cold cell lysis buffer. ERK1/2 activation was determined by measuring ERK1/2 phosphorylation by immunoblotting.

#### GST fusion protein pulldown assays

GST fusion protein pulldown assays were carried out by using the MagneGST pulldown system as described previously (Li et al., 2012; Zhang et al., 2016a). Briefly, GST fusion proteins were incubated with total HEK293 cell homogenates in 500 μL of binding buffer containing 20 mM Tris-HCl, pH 7.5, 140 m NaCl, 1% Nonidet P-40 for 4 h at 4°C. The bound proteins were solubilized and detected by immunoblotting.

#### *In vitro* GAP assays

The GAP assays were performed as described previously (Fuchs et al., 2007; Haas et al., 2005) with modifications. Briefly, GST-Rab26 or GST-Rab1 (50 pmol) were incubated with 1 μl of [γ-^32^P]GTP in 50 μl buffer (50 mM HEPES-NaOH, pH 6.8, 1 mM DTT, 1 mg/ml bovine serum albumin, 1 mM EDTA and 0.5 mM of an equal mixture of GTP and MgCl_2_) for 15 min at 30°C. GST-TBC1D6 or its mutants (4.6 pmol) were then added to the reaction mixture and incubated at 30°C for 60 min. At each time point, 5 μl of the reaction was removed and mixed with 795 μl of activated charcoal. After centrifugation, 400 μl of the cleared supernatant was counted and the ^32^Pi release was determined by the radioactivity difference at different time points.

#### BRET assays

The live cell-based BRET assay to measure the possible interaction between α_2B_-AR and Rab26 was carried out as described previously (Li et al., 2012, 2017). HEK293 cells were cultured on 6-well dishes and transfected with 0.1 μg of Rab-Rluc8 and 1.5 μg of α_2B_-AR-Venus plus 1.0 μg of the pCMV-myc vector or myc-TBC for 36 h.

### STATISTICAL ANALYSIS

All data were calculated and presented as mean ± SE. The exact value of n is given in each figure legend, where n represents the number of independent repeats. Statistical analysis was performed using unpaired Student’s t test. p < 0.05 was considered as statistically significant.

## Supplementary Material

1

2

## Figures and Tables

**Table:** KEY RESOURCES TABLE

REAGENT or RESOURCE	SOURCE	IDENTIFIER
Antibodies		
Mouse monoclonal anti-GFP (clone B-2)	Santa Cruz Biotechnology	sc-9996
Rabbit polyclonal anti-calregulin (clone H-170)	Santa Cruz Biotechnology	sc-11398
Mouse monoclonal anti-β-actin (clone C4)	Santa Cruz Biotechnology	sc-47778
Mouse monoclonal anti-p-ERK (clone E-4)	Santa Cruz Biotechnology	sc-7383
Mouse monoclonal anti-GM130 (clone 35)	BD Biosciences	610823
Rabbit polyclonal anti-p44/42 MAPK (Erk1/2)	Cell Signaling Technology	9102
Mouse monoclonal anti-HA (clone 12CA5)	Roche	11583816001
Rat monoclonal anti-HA-fluorescein (clone 3F10)	Roche	11988506001
Goat anti-mouse IgG (H+L), Alexa Fluor 647	Thermo Fisher Scientific	A-21235
Goat anti-rabbit IgG (H+L), Alexa Fluor 647	Thermo Fisher Scientific	A-21244
Chemicals, Peptides, and Recombinant Proteins		
UK14304	Sigma-Aldrich	U104
NSC12155	Sigma-Aldrich	S6951
Activated charcoal	Sigma-Aldrich	242276
IPTG	Sigma-Aldrich	I5502
Doxycycline hyclate	Sigma-Aldrich	D9891
[^3^H]-RX821002	Perkin-Elmer	NET1153250UC
[^3^H]-CGP12177	Perkin-Elmer	NET1061250UC
[γ-^32^P]-GTP	Perkin-Elmer	BLU004Z250UC
Lipofectamine 2000	Thermo Fisher Scientific	11668019
GTP	Thermo Fisher Scientific	18332015
EGF	Thermo Fisher Scientific	PHG0311
MagneGST glutathione particles	Promega	V8611
Dulbecco’s modified eagles medium	GE Healthcare Life Sciences	SH30243.01HI
Fetal bovine serum	GE Healthcare Life Sciences	SH30396.03HI
Endo H	New England Biolabs	P0702L
PNGase F	New England Biolabs	P0708S
Experimental Models: Cell Lines		
HEK293	ATCC	CRL-1573
HT-29	ATCC	HTB-38
MCF-7	ATCC	HTB-22
Oligonucleotides		
Human TBC1D6 Stealth siRNA #1	Thermo Fisher Scientific	1299001 ID:HSS128833
Human TBC1D6 Stealth siRNA #2	Thermo Fisher Scientific	1299001 ID:HSS128835
Stealth siRNA negative control, Med GC	Thermo Fisher Scientific	12935300
Primers (Table S1)	This paper	N/A
Recombinant DNA		
α_2A_-AR-GFP	Li et al., 2017	N/A
HA-α_2A_-AR	Li et al., 2017	N/A
α_2B_-AR YS-GFP	Dong and Wu, 2006	N/A
α_2B_-AR-GFP	Wu et al., 2003	N/A
HA-α_2B_-AR	Li et al., 2017	N/A
α_2B_-AR-Venus	Li et al., 2012	N/A
AT1R-GFP	Li et al., 2017	N/A
β_2_-AR-GFP	Wu et al., 2003	N/A
GFP-TBC	Itoh et al., 2011	N/A
DsRed-TBC	This paper	N/A
GFP-Rab3GAP	Itoh et al., 2011	N/A
EGFR-GFP	Li et al., 2017	N/A
VSVGtsO45-GFP	Addgene	11912
GST-Rab1	This paper	N/A
GST-Rab26	This paper	N/A
GST-TBC1D6	This paper	N/A
GST-α_2B_-AR ICL3	Wu et al., 2003	N/A
GST-α_2A_-AR ICL3	Zhang et al., 2016a	N/A
GST-AT1R CT	Duvernay et al., 2011	N/A
Rab26-Rluc8	Li et al., 2017	N/A
Software and Algorithms		
ImageJ	NIH	https://imagej.nih.gov/ij/

## Highlights

- Six TBC proteins activity-dependently regulate the cell surface export of GPCRs
- TBC proteins selectively control ER-Golgi or Golgi-cell surface transport of GPCRs
- TBC1D6 is a GAP for Rab26 and physically associates with Rab26
- TBC1D6 modulates Rab26 interaction with and the post-Golgi traffic of GPCRs
