# Supplementary material for: Specific TBC Domain-Containing Proteins Control the ER-Golgi-Plasma Membrane Trafficking of GPCRs
Source: Cell Rep. Author manuscript; Available in PMC 2019 Jul 18. (PMC6639060; doi:10.1016/j.celrep.2019.05.033)
Supplement: 1 [file NIHMS1534188-supplement-1.pdf]

Cell Reports, Volume *28*

Supplemental Information

Specific TBC Domain-Containing Proteins Control the ER-Golgi-Plasma Membrane Trafficking of GPCRs

Zhe Wei, Maoxiang Zhang, Chunman Li, Wei Huang, Yi Fan, Jianhui Guo, Mostafa Khater, Mitsunori Fukuda, Zheng Dong, Gang Hu, and Guangyu Wu

Figure S1

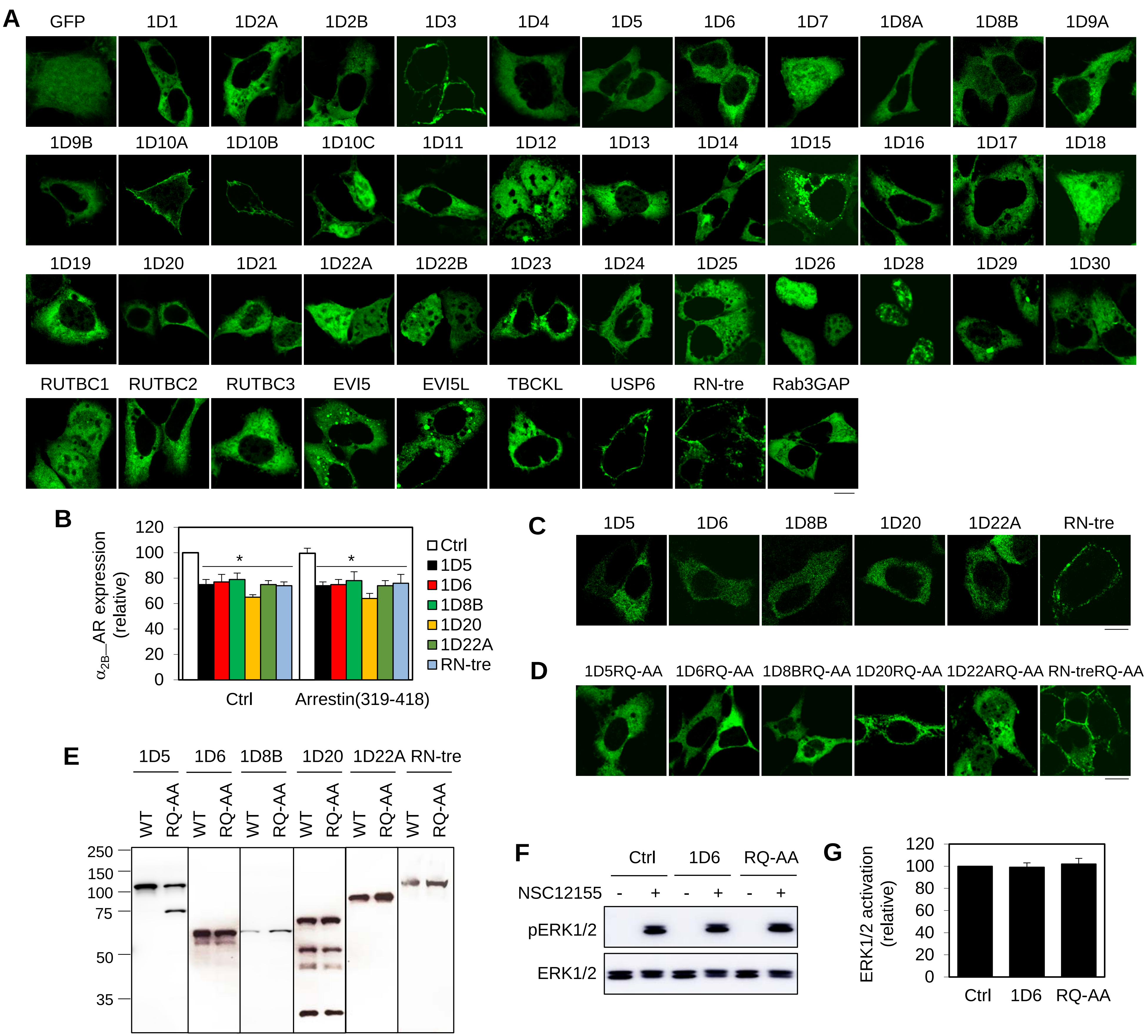

A
GFP
1D1
1D2A
1D2B
1D3
1D4
1D5
1D6
1D7
1D8A
1D8B
1D9A
1D9B
1D10A
1D10B
1D10C
1D11
1D12
1D13
1D14
1D15
1D16
1D17
1D18
1D19
1D20
1D21
1D22A
1D22B
1D23
1D24
1D25
1D26
1D28
1D29
1D30
RUTBC1
RUTBC2
RUTBC3
EVI5
EVI5L
TBCKL
USP6
RN-tre
Rab3GAP
B
α2B AR expression (relative)
120
100
80
60
40
20
0
*
Ctrl
Arrestin(319-418)
Ctrl
1D5
1D6
1D8B
1D20
1D22A
RN-tre
C
1D5
1D6
1D8B
1D20
1D22A
RN-tre
D
1D5RQ-AA
1D6RQ-AA
1D8BRQ-AA
1D20RQ-AA
1D22ARQ-AA
RN-treRQ-AA
E
1D5
1D6
1D8B
1D20
1D22A
RN-tre
WT
RQ-AA
250
150
100
75
50
35
F
Ctrl
1D6
RQ-AA
NSC12155
-
+
pERK1/2
ERK1/2
G
ERK1/2 activation (relative)
120
100
80
60
40
20
0
Ctrl
1D6
RQ-AA

Figure S1. Screening for TBC proteins involved in the surface transport of α_{2B}-AR. Related to Figure 1.

(A) Expression and subcellular localization of TBC proteins. Individual GFP-tagged TBC proteins were transiently expressed in HEK293 cells and their subcellular localization was revealed by confocal microscopy.

(B) Effect of dominant negative arrestin-3 mutant on the cell surface expression of α_{2B}-AR. HEK293 cells were transfected with α_{2B}-AR and individual TBC proteins together with control vector or dominant negative arrestin-3(201-490) for 36 h and then the cell surface α_{2B}-AR expression was determined by intact cell ligand binding

(C) Expression of TBC proteins in MCF7 cells.

(D) Expression of TBC mutants in HEK239 cells.

(E) Western blots showing the expression of TBC proteins and their RQ-AA mutants.

(F) Effect of TBC1D6 on the activation of ERK1/2 by NSC12155. HEK293 cells were transfected with the pEGFP-C1 vector (Ctrl), GFP-TBC1D6 or GFP-TBC1D6 RQ-AA mutant and then stimulated with NSC12155 at a concentration of 10 μM for 5 min. The activation of ERK1/2 was measured by immunoblotting.

(G) Quantitative data shown in F

The data shown in B and G are mean ± SE (n=3). *$p<0.05$ versus Ctrl. The images are representative of at least 3 experiments. Scale bars, 10 μm.

Figure S2

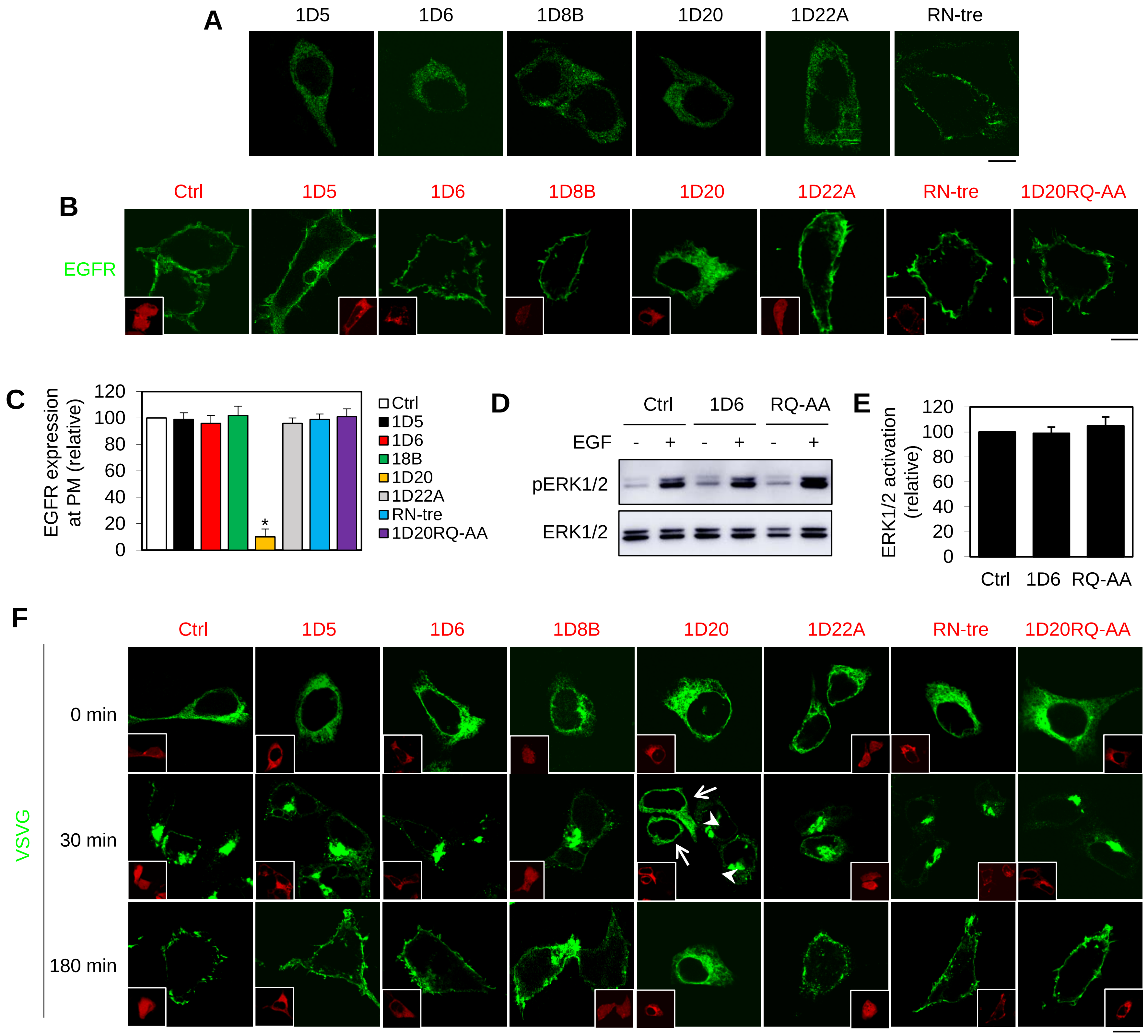
A
1D5
1D6
1D8B
1D20
1D22A
RN-tre
B
Ctrl
1D5
1D6
1D8B
1D20
1D22A
RN-tre
1D20RQ-AA
EGFR
C
EGFR expression at PM (relative)
120
100
80
60
40
20
0
*
Ctrl
1D5
1D6
18B
1D20
1D22A
RN-tre
1D20RQ-AA
D
Ctrl
1D6
RQ-AA
EGF
- + - + - +
pERK1/2
ERK1/2
E
ERK1/2 activation (relative)
120
100
80
60
40
20
0
Ctrl
1D6
RQ-AA
F
Ctrl
1D5
1D6
1D8B
1D20
1D22A
RN-tre
1D20RQ-AA
VSVG
0 min
30 min
180 min

Figure S2. Expression of TBC proteins and their effects on the cell surface transport of EGFR and VSVG. Related to Figure 3.

(A) Expression of TBC proteins in HT29 cells. The images are representatives of at least 3 experiments.

(B) Effect of TBC proteins on the cell surface expression of EGFR.

(C) Quantitative data shown in B. The cell surface expression of EGFR was determined by measuring the fluorescent intensity at the cell surface. The data shown are relative to the total expression of EGFR with a total of 25-30 cells quantified.

(D) Effect of TBC1D6 on the activation of ERK1/2 by EGF. HEK293 cells were transfected with the pEGF-C1 vector, GFP-TB1D6 or GFP-TBC1D6 RQ-AA mutant and then stimulated with EGF at 10 mg/ml for 10 min. ERK1/2 activation was determined by immunoblotting.

(E) Quantitative data shown in D.

(F) Effect of TBC proteins on the ER-Golgi-plasma membrane transport of VSVG. HEK293 cells were transfected with VSVGtsO45-GFP together with dsRed-C1 (Ctrl) or individual dsRed-TBC proteins. The cells were cultured at 40˚C for 24 h (0 min) and then shifted to 32˚C for different periods of time. The images shown are representative VSVG expression at 0, 30 and 180 min. Arrows indicate VSVG accumulation in TBC1D20-expressing cells and arrow heads indicate VSVG transport to the Golgi in cells without TBC1D20 transfection.

The data shown in C and E are mean ± SE (n=3). *$p < 0.05$ versus Ctrl. Scale bars, 10 μm.

Figure S3

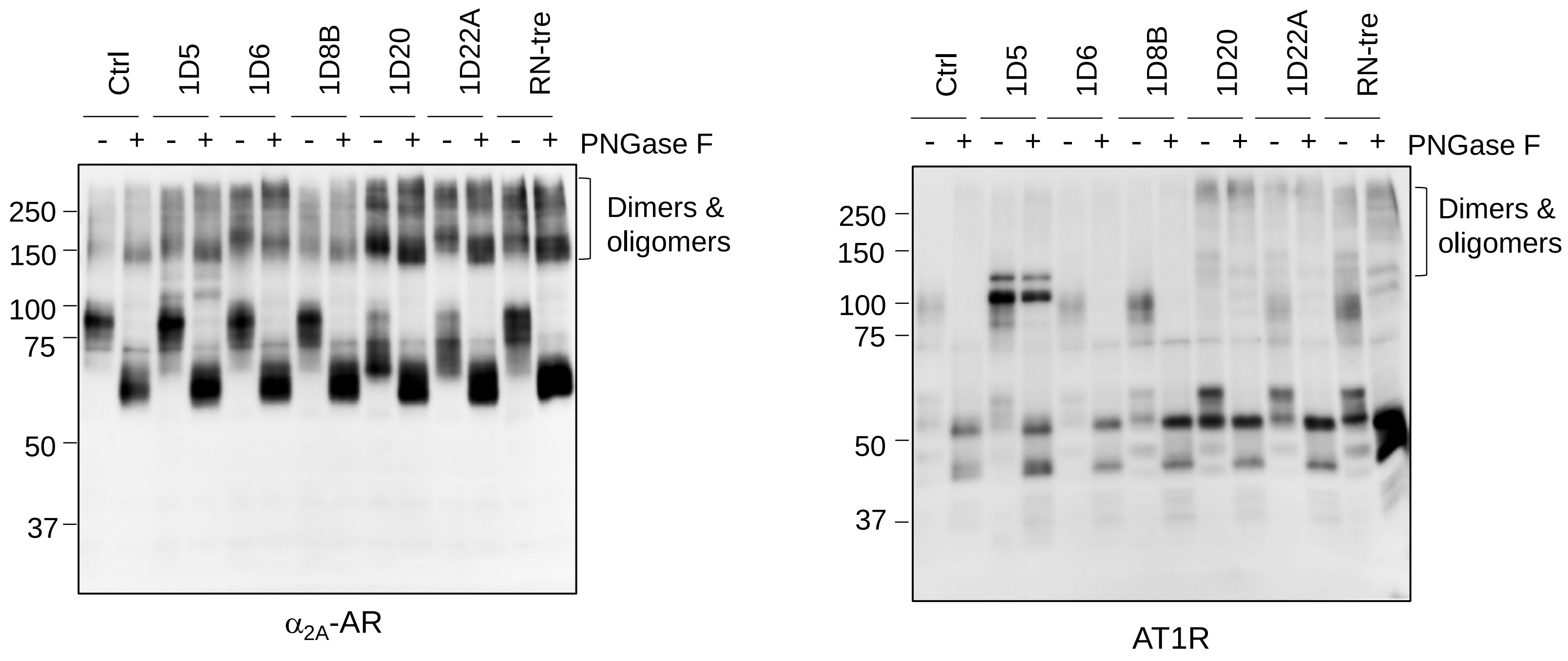

Figure S3. Effect of TBC proteins on the glycosylation of α_{2A}-AR (left panel) and AT1R (right panel). Related to Figure 4.
HEK293 cells were transfected with the receptors with or without TBC proteins. Total cell lysates were treated with PNGase F for 1 h at 37°C. Upper bands represent most likely dimers and oligomers of the receptors.

Figure S4

A

α_{2B}-AR ICL3 205 KRSHCRGLGA KRGSGEGESK KPQPVAGGVP TSAKVPTLVS PLSSVGEANG HPKPPREKEE
GETPEDPEAR ALPPTWSALP RSGQGQKKGT SGATAEEGDEEDEEEVEECE PQTLPASPAS
VCNPPLQQPQ TSRVLATLRG QVLLGKNVGV ASGQWWRRRT QLSRE 369

α_{2A}-AR ICL3 239 KRRTRVPPSR RGPDAVAAPP GGTERRPNGL GPERSAGPGG AEAEPLPTQL NGAPGEPAPA
GPRDTDALDL EESSSSDHAE RPPGPRRPER GPRGKGKARA SQVKPGDSLP RRGPGATGIG
TPAAGPGEER VGAAKASRWR GRQNRE 384

AT1R CT 303 GFLGKKFKKY FLQLLKYIPP KAKSHSSLST KMSTLSYRPS DNMSSSAKKP ASCFEVE 359

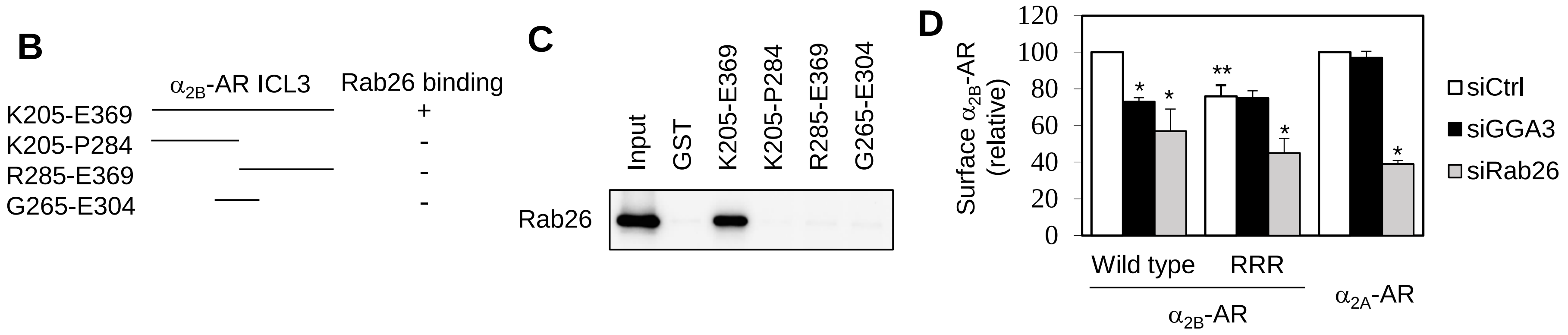

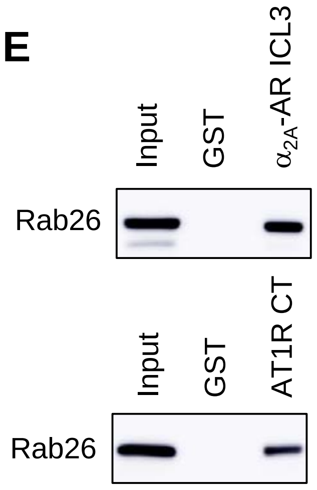

F

GPCRs		Rab26	GGA3
α_{2B}-AR	Interaction	Yes	Yes
	Interaction domain	ICL3	ICL3
	Interaction motif	No	RRR
	Cell surface transport	Yes	Yes
	Cell surface transport of RRR mutant	Yes	No
α_{2A}-AR	Interaction	Yes	No
	Interaction domain	ICL3	No
	Interaction motif	Unknown	No
	Cell surface transport	Yes	No
AT1R	Interaction	Yes	Unknown
	Interaction domain	C-terminus	Unknown
	Cell surface transport	Yes	Unknown

Figure S4. Regulation of α_{2B}-AR cell surface transport by Rab26 and GGA3 and Rab26 interaction with AT1R and α_{2A}-AR. Related to Figure 5.

(A) Sequences of the third intracellular loop (ICL3) of α_{2A}-AR and α_{2B}-AR and the C-terminus (CT) of AT1R. The GGA3-binding RRR motif is red colored.

(B) Summary of progressive deletion to identify the possible Rab26-binding domain in the ICL3 of α_{2B}-AR as shown in C.

(C) A representative blot showing the interaction of Rab26 with different ICL3 fragments. Similar results were obtained in at the least three experiments.

(D) Effect of siRNA-mediated depletion of GGA3 and Rab26 on the cell surface transport of α_{2B}-AR, the α_{2B}-AR RRR mutant in which the RRR motif was mutated to AAA, and α_{2A}-AR. The cell surface expression of the receptors was measured by intact cell ligand binding. The data shown are percentages of wild type receptor expression at the cell surface and presented as mean ± SE (n=3). *$p < 0.05$ versus respective siCtrl and **$p < 0.05$ versus wild type.

(E) The AT1R CT (upper panel) and the α_{2A}-AR ICL3 (lower panel) were generated as GST fusion proteins and their interaction with Rab26 was measured by GST fusion protein pulldown assays. Similar results were obtained in at least three separate experiments.

(F) Summary of differential regulation of GPCR transport by Rab26 and GGA3.

Figure S5

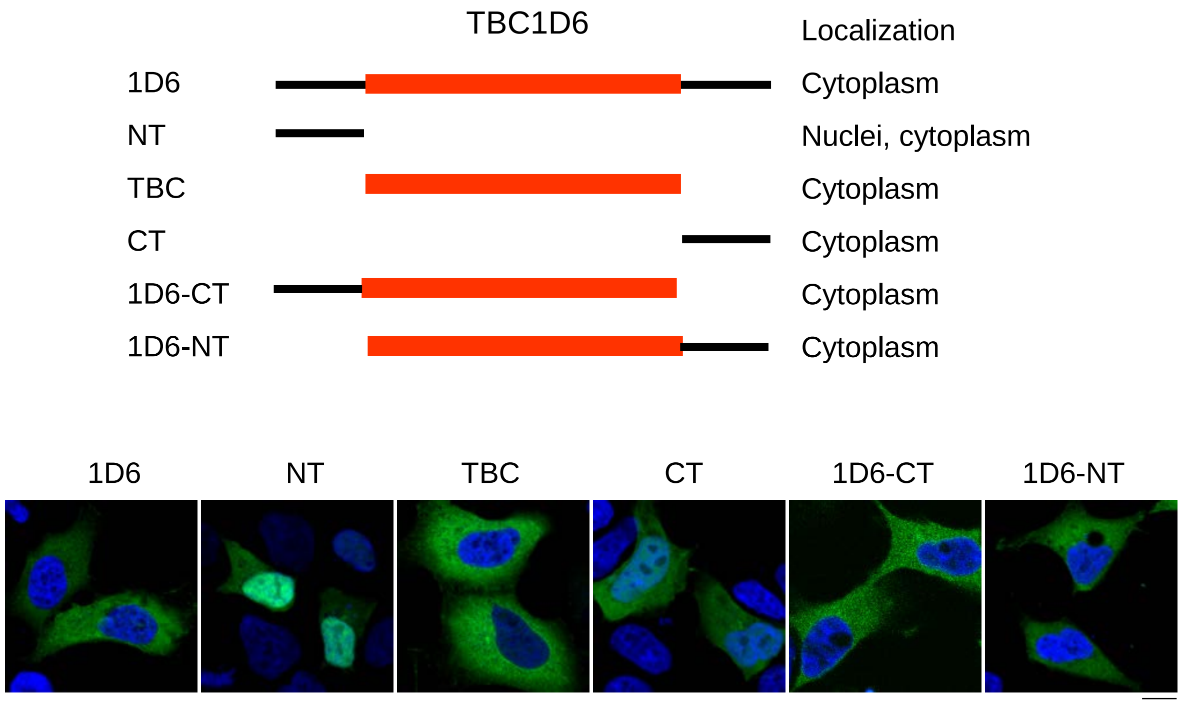

Figure S5. Expression and subcellular localization of TBC1D6 and its domains. Related to Figure 6.
TBC1D6 and its domains were tagged with GFP at their N-termini and transiently expressed in HEK293 cells. Their expression and subcellular distribution were visualized by confocal microscopy. Scale bar, 10 µm.

Figure S6

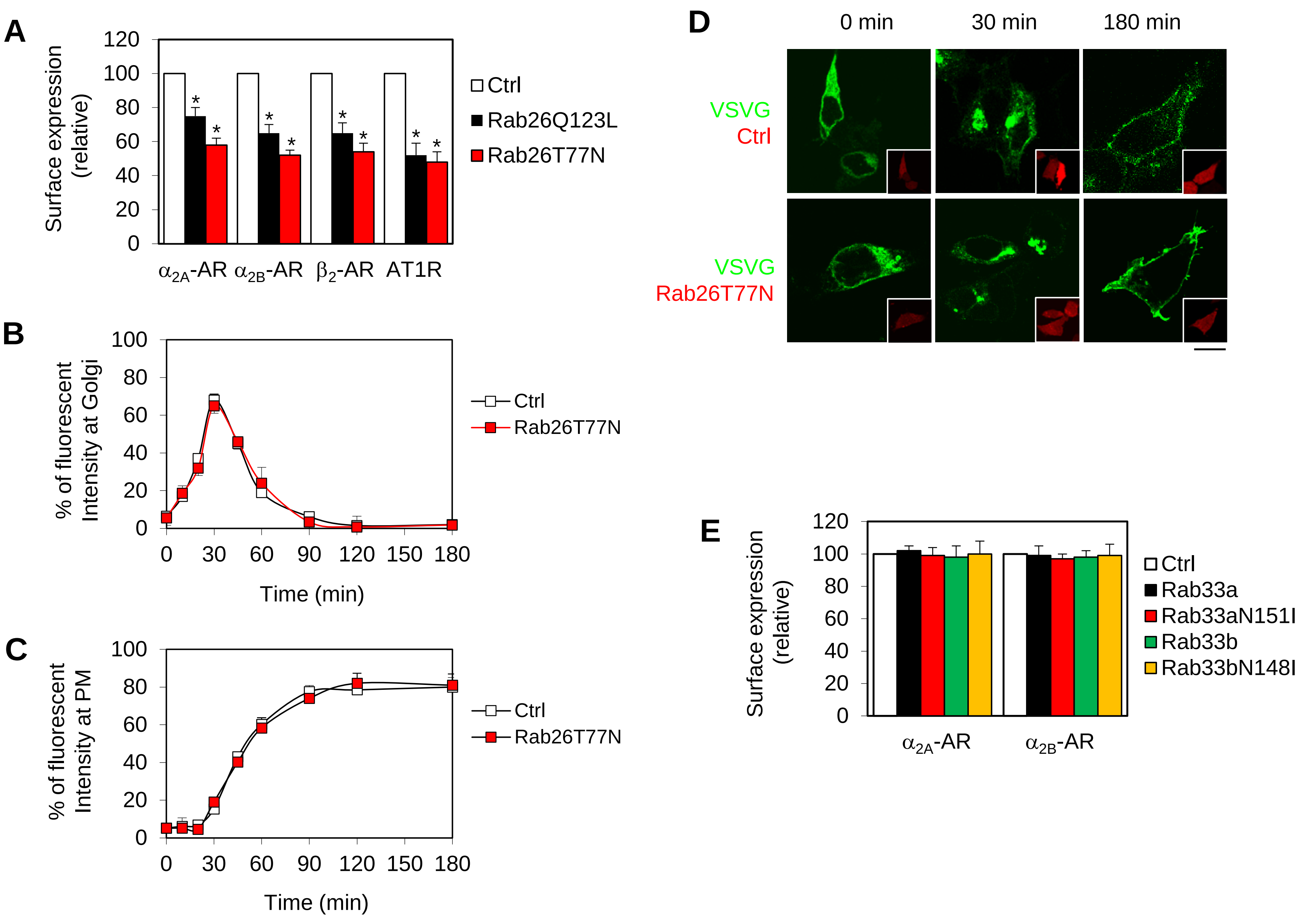

Figure S6. Effect of Rab26 and Rab33 mutants on the cell surface transport of GPCRs and VSVG. Related to Figure 7.

(A) Effect of Rab26 mutants on the cell surface expression of a group of GPCRs. HEK293 cells were transfected with individual receptors together with Rab26 mutants. The cell surface expression of the receptors was measured by intact cell ligand binding or flow cytometry.

(B) Effect of Rab26T77N on the expression of VSVG at the Golgi. HEK293 cells were transfected with VSVGtsO45-GFP together with dsRed-C1 (Ctrl) or dsRed-Rab26T77N. The cells were cultured at 40˚C for 24 h (0 min) and then shifted to 32˚C for up to 180 min. The expression of VSVG at the Golgi was measured by fluorescent intensity.

(C) Effect of Rab26T77N on the expression of VSVG at the plasma membrane (PM) as described in B.

(D) Representative images showing VSVG expression of at the Golgi after 30 min and at the plasma membrane after 180 min.

(E) Effect of Rab33 on the cell surface transport of GPCRs. HEK293 cells were transfected with α_{2A}-AR or α_{2A}-AR together with Rab33a, Rab33b or their mutants and the cell surface expression of the receptors were measured by intact cell ligand binding.

The data are presented as mean ± SE (n=3-4). *$p < 0.05$ versus Ctrl. Scale bar, 10 μm.

Table S1. Primers used in the manuscript. Related to Figures 1, 2 and 6.

TBC mutants	Sense (S) or antisence (A)	Sequences
TBC1D5 R-A	S	GAACAAGATGTCAAAGCAACGTTTCCTGAAATGC
	A	GCATTTCAGGAAACGTTGCTTTGACATCTTGTTC
TBC1D5 Q-A	S	CGAGCAGTTGCTTTATAAAGCGGGCATGCACGAACTGTTAG
	A	CTAACAGTTCGTGCATGCCCGCTTTATAAAGCAACTGCTCG
TBC1D6 R-A	S	ATCAGGACAGACCTGAACGCAACCTTCCCCGACAACGTG
	A	CACGTTGTCGGGGAAGGTTGCGTTCAGGTCTGTCCTGAT
TBC1D6 Q-A	S	CCAGGGAGTGGGCTACTGCGCGGGAATGAATTTTATAGCAG
	A	CTGCTATAAAATTCATTCCCGCGCAGTAGCCCACTCCCTGG
TBC1D8B R-A	S	GAAATTGAACGTGATTTACGTGCATCTCTGCCTGAGCACCCAGCC
	A	GGCTGGGTGCTCAGGCAGAGATGCACGTAAATCACGTTCAATTTC
TBC1D6B Q-A	S	CCAAAATTGGATACTGCGCGGCAATGAATATTTTGAC
	A	GTCAAAATATTCATTGCCGCGCAGTATCCAATTTTGG
TBC1D20 R-A	S	CAAGTGTTGCTGGACGTCCGGGCGTCATTGCGGCGGTTCCCTCCTG
	A	CAGGAGGGAACCGCCGCAATGACGCCCGGACGTCCAGCAACACTTG
TBC1D20 Q-A	S	CCTCAGCTGCACTACTACGCGGGCTACCATGACATTGTG
	A	CACAATGTCATGGTAGCCCGCGTAGTAGTGCAGCTGAGG
TBC1D22A R-A	S	CAGATCCACATAGACATCCCTGCCATGAGCCCTGAAGCGTTGATC
	A	GATCAACGCTTCAGGGCTCATGGCAGGGATGTCTATGTGGATCTG
TBC1D22A Q-A	S	CCAGCCAGTGGATACGTTGCGGGTATAAATGATCTCGTC
	A	GACGAGATCATTTATACCCGCAACGTATCCACTGGCTGG
RN-tre R-A	S	CAAATAGACCTGGATGTCAACGCCACATTTCGGGACCACATTATG
	A	CATAATGTGGTCCCGAAATGTGGCGTTGACATCCAGGTCTATTTG
RN-tre Q-A	S	CACGGAAGTCGGGTATTGTGCGGGGATGGACCAGATCACAG
	A	CTGTGATCTGGCTCATCCCCGCACAATACCCGACTTCCGTG
TBC proteins cloned into the dsRed monomer-C1 vector		
TBC1D5	S	ATGCCTCGAGCTTATCATTCCTTATCTGAAACTAGACATCC
	A	ATGCGGATCCTCAGATGTCCAGGGGACTCACAATGGT
TBC1D6	S	ATGCGAATTCTCAGCCCGCCGAGCGCTCGCGG
	A	ATGCGGATCCTCATGCCCACTGGTGCCCGTC
TBC1D8B	S	ATGCGAATTCTGCTACTAATCCTGACTATTATAC
	A	ATGCGGATCCTCAGCAGCCTCTGAACTGCTGGC
TBC1D20	S	ATGCCTCGAGCTGCCCTCCGGAGTGCGCAGGGC
	A	ATGCGAATTCTCAGGGAAACAGCTGCAGCTGAAA
TBC1D22A	S	GATCGAATTCTGCCAGCGACGGGGCCAGGAAG
	A	GATCGGATCCTCATTTCTTGTAGTGATTGGGGGC
RN-tre	S	ATGCGAATTCTAATTCAGACCAGGATGTAGCACTC
	A	ATGCGGATCCTCACAGCAACACTGACTCTTGGATGG
TBC1D6 domains cloned into the pEGFP-C1 vector		
N-terminus	S	ATGCGAATTCTCAGCCCGCCGAGCGCTCGCGG
	A	ATGCGGATCCTCAATAGCGCTTCACTGTCCGGCTC
TBC	S	ATGCGAATTCTGTCCGGAAAGGGGTCCCGC
	A	ATGCGGATCCTCACAAAATCAACTCCTGGTGCTGCT
C-terminus	S	ATGCGAATTCTGAAGCCACCAGCGTTCCAGAC
	A	ATGCGGATCCTCATGCCCACTGGTGCCCGTC
1D6-N-terminus	S	ATGCGAATTCTGTCCGGAAAGGGGTCCCGC
	A	ATGCGGATCCTCATGCCCACTGGTGCCCGTC
1D6-C-terminus	S	ATGCGAATTCTCAGCCCGCCGAGCGCTCGCGG
	A	ATGCGGATCCTCACAAAATCAACTCCTGGTGCTGCT

TBC and Rab proteins cloned into the pEGX-4T-1 vector		
TBC1D6	S	GATCGGATCCCAGCCCGCCGAGCGCTCGCGG
	A	GATCCTCGAGTGCCCACTGGTGCCCGTCCTG
1D6-C-terminus		GATCGGATCCCAGCCCGCCGAGCGCTCGCGG
		ATGCCTCGAGCAAAATCAACTCCTGGTGCTGCT
Rab26	S	ATGCGGATCCTCCAGGAAGAAGACCCCCAAGA
	A	ATGCGAATTCTCAAGGGCGGCAGCAG
Rab33 mutants		
Rab33aN151I	S	AAAGTGCTTGTGGGCATCAAGTGTGACTTGAGG
	A	CCTCAAGTCACACTTGATGCCCACAAGCACTTT
Rab33bN148I	S	CCTCGAATTCTGGTGGGAATTAAATGTGACTTGAGAAGC
	A	GCTTCTCAAGTCACATTTAATTCCCACCAGAATTCGAGG
