# Supplementary material for: Specific TBC Domain-Containing Proteins Control the ER-Golgi-Plasma Membrane Trafficking of GPCRs
Source: Cell Rep. Author manuscript; Available in PMC 2019 Jul 18. (PMC6639060; doi:10.1016/j.celrep.2019.05.033)
Supplement: 2 [file NIHMS1534188-supplement-2.pdf]

Cell Reports

Article

Specific TBC Domain-Containing Proteins Control the ER-Golgi-Plasma Membrane Trafficking of GPCRs

Graphical Abstract

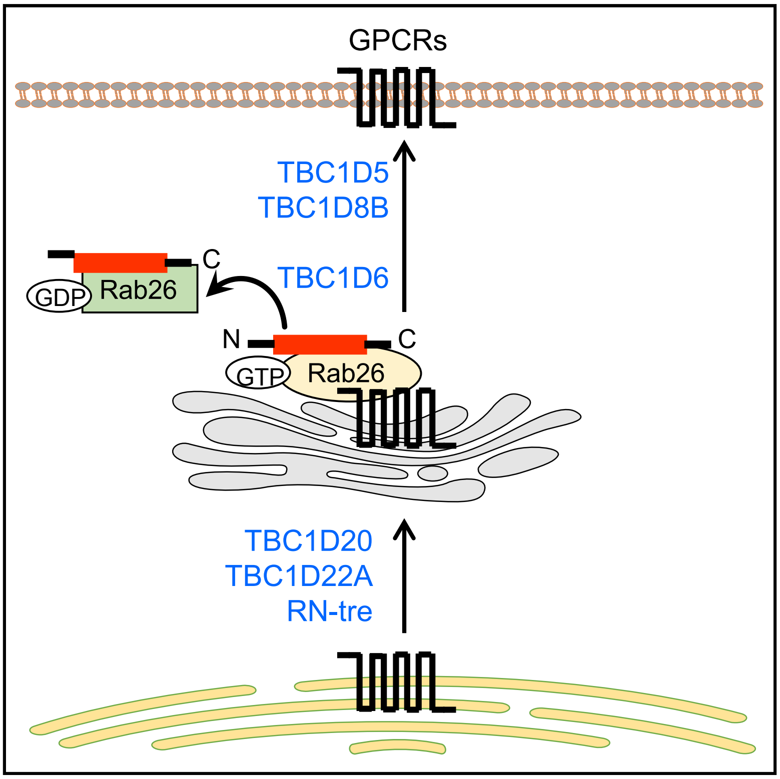

Highlights

- Six TBC proteins activity-dependently regulate the cell surface export of GPCRs
- TBC proteins selectively control ER-Golgi or Golgi-cell surface transport of GPCRs
- TBC1D6 is a GAP for Rab26 and physically associates with Rab26
- TBC1D6 modulates Rab26 interaction with and the post-Golgi traffic of GPCRs

Authors

Zhe Wei, Maoxiang Zhang, Chunman Li, ..., Zheng Dong, Gang Hu, Guangyu Wu

Correspondence

guwu@augusta.edu

In Brief

Wei et al. report that several TBC proteins specifically and activity-dependently regulate ER-Golgi-plasma membrane transport of nascent GPCRs. They also show that TBC1D6 is a GAP for Rab26 and controls GPCR post-Golgi traffic. Their results reveal crucial roles of TBC proteins in and provide regulatory mechanisms of GPCR trafficking.

Wei et al., 2019, Cell Reports *28*, 554–566
July 9, 2019 © 2019 The Author(s).
https://doi.org/10.1016/j.celrep.2019.05.033

OPEN ACCESS
CellPress

Cell Reports
Article

Specific TBC Domain-Containing Proteins Control the ER-Golgi-Plasma Membrane Trafficking of GPCRs

Zhe Wei,[1,6] Maoxiang Zhang,[1,6] Chunman Li,[1,6] Wei Huang,[1] Yi Fan,[1] Jianhui Guo,[1] Mostafa Khater,[1] Mitsunori Fukuda,[2] Zheng Dong,[3,4] Gang Hu,[5] and Guangyu Wu[1,7,*]
[1]Department of Pharmacology and Toxicology, Medical College of Georgia, Augusta University, Augusta, GA 30912, USA
[2]Department of Integrative Life Sciences, Graduate School of Life Sciences, Tohoku University, Sendai, Miyagi, Japan
[3]Department of Cell Biology and Anatomy, Medical College of Georgia, Augusta University, Augusta, GA 30912, USA
[4]Charlie Norwood VA Medical Center, Augusta, GA 30904, USA
[5]Department of Pharmacology, Nanjing University of Chinese Medicine, Nanjing, China
[6]These authors contributed equally
[7]Lead Contact
*Correspondence: guwu@augusta.edu
https://doi.org/10.1016/j.celrep.2019.05.033

SUMMARY

G-protein-coupled receptors (GPCRs) constitute the largest superfamily of cell surface signaling proteins. However, the molecular mechanisms underlying their cell surface delivery after synthesis remain poorly understood. Here, we screen the TBC domain-containing proteins, putative Rab GTPase-activating proteins (GAPs), in the intracellular trafficking of GPCRs and identify several TBC proteins that activity-dependently regulate the anterograde transport, *en route* from the endoplasmic reticulum to the Golgi or from the Golgi to the cell surface, of several prototypic GPCR members without affecting other plasma membrane proteins. We also show that TBC1D6 functions as a GAP for Rab26, physically associates with Rab26, and attenuates Rab26 interaction with GPCRs. Furthermore, both overexpression and depletion of TBC1D6 inhibit the post-Golgi traffic of GPCRs. These data demonstrate important roles of the TBC proteins in forward trafficking of nascent GPCRs and reveal regulatory mechanisms of GPCR targeting to the functional destination.

INTRODUCTION

Rab GTPases are the master regulators of vesicle-mediated membrane traffic and regulate almost every trafficking step involved in endocytosis and exocytosis (Pfeffer, 2017; Stenmark, 2009). More than 60 Rab GTPases have been identified in mammals, forming the largest branch of the Ras-related GTPase. As with all GTPases, the function of Rab GTPases is crucially regulated by their cycle between an inactive GDP-bound state and an active GTP-bound state that are under control by two proteins: a guanine nucleotide exchange factor (GEF) that accelerates the release of GDP from Rabs and a GTPase-activating protein (GAP) that enhances the intrinsically low GTP hydrolysis rate of GTP-bound Rab. In the GDP-bound state, Rab GTPases are extracted from membranes by GDP dissociation inhibitors that regulate both Rab expression in the cytoplasm and recruitment onto the correct subcellular location. In the GTP-bound state, Rab GTPases can bind to diverse downstream effectors to ensure proper vesicle sorting, motility, tethering, and fusion with the appropriate membranes (Grosshans et al., 2006).

Recent studies have shown that Tre2-Bub2-Cdc16 (TBC) domain-containing proteins are specific GAPs for Rab GTPases (Barr and Lambright, 2010; Frasa et al., 2012; Fukuda, 2011). TBC domain is a conserved catalytic domain that consists of ~200 amino acid residues. Since the identification of the TBC domain in yeast Rab GAPs (Albert et al., 1999), a number of TBC proteins have been characterized in mammalian cells. Based on the sequence homology of the TBC domain, more than 40 TBC proteins are predicted to exist in humans. Some TBC proteins have been demonstrated to act on specific Rabs and to regulate specific trafficking steps and cellular processes same as their respective target Rabs (Davey et al., 2012; Fuchs et al., 2007; Goueli et al., 2012; Haas et al., 2005, 2007; Hsu et al., 2010; Itoh and Fukuda, 2006; Itoh et al., 2011; Peralta et al., 2010; Yoshimura et al., 2007). The most TBC proteins use a dual Arg-Gln-finger catalytic mechanism to inactivate Rab substrates (Du and Novick, 2001; Pan et al., 2006) that is largely different from the single Arg finger mechanism used by many other small GTPase GAPs.

G-protein-coupled receptors (GPCRs) modulate a wide variety of physiological and pathological functions and are the therapeutic targets of approximately one-third of the drugs on the market (Hauser et al., 2017; Hilger et al., 2018; Pierce et al., 2002). For most GPCRs, the plasma membrane represents the most important functional destination where they bind to their respective ligands and activate cognate heterotrimeric G proteins, arrestins, and other signaling proteins that in turn activate downstream effectors. However, as compared with extensively studied internalization, recycling, and degradation pathways (Hanyaloglu and von Zastrow, 2008; Marchese et al., 2008), the molecular mechanisms underlying the anterograde transport of nascent GPCRs to the plasma membrane, *en route* from the endoplasmic reticulum (ER) where they are synthesized and through the Golgi where the receptors attain fully matured statues, remain poorly understood.

A number of Rab GTPases have been well studied to regulate the cell surface transport, internalization, recycling, degradation,

 © 2019 The Author(s).
This is an open access article under the CC BY license (http://creativecommons.org/licenses/by/4.0/).

and nuclear translocation of a number of GPCRs (Bhosle et al., 2016; Dale et al., 2004; Fan et al., 2003; Filipeanu et al., 2006; Li et al., 2012, 2017; Wang and Wu, 2012; Wu et al., 2003). However, the functions of TBC proteins in any trafficking processes of the GPCR superfamily have not been investigated. In this study, we identify six TBC proteins to activity-dependently regulate the anterograde ER-Golgi-cell surface traffic of GPCR members. We also show that TBC1D6 controls the post-Golgi transport of GPCRs via inactivating Rab26 and inhibiting Rab26 interaction with the receptors. These results provide a previously unappreciated function of TBC proteins, by virtue of their abilities to regulate both Rab GTPase activation and Rab-GPCR interaction, in the intracellular trafficking of GPCR members and reveal regulatory mechanisms of targeting to the functional destination of nascent GPCRs, a poorly explored area in the study of the GPCR superfamily.

RESULTS

Six TBC Proteins Regulate the Cell Surface Delivery and Signaling of α_{2B}-Adrenergic Receptor (AR) in an Activity-Dependent Manner

As an initial approach to study the possible functions of TBC proteins in regulating GPCR trafficking, we used α_{2B}-AR, a prototypic GPCR, as a model to screen for TBC proteins involved in the anterograde cell surface transport using stable HEK293 cells expressing N-terminal hemagglutinin (HA)-tagged α_{2B}-AR. Forty-three TBC domain-containing proteins and Rab3GAP, the known Rab3 GAP without a TBC domain, were tagged with GFP and their expression was visualized by confocal microscopy (Figure S1A). The effects of TBC proteins on the cell surface transport of α_{2B}-AR at steady state were measured by intact live cell ligand binding using the cell nonpermeable radioligand ^{3}H-RX821002. This strategy identified six TBC proteins, namely TBC1D5, TBC1D6, TBC1D8B, TBC1D20, TBC1D22A, and RN-tre, which significantly inhibited the cell surface numbers of α_{2B}-AR by $\sim$30% as compared with cells expressing the control vector pEGFP-C1 (Figure 1A). In contrast, other 37 TBC proteins and Rab3GAP did not have clear effects on the cell surface expression of α_{2B}-AR (Figure 1A). The expression of TBC proteins and Rab3GAP did not influence the total expression of α_{2B}-AR as measured by flow cytometry following staining with HA antibodies in permeabilized cells (Figure 1A). Furthermore, expression of arrestin-3(201-409), a dominant-negative mutant of arrestin-3 that mediates agonist-stimulated internalization of a number of GPCRs including α_{2B}-AR (DeGraff et al., 1999), did not reverse the effect of TBC proteins on the cell surface expression of α_{2B}-AR (Figure S1B), suggesting that the reduction of α_{2B}-AR expression at the cell surface induced by TBC proteins was not caused by enhanced internalization of the receptor.

We then determined the effect of the six TBC proteins on the cell surface transport of newly synthesized α_{2B}-AR in inducible HEK293 cells generated by using the Tet-On 3G tetracycline inducible gene expression system. The inducible cells were first transiently transfected with individual TBC proteins and then incubated with doxycycline at a concentration of 40 ng/mL for 24 h to achieve the maximal cell surface expression of the receptor (Li et al., 2017; Zhang et al., 2016a). Expression of each of the six TBC proteins significantly attenuated the cell surface expression of α_{2B}-AR (Figure 1B). Similarly, transient expression of these TBC proteins inhibited the cell surface expression of endogenous α_{2B}-AR by 30%–50% in MCF-7 cells (Figures 1C and S1C). These data demonstrate that the TBC proteins TBC1D5, TBC1D6, TBC1D8B, TBC1D20, TBC1D22A, and RN-tre are able to regulate the cell surface transport of α_{2B}-AR.

The six TBC proteins identified above to regulate the cell surface transport of α_{2B}-AR are canonical TBC proteins containing two catalytic residues, an Arg finger and a Gln finger (Figure 1D). To define if their inhibitory effects on α_{2B}-AR transport were specifically mediated through inactivating Rab GTPases, we compared the effects of wild type TBC proteins with their respective catalytically inactive mutants in which both the Arg and Gln fingers were mutated to Ala (RQ-AA). Confocal microscopy revealed that RQ mutations did not alter the subcellular localization of these TBC proteins (Figure S1D). Immunoblotting showed that, as compared with their RQ-AA mutants, the six TBC proteins were expressed at similar levels with predicted apparent molecular weights (Figure S1E). In marked contrast to wild type TBC counterparts that blocked the cell surface transport of α_{2B}-AR, expression of the RQ-AA mutants had no significant effects on α_{2B}-AR expression at the cell surface measured by radioligand binding (Figure 1E). These data suggest that the function of these TBC proteins in regulating α_{2B}-AR transport is dependent on their GAP activity.

We next addressed the question if TBC protein-induced reduction of the cell surface α_{2B}-AR could result in a concomitant defective signaling. α_{2B}-AR couples to the Gi/Go family G proteins, and its activation has been well shown to stimulate the mitogen-activated protein kinases (MAPK) ERK1/2, inhibit adenylyl cyclases, and suppress voltage-gated calcium channel (Dong et al., 2011). The activation of ERK1/2 was chosen as a functional readout, and TBC1D6 was chosen as a representative TBC protein to be studied. Consistent with its ability to inhibit α_{2B}-AR cell surface transport, the expression of TBC1D6 significantly attenuated ERK1/2 activation in response to UK14304 stimulation by 55% in cells stably expressing α_{2B}-AR, whereas its mutant RQ-AA had no effect (Figures 1F and 1G). To eliminate the possibility that the effect of TBC1D6 on the MAPK activation was mediated through the inhibition of the activation of G proteins and/or other signaling molecules, we measured the effect of TBC1D6 on MAPK activation by 1,3-bis(4-amino-2-methylquinolin-6-yl)urea (NSC12155) that activates MAPK via enhancing the release of $G\beta\gamma$ from the $G\alpha\beta\gamma$ trimers (Surve et al., 2014). Treatment with NSC12155 remarkably activated MAPK that was not affected by expression of TBC1D6 and its inactive mutant (Figures S1F and S1G). These data suggest that TBC1D6 does not influence the activation of G proteins and other signaling molecules downstream of G proteins involved in the MAPK activation, and TBC proteins regulate not only α_{2B}-AR trafficking but also its function in an activity-dependent fashion.

Specific TBC Proteins Control the ER-to-Golgi and the Golgi-to-Plasma Membrane Transport of α_{2B}-AR

To confirm the effects of the six TBC proteins on the cell surface expression of α_{2B}-AR measured by radioligand binding, confocal microscopy was utilized to directly visualize subcellular

OPEN
ACCESS
CellPress

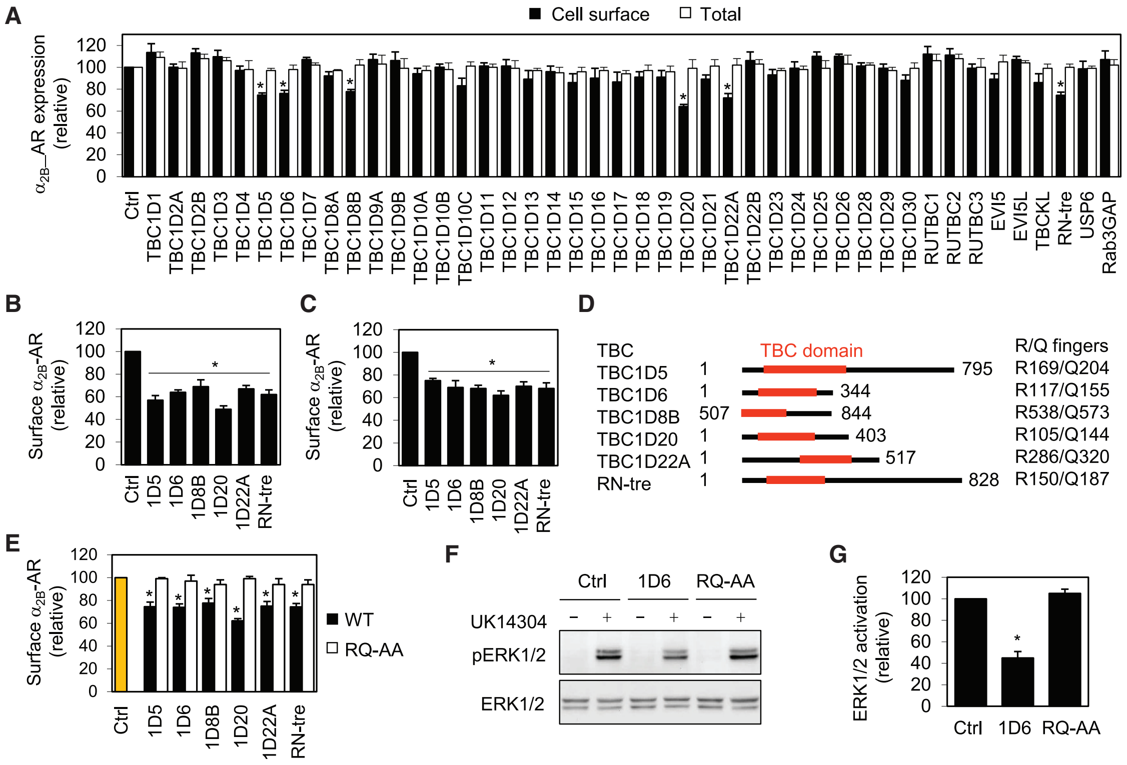

Figure 1. Six TBC Proteins Regulate the Cell Surface Expression of α_{2B}-AR in an Activity-Dependent Fashion

(A) Screening for TBC proteins involved in the surface transport of α_{2B}-AR. HEK293 cells stably expressing HA-α_{2B}-AR were transfected with the control vector pEGFP-C1 (Ctrl) or individual GFP-TBC proteins. The cell surface expression of α_{2B}-AR was determined by intact cell ligand binding using [^{3}H]-RX821002. The mean value of specific radioligand binding was 23,432 ± 1,432 cpm from cells transfected with the control vector.

(B) Inhibition of the surface transport of inducibly expressed α_{2B}-AR by TBC proteins. Stable HEK293 cells inducibly expressing HA-α_{2B}-AR were transfected with pEGFP-C1 (Ctrl) or GFP-TBC proteins, and the cell surface expression of the receptor was measured by radioligand binding after incubation with doxycycline (40 ng/mL) for 24 h. In a typical experiment, the maximal specific binding of ^{3}H-RX821002 are 21,562 cpm/well (24-well dish) in control cells.

(C) Inhibition of the surface expression of endogenous α_2-AR by TBC proteins in MCF-7 cells. The mean value of specific ligand binding was 762 ± 143 cpm in control cells.

(D) A diagram showing the locations of the TBC domain (red) in six TBC proteins. The right panel indicates the exact positions of the Arg and Gln fingers.

(E) Effect of six TBC proteins and their RQ-AA mutants on the cell surface expression of α_{2B}-AR as measured by intact cell ligand binding.

(F) Effect of TBC1D6 and its RQ-AA mutant on α_{2B}-AR-mediated ERK1/2 activation. HEK293 cells stably expressing α_{2B}-AR were transfected with the pEGFP-C1 vector (Ctrl) or GFP-TBC1D6. The cells were stimulated with UK14304 at 1 μM for 5 min and ERK1/2 activation was determined by immunoblotting.

(G) Quantitative data shown in (F).

The quantitative data are presented as mean ± SE (n = 3–4). *$p < 0.05$ versus Ctrl.

distribution of α_{2B}-AR in cells expressing individual TBC proteins. For this purpose, α_{2B}-AR was tagged with GFP, whereas the six TBC proteins were tagged with dsRed. As expected, GFP-tagged α_{2B}-AR was robustly expressed at the cell surface in cells transfected with the control vector dsRed. The expression of TBC1D5, TBC1D6, TBC1D8B, TBC1D20, TBC1D22A, and RN-tre each caused a marked reduction in the cell surface presentation of α_{2B}-AR that was accompanied by an extensive accumulation of the receptors in the perinuclear region, whereas the expression of TBC mutants did not alter the cell surface expression of α_{2B}-AR (Figure 2A). The effects of the six TBC proteins on the subcellular distribution of α_{2B}-AR were markedly different. Whereas the subcellular localizations of α_{2B}-AR were similar in cells expressing TBC1D5, TBC1D6, and TBC1D8, they are apparently different from those in cells expressing TBC1D20, TBC1D22A, and RN-tre (Figure 2A).

To define the intracellular compartments in which individual TBC proteins regulate α_{2B}-AR transport, we first studied the colocalization of α_{2B}-AR with different intracellular organelle marker proteins in cells expressing TBC1D6, TBC1D20, and TBC1D22A. α_{2B}-AR was strongly colocalized with the ER marker calregulin in cells expressing TBC1D20 and TBC1D22A (Figure 2B), whereas it was clearly colocalized with the *cis*-Golgi marker GM130 in cells expressing TBC1D6 (Figure 2C). These data suggest that TBC1D20 and TBC1D22A control α_{2B}-AR transport from the ER to the Golgi, whereas likely TBC1D6 regulates α_{2B}-AR transport from the Golgi to the plasma membrane.

We next took advantage of our previously characterized Golgi-localized YS mutant of α_{2B}-AR (Dong and Wu, 2006) and determined if TBC1D6, TBC1D20, and TBC1D22A could block its transport from the ER to the Golgi. The mutant

OPEN
ACCESS
CellPress

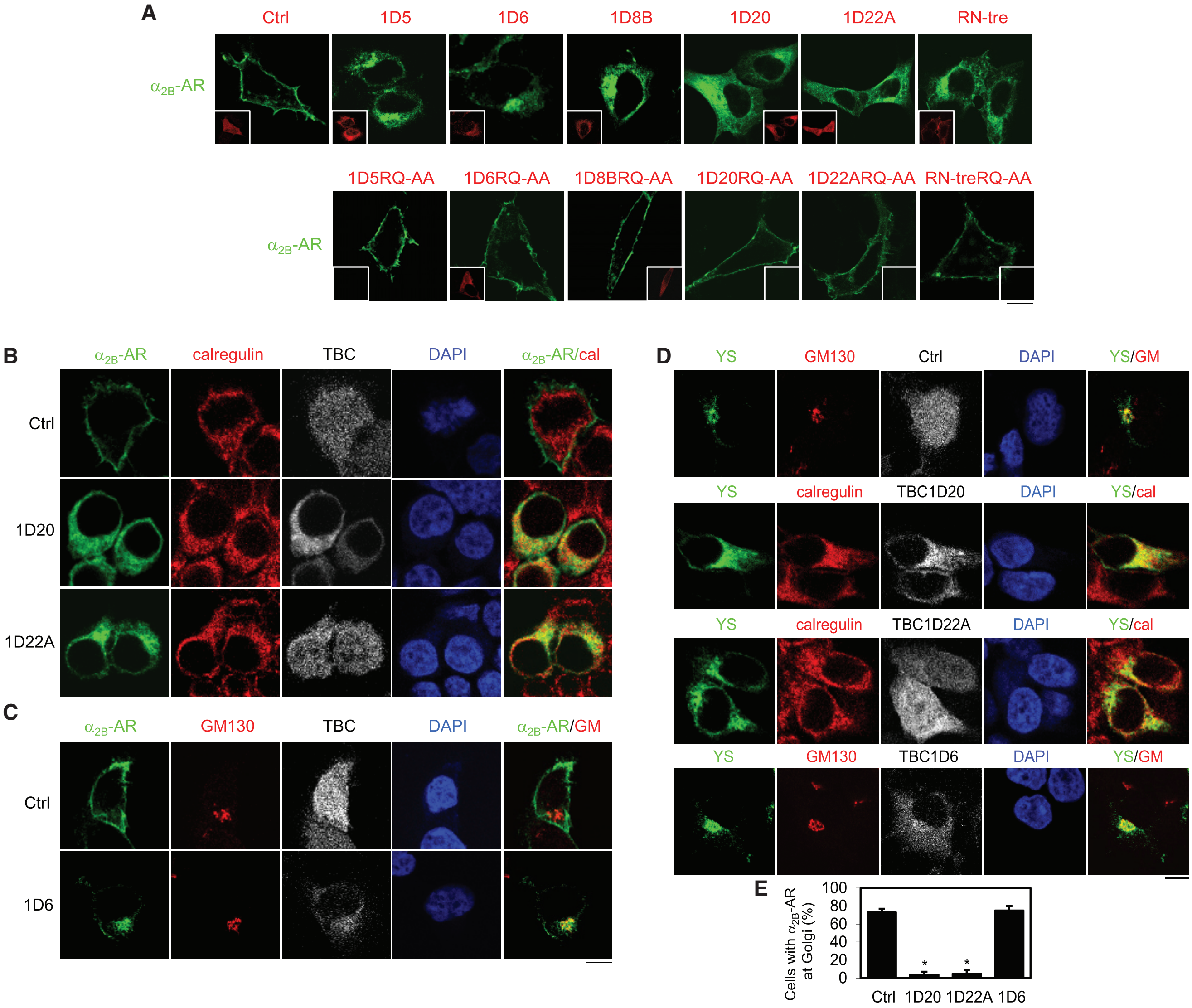

Figure 2. Inhibition of α_{2B}-AR Transport from the ER through the Golgi to the Cell Surface by TBC Proteins

(A) Effect of the six TBC proteins and their inactive RQ-AA mutants on the subcellular distribution of α_{2B}-AR. HEK293 cells were transfected with α_{2B}-AR-GFP together with the vector dsRed-C1 (Ctrl) or individual dsRed-TBC proteins. The subcellular distribution of α_{2B}-AR was revealed by confocal microscopy. Insets show the expression of individual TBC proteins.

(B) Colocalization of α_{2B}-AR with the ER marker calregulin in cells expressing TBC1D20 and TBC1D22A. HEK293 cells were transfected with α_{2B}-AR-GFP together with dsRed-C1 (Ctrl) or individual dsRed-tagged TBC proteins and then stained with calregulin antibodies.

(C) Colocalization of α_{2B}-AR with the Golgi marker GM130 in cells expressing TBC1D6. HEK293 cells were transfected with α_{2B}-AR-GFP together with dsRed-C1 (Ctrl) or individual dsRed-tagged TBC proteins and then stained with GM130 antibodies.

(D) Effect of TBC proteins on the ER-to-Golgi transport of the α_{2B}-AR YS mutant. HEK293 cells were transfected with GFP-tagged YS mutant together with dsRed-C1 (Ctrl) or dsRed-TBC proteins. The cells were then stained with GM130 antibodies in cells expressing control vector and TBC1D6 or with calregulin antibodies in cells expressing TBC1D20 and TBC1D22A.

(E) Quantitative data shown in (D). Approximately 100 cells were counted in each experiment.

The quantitative data are mean ± SE (n = 3). *$p < 0.05$ versus Ctrl. The images shown are representatives of at least 3 experiments. Scale bars, 10 μm.

YS-AA was mainly retained in the Golgi in cells expressing the control vector dsRed and TBC1D6, but it was clearly arrested in the ER, unable to export to the Golgi, in cells expressing TBC1D20 and TBC1D22A (Figures 2D and 2E). This further indicates a role for TBC1D20 and TBC1D22A in the ER-to-Golgi transport of α_{2B}-AR.

Multiple TBC Proteins Selectively Modulate the Cell Surface Export of GPCRs, but Not Other Plasma Membrane Proteins

We then sought to determine if the six TBC proteins identified above were able to regulate the other three family A GPCRs, α_{2A}-AR, β_2-AR, and angiotensin II type 1 receptor (AT1R), in

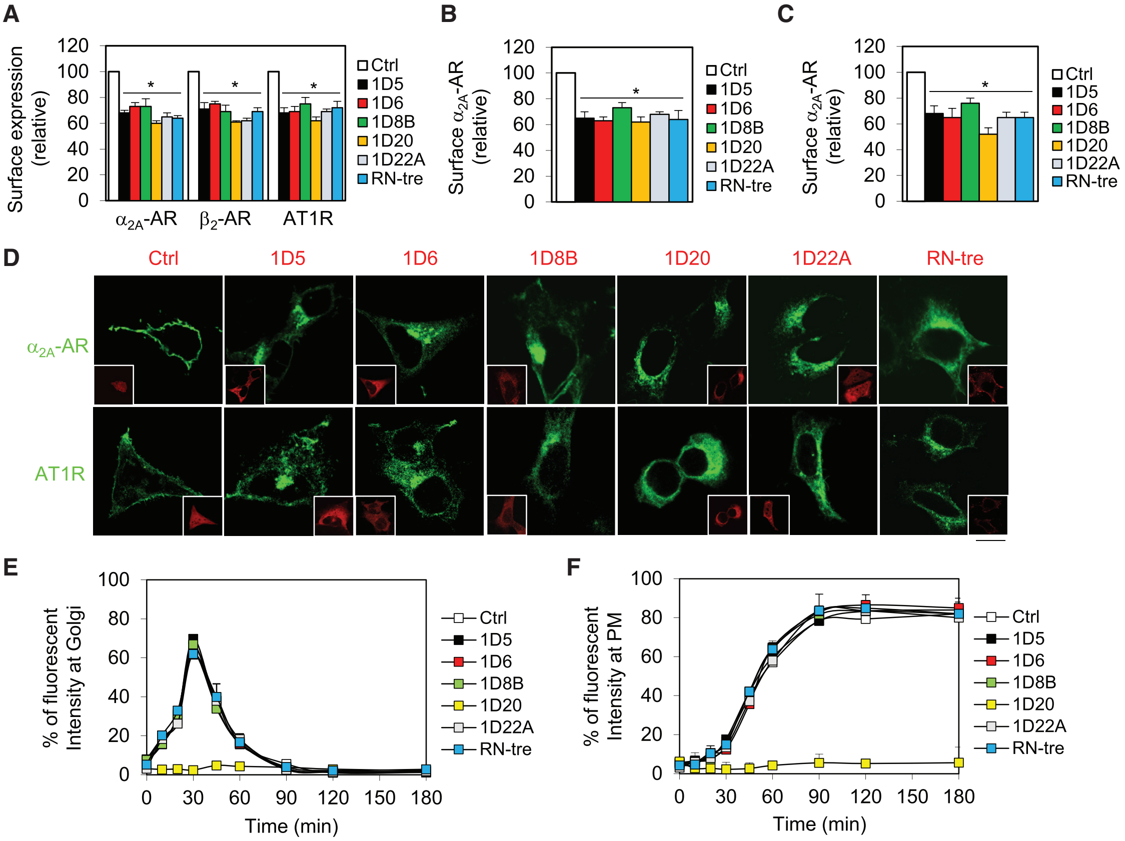

Figure 3. Effect of Six TBC Proteins on the Cell Surface Traffic of GPCRs and Other Plasma Membrane Proteins

(A) Effect of TBC proteins on the surface expression of α_{2A}-AR, β_2-AR, and AT1R. HEK293 cells were transfected with individual receptors together with pEGFP-C1 (Ctrl) or GFP-TBC proteins and the cell surface expression of each receptor was measured by radioligand binding or flow cytometry.

(B) Inhibition of the surface transport of inducibly expressed α_{2A}-AR by TBC proteins. Stable HEK293 cells inducibly expressing HA-α_{2A}-AR were transfected with pEGFP-C1 (Ctrl) or GFP-TBC proteins and the cell surface expression of the receptor was measured by radioligand binding after doxycycline induction for 24 h. In a typical experiment, the maximal specific binding of ^{3}H-RX821002 are 24,805 cpm/well (24-well dish) in control cells.

(C) Inhibition of the surface expression of endogenous α_{2A}-AR by TBC proteins in HT29 cells. The mean value of specific ligand binding was 892 ± 187 cpm in control cells.

(D) Effect of six TBC proteins on the subcellular distribution of α_{2A}-AR and AT1R. HEK293 cells were transfected with α_{2A}-AR-GFP or AT1R-GFP together with the vector dsRed-C1 (Ctrl) or individual dsRed-TBC proteins. The subcellular distribution of the receptors was revealed by confocal microscopy.

(E) Effect of TBC proteins on the expression of VSVG at the Golgi. HEK293 cells were transfected with VSVGtsO45-GFP together with dsRed-C1 (Ctrl) or dsRed-tagged individual TBC proteins. The cells were cultured at 40°C for 24 h (0 min) and then shifted to 32°C for 10, 20, 30, 45, 60, 90, 120, and 180 min. The expression of VSVG at the Golgi was measured by fluorescent intensity in a total of 25–30 cells.

(F) Effect of TBC proteins on the expression of VSVG at the plasma membrane (PM) as described in (E).

The data are mean ± SE (n = 3–7). *$p < 0.05$ versus Ctrl. The images are representatives of 3 experiments. Scale bar, 10 μm.

transient expression systems. Similar to their effects on α_{2B}-AR, the expression of TBC1D5, TBC1D6, TBC1D8B, TBC1D20, TBC1D22A, and RN-tre each significantly inhibited the cell surface expression of α_{2A}-AR, β_2-AR, and AT1R by 25%–45% as measured by radioligand binding or flow cytometry (Figure 3A). The expression of these six TBC proteins also significantly attenuated the cell surface expression of inducibly expressed α_{2A}-AR in HEK293 cells (Figure 3B). Furthermore, the expression of each of these six TBC proteins reduced the cell surface expression of endogenous α_{2A}-AR in HT-29 cells (Figures 3C and S2A).

We then studied the effect of the six TBC proteins on the subcellular localization of α_{2A}-AR and AT1R. The expression of each of the six TBC proteins arrested both receptors in the perinuclear region revealed by confocal microscopy (Figure 3D). In contrast, among the six TBC proteins, only TBC1D20 was found to activity-dependently inhibit the cell surface expression of epidermal growth factor receptor (EGFR) as measured by quantification of fluorescent intensity, whereas TBC1D5, TBC1D6, TBC1D8B, TBC1D22A, and RN-tre had no effect (Figures S2B and S2C). Consistently, expression of TBC1D6 had no effect of the activation of MAPK by EGF (Figure S2D and S2E).

Similar to EGFR, the transport of vesicular stomatitis virus glycoprotein (VSVG) using its temperature-sensitive mutant (VSVGtsO45) from the ER to the Golgi and to the cell surface was markedly inhibited by TBC1D20, but not its RQ-AA mutant and other five TBC proteins (Figures 3E, 3F, and S2F). These data suggest that the actions of TBC1D5, TBC1D6, TBC1D8B,

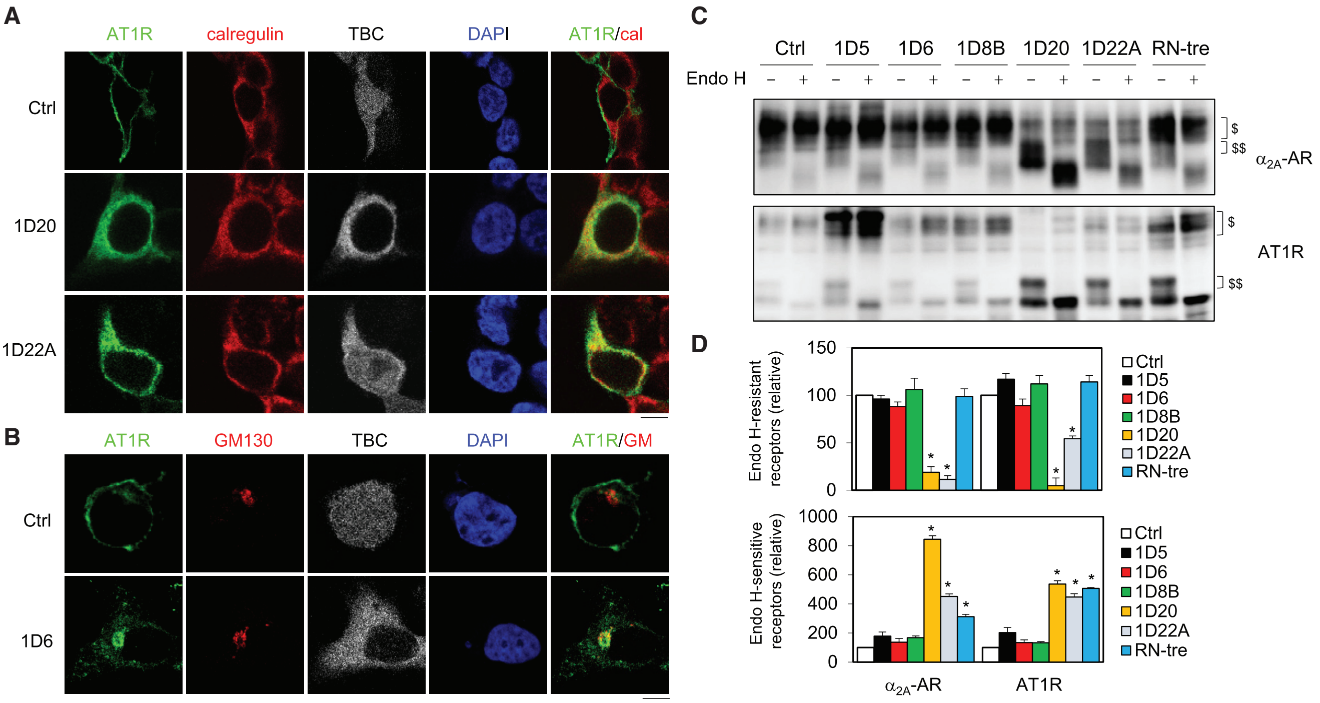

Figure 4. Effect of TBC Proteins on the Subcellular Distribution and Glycosylation of GPCRs

(A) Colocalization of AT1R with calregulin in cells expressing TBC1D20 and TBC1D22A.

(B) Colocalization of AT1R with GM130 in cells expressing TBC1D6.

(C) Deglycosylation of α_{2A}-AR (upper panel) and AT1R (lower panel) by Endo H treatment. $ indicates Endo H-resistant bands and $$ Endo H-sensitive bands.

(D) Quantitative data showing the effect of TBC proteins on the formation of Endo H-resistant (upper panel) and Endo H-sensitive glycosylation (lower panel) of the receptors.

The quantitative data are mean ± SE (n = 3–5). *$p < 0.05$ versus Ctrl. The images shown are representatives of 4 experiments. Scale bars, 10 μm.

TBC1D22A, and RN-tre on the transport of GPCRs are likely specific.

Confocal microscopy revealed that, similar to α_{2B}-AR, AT1R was remarkably colocalized with calregulin in cells expressing TBC1D20 and TBC1D22A (Figure 4A), whereas it was extensively colocalized with GM130 in cells expressing TBC1D6 (Figure 4B).

We next defined the effect of the six TBC proteins on the conversion from simple to complex glycosylation of GPCRs that reflects the transport of the receptors from the ER to the Golgi. α_{2B}-AR does not have N-linked glycosylation sites, whereas α_{2A}-AR and AT1R possess 2 and 3 N-linked glycosylation sites at their N termini, respectively, and therefore were chosen to be studied. The expression of TBC1D20, TBC1D22A, and RN-tre markedly enhanced the formation of endoglycosidase (Endo) H-sensitive glycosylation of both receptors (Figures 4C and 4D). However, TBC1D20 and TBC1D22A, but not RN-tre, inhibited the acquisition of complex N-linked sugars (Endo H-resistant) (Figures 4C and 4D) that was further confirmed by PNGase F treatment to remove both simple and complex glycosylation (Figure S3). In contrast, TBC1D5, TBC1D6, and TBC1D8B did not affect both Endo H-sensitive and -resistant glycosylation of the receptors. These data further suggest that TBC1D20, TBC1D22A, and RN-tre, but not TBC1D5, TBC1D6, and TBC1D8B, may play a role in GPCR transport from the ER to the Golgi.

TBC1D6 Is a Specific GAP for Rab26 and Regulates Rab26 Interaction with GPCRs

Our previous studies have demonstrated that Rab26 and three Golgi-localized, γ-adaptin ear domain homology, ADP ribosylation factor-binding proteins (GGAs) regulate the Golgi-to-plasma membrane transport of GPCRs (Li et al., 2012; Zhang et al., 2016a, 2016b). They all directly interacted with α_{2B}-AR, specifically its third intracellular loop (ICL3), and the RRR motif was identified as the GGA3-binding motif in the ICL3 (Figure S4A). However, mutation of the RRR motif did not affect Rab26 interaction with the ICL3 (data not shown), and progressive deletion of the ICL3 failed to identify a domain interacting with Rab26 (Figures S4B and S4C), suggesting that the conformation of the loop may be important for Rab26 interaction. Furthermore, small interfering RNA (siRNA)-mediated depletion of Rab26 inhibited the cell surface transport of α_{2A}-AR, α_{2B}-AR, and an α_{2B}-AR mutant in which the RRR motif was mutated to Ala, whereas knockdown of GGA3 by siRNA only attenuated the cell surface expression of α_{2B}-AR, but not the RRR mutant and α_{2A}-AR (Figure S4D). In addition to α_{2B}-AR, the α_{2A}-AR ICL3 and the AT1R C terminus also strongly bound to Rab26 as measured in GST fusion protein pull-down assays (Figure S4E). These data demonstrate that Rab26 and GGAs differentially regulate GPCR transport (Figure S4F). These data also suggest that Rab26 and GGAs likely control the post-Golgi transport of GPCRs through distinct routes.

OPEN ACCESS
CellPress

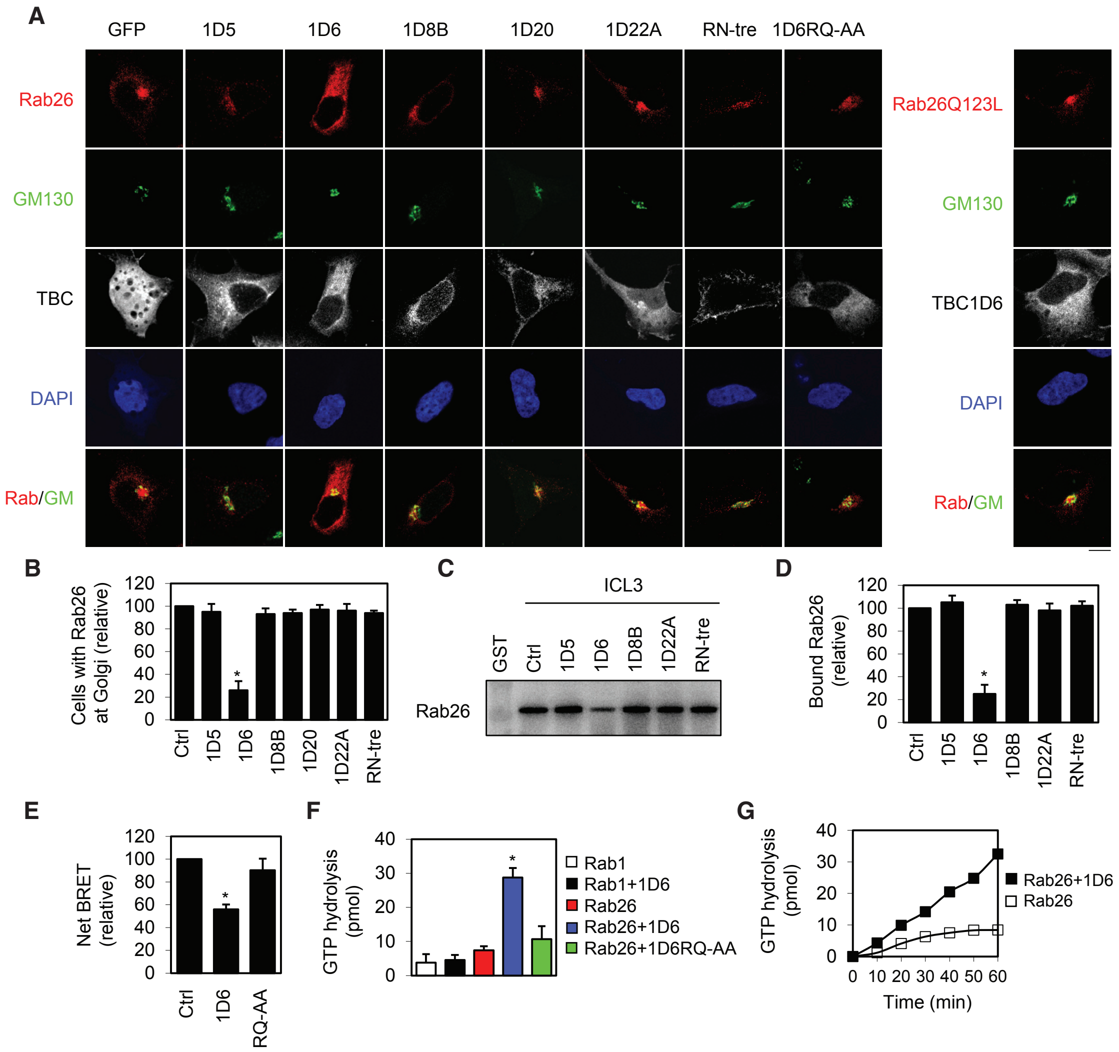

Figure 5. Effect of TBC Proteins on the Subcellular Localization, Interaction with α_{2B}-AR, and GTP Hydrolysis of Rab26

(A) Effect of the six TBC proteins on the subcellular localization of Rab26. HEK293 cells were transfected with individual GFP-tagged TBC proteins together with dsRed-tagged Rab26 or the active mutant Rab26Q123L and then stained with GM130 antibodies.

(B) Quantitative data shown in (A). The data are percentage of cells with Rab26 expression at the Golgi with a total of 50 cells counted in each experiment.

(C) Effect of the six TBC proteins on Rab26 interaction with the ICL3 of α_{2B}-AR in GST fusion protein pull-down assays.

(D) Quantitative data shown in (C).

(E) Effect of TBC1D6 and its inactive mutant on Rab26 interaction with full-length α_{2B}-AR in BRET assays.

(F) Effect of TBC1D6 on GTP hydrolysis by Rab26 and Rab1 measured in GAP assays. GST-tagged Rabs were loaded with [γ-^{32}P]-GTP and then incubated with TBC1D6 or its QR-AA mutant.

(G) Time-dependent action of TBC1D6 on GTP hydrolysis by Rab26. Similar results were obtained in 2 different experiments.

The quantitative data are mean ± SE (n = 3–5). *$p < 0.05$ versus Ctrl. The images shown are representatives of 3 experiments. Scale bars, 10 μm.

Because the GAPs for Rab26 have not been identified, we hypothesized that at least one of the six TBC proteins identified above was a specific GAP for Rab26. To test this hypothesis, we first determined the effect of the six TBC proteins on the subcellular localization of Rab26 by confocal microscopy. Rab26, as well as its constitutively active mutant Q123L, localizes to the Golgi and partially colocalizes with GM130, whereas its inactive mutants are mainly expressed in cytoplasm (Figure 5A) (Li et al., 2012). The expression of TBC1D6 markedly disrupted the Golgi localization of Rab26 but had no effect on the mutant Rab26Q123L. In contrast, the expression of TBC1D6 mutant RQ-AA, as well as other five TBC proteins, did not affect the Golgi localization of Rab26 (Figures 5A and 5B). These data suggest that TBC1D6 may enhance the transition of Rab26 from an active state to an inactive state in cell.

As Rab26 interaction with the ICL3 as well as full-length α_{2B}-AR was shown to highly depend on its activation (Li et al., 2012), these protein-protein interactions provide unique assays to measure Rab26 activation. The ICL3 of α_{2B}-AR was generated as fusion proteins and then incubated with the total cell lysates

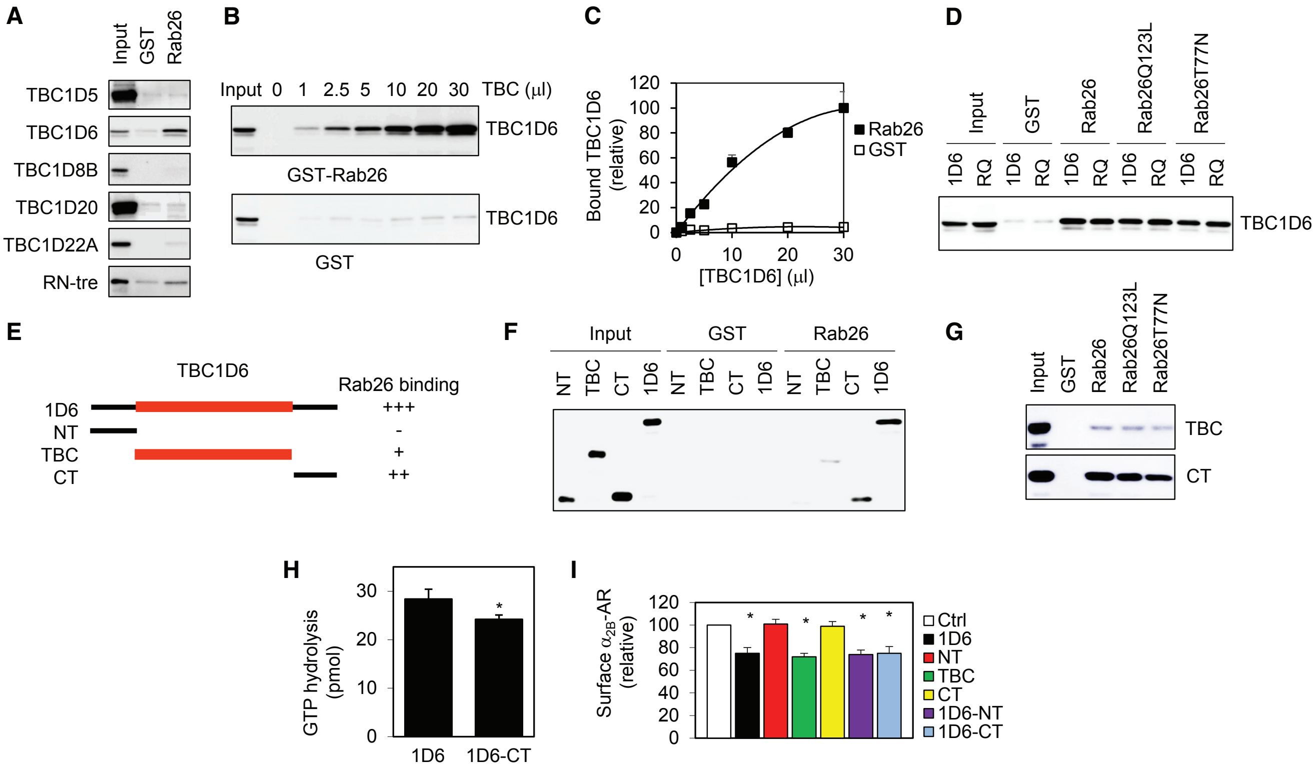

Figure 6. TBC1D6 Interaction with Rab26 and Identification of the Rab26-Binding Domains of TBC1D6

(A) Interaction of TBC1D6 with Rab26 in GST-Rab26 fusion protein pull-down assays.

(B) Dose dependent interaction of TBC1D6 with Rab26.

(C) Quantitative data shown in (B).

(D) Interaction of TBC1D6 and its catalytically inactive RQ-AA mutant with Rab26 and its GTP-bound (Q123L) and GDP-bound (T77N) mutants in GST fusion protein pull-down assays.

(E) Summary of Rab26 binding to full-length TBC1D6 and its N terminus (NT), TBC domain (TBC), and C terminus (CT) as shown in (F).

(F) A representative blot showing Rab26 interaction with different TBC1D6 domains.

(G) Interaction of the CT and the TBC domain of TBC1D6 with Rab26 and its GTP-bound (Q123L) and GDP-bound (T77N) mutants in GST fusion protein pull-down assays.

(H) Effect of TBC1D6 and its mutant lacking the C terminus (1D6-CT) on GTP hydrolysis by Rab26 measured in GAP assays.

(I) Effect of TBC1D6 domains on the cell surface transport of α_{2B}-AR. TBC1D6 and different domains were transiently expressed in HEK293 cells stably expressing α_{2B}-AR. The cell surface expression of α_{2B}-AR was measured by intact cell ligand binding.

Immunoblots shown are representatives of at least 3 experiments. Input represents 10% of total amount used in each reaction. The quantitative data shown are mean ± SE (n = 3–5). *$p < 0.05$ versus Ctrl.

prepared from cells expressing individual TBC proteins. As TBC1D20 has been well defined as a GAP for Rab1 and Rab2 (Haas et al., 2007; Ishida et al., 2012; Nachmias et al., 2012; Nevo-Yassaf et al., 2012; Sklan et al., 2007), it was not studied in this experiment. The expression of TBC1D6 strongly inhibited Rab26 interaction with the ICL3, whereas TBC1D5, TBC1D8B, TBC1D22A, and RN-tre had no significant effects (Figures 5C and 5D). In live cell bioluminescence resonance energy transfer (BRET) assays, the expression of TBC1D6, but not its inactive RQ-AA mutant, significantly reduced the BRET between α_{2B}-AR-Rluc and Rab26-venus (Figure 5E). These data demonstrate that TBC1D6 not only inactivates Rab26 but also attenuates its interaction with α_{2B}-AR.

The GAP assay was then utilized to directly measure the ability of TBC1D6 to accelerate the GTP hydrolysis by Rab GTPases. TBC1D6, but not its QR-AA mutant, markedly enhanced the GTP hydrolysis by Rab26, without affecting GTP hydrolysis by Rab1 (Figure 5F). The enhancement of Rab26-mediated GTP hydrolysis by TBC1D6 was in a time-dependent fashion (Figure 5G). These data demonstrate that TBC1D6 is a specific GAP for Rab26.

TBC1D6 Physically Associates with Rab26

Previous studies have demonstrated that TBC proteins may directly interact with their target Rabs (Dabbeekeh et al., 2007; Gallo et al., 2014; Haas et al., 2005; Itoh et al., 2006). We then sought to determine if Rab26 was able to physically associate with TBC1D6 in GST-Rab26 fusion protein pull-down assays. TBC1D6, but not TBC1D5, TBC1D18B, TBC1D20, TBC1D22A, and RN-tre, interacted with Rab26 (Figure 6A). The interaction was dependent upon TBC1D6 concentration (Figures 6B and 6C).

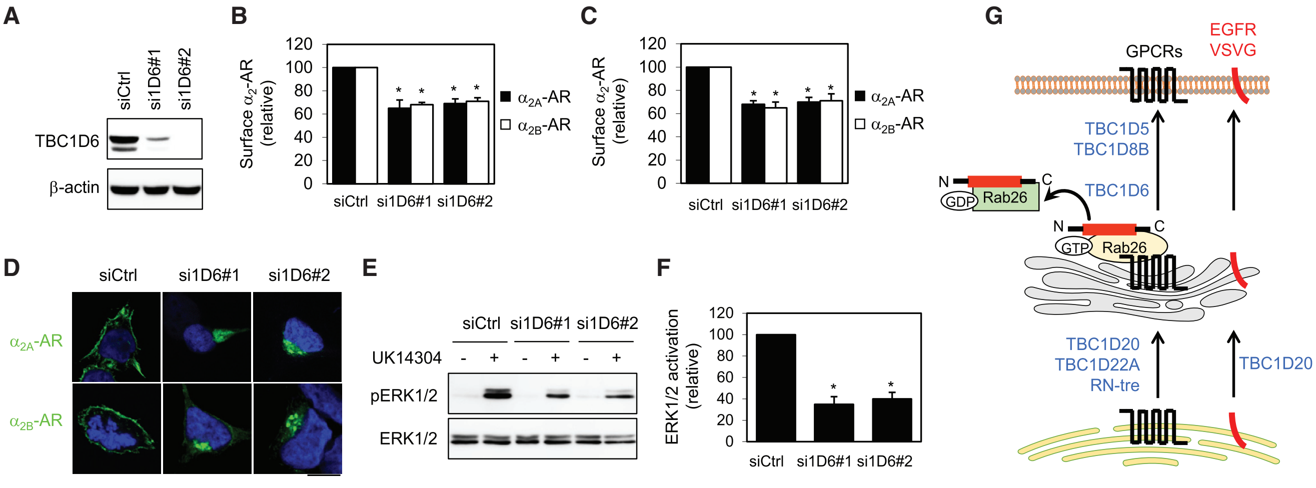

Figure 7. Effect of siRNA-Mediated Depletion of TBC1D6 on the Cell Surface Transport and Signaling of GPCRs

(A) Western blot analysis of siRNA-mediated knockdown of TBC1D6 in HEK293 cells.

(B) Effect of TBC1D6 siRNA on the surface expression of stably expressed α_{2A}-AR and α_{2B}-AR.

(C) Effect of TBC1D6 siRNA on the surface expression of inducibly expressed α_{2A}-AR and α_{2B}-AR.

(D) Effect of TBC1D6 siRNA on the subcellular localization of α_{2A}-AR and α_{2B}-AR. HEK293 cells were transfected with siRNA targeting TBC1D6 together with GFP-tagged α_{2A}-AR or α_{2B}-AR.

(E) Effect of TBC1D6 siRNA on α_{2B}-AR-mediated ERK1/2 activation in HEK293 cells stably expressing α_{2B}-AR.

(F) Quantitative data shown in (E).

(G) A model depicting the functions of six TBC proteins, as well as Rab26, in the forward ER-Golgi-plasma membrane transport of GPCRs and other plasma membranes (see text for detail).

The quantitative data are mean ± SE (n = 3–6). *$p < 0.05$ versus Ctrl. The images shown are representatives of 3 experiments. Scale bar, 10 μm.

To determine if the interaction between Rab26 and TBC1D6 was dependent on their activation, Rab26 and its GTP- and GDP-bound mutants were generated as GST fusion proteins and incubated with the total cell lysates expressing TBC1D6 or its inactive RQ-AA mutant. Rab26 and its mutants all strongly bound to TBC1D6 and RQ-AA mutant, and the binding affinities between wild type and mutated Rab26 and TBC1D6 are seemly similar (Figure 6D). These data suggest that interaction between Rab26 and TBC1D6 is independent of their activation statuses.

To identify the domains that mediate TBC1D6 interaction with Rab26, the N terminus, the TBC domain, and the C terminus of TBC1D6 were tagged with GFP, expressed in cells (Figure S5), and incubated with GST-Rab26 fusion proteins. Both the C terminus and the TBC domain, but not the N terminus, clearly interacted with Rab26. The C-terminal interaction with Rab26 was stronger than that of the TBC domain, but both were weaker than full-length TBC1D6 (Figures 6E and 6F). Similar to full-length TBC1D6, the interaction of TBC domain and C terminus with Rab26 is independent of Rab26 activation (Figure 6G). These data suggest that TBC1D6 has multiple Rab26-binding sites.

As the C terminus strongly bound to Rab26, we sought to determine its role in regulating TBC1D6 function. We first determined if the C terminus was required for the GAP activity of TBC1D6 toward Rab26. The hydrolysis of GTP by Rab26 was moderately but significantly weaker in the presence of TBC1D6 mutant lacking the C terminus (1D6-CT) than in the presence of full-length TBC1D6 as measured in *in vitro* GAP assays (Figure 6H). These data suggest that the C-terminal binding to Rab26 is able to enhance the GAP activity of TBC1D6. We then determined the role of different domains of TBC1D6 in GPCR transport. The expression of full-length TBC1D6, TBC domain alone, TBC-CT, and TBC1D6 mutant lacking the N-terminal portion (1D6-NT) similarly inhibited the cell surface transport of α_{2B}-AR, whereas the N and C termini had no effect (Figures 6I and S5). These data suggest that the TBC domain itself is sufficient to inhibit GPCR transport and that, although the C terminus is able to interact Rab26 and enhance the GAP activity of TBC1D6, it may not significantly contribute to the regulation of GPCR cell surface export at steady state.

Depletion of TBC1D6 Inhibits the Cell Surface Expression and Signaling of GPCRs

The function of TBC1D6 in regulating GPCR transport was further studied by siRNA-mediated depletion strategy. Due to the low expression level of endogenous TBC1D6 in HEK293 cells and the quality of TBC1D6 antibodies, we measured the ability of TBC1D6 siRNA to deplete transiently expressed GFP-tagged TBC1D6. Two siRNA targeting TBC1D6 markedly knocked down GFP-TBC1D6 by more than 75% when compared with cells transfected with control siRNA (Figure 7A). Interestingly, similar to its overexpression, siRNA-mediated knockdown of TBC1D6 significantly attenuated the cell surface numbers of stably expressed α_{2A}-AR and α_{2B}-AR in HEK293 cells (Figure 7B). siRNA targeting TBC1D6 also attenuated the cell surface expression of inducibly expressed α_{2A}-AR and α_{2B}-AR (Figure 7C). Both α_2-ARs were clearly arrested in the perinuclear region, most likely the Golgi, in cells expressing TBC1D6 siRNA (Figure 7D). Furthermore, expression of siRNA targeting TBC1D6 markedly inhibited α_{2B}-AR-mediated ERK1/2 activation by ~65% as compared to cells expressing control siRNA

(Figures 7E and 7F). These data suggest that the normal expression level of TBC1D6 is an essential, limiting factor for the anterograde transport of α_2-ARs.

Our preceding data demonstrate that overexpression and depletion of TBC1D6 produce an inhibitory effect on the cell surface transport of GPCRs, and TBC1D6 is likely a GAP for Rab26, suggesting that proper GDP/GTP cycling of Rab26 may be a crucial event for membrane trafficking of GPCRs. To further test this possibility, we determined the effect of transient expression of GTP-bound mutant Rab26Q123L and GDP-bound mutant Rab26T77N on the cell surface transport of several GPCRs. The expression of Rab26Q123L and Rab26T77N similarly inhibited the cell surface transport of α_{2A}-AR, α_{2B}-AR, β_2-AR, and AT1R (Figure S6A), but Rab26T77N did not affect the ER-Golgi-cell surface transport of VSVG (Figures S6B–S6D). As TBC1D20 was suggested to be a GAP for Rab33 (Fuchs et al., 2007; Pan et al., 2006), we determined if Rab33a and Rab33b were involved in the cell surface transport of two GPCRs, α_{2A}-AR and α_{2B}-AR. In contrast to Rab26, expression of Rab33a and Rab33b and their dominant-negative mutants had no significant effects on the cell surface expression of α_{2A}-AR and α_{2B}-AR (Figure S6E), suggesting that the function of TBC1D22A in GPCR trafficking is unlikely mediated via the inactivation of Rab33.

DISCUSSION

In this study, we have investigated the possible functions of RabGAPs in GPCR trafficking and identified six TBC proteins, namely TBC1D5, TBC1D6, TBC1D8B, TBC1D20, TBC1D22A, and RN-tre, which clearly regulate the anterograde cell surface transport of multiple GPCRs. This function of RabGAPs was first identified in a screening assay in which we measured the effect of almost all TBC proteins identified thus far on the cell surface expression of stably expressed α_{2B}-AR at steady state by radioligand binding of live cells, which was further confirmed by different cell systems in which the receptor was expressed transiently, inducibly, and endogenously. The function of these six TBC proteins in the cell surface transport of α_{2B}-AR was also supported by confocal microscopy to directly visualize the subcellular localization of the receptor and by receptor-mediated signaling measured as ERK1/2 activation. In addition to α_{2B}-AR, the cell surface expression of several other family A GPCRs are similarly affected by the six TBC proteins. These data demonstrate that TBC proteins are important regulators in GPCR trafficking. As the molecular mechanisms underlying the anterograde cell surface transport of GPCRs are poorly understood, these studies provide important insights into regulatory mechanisms of GPCR forward trafficking.

Our studies have also provided direct evidence implicating that TBC1D20, TBC1D22A, and RN-tre regulate the transport of GPCRs from the ER to the Golgi (Figure 7G). This became evident as enhanced expression of TBC1D20, TBC1D22A, and RN-tre induced extensive ER localization of all GPCRs studied, as well as the Golgi-localized α_{2B}-AR YS mutant. This is also strongly supported by fact that TBC1D20, TBC1D22A, and RN-tre significantly enhanced the formation of simple glycosylation that occurs in the ER. In contrast, TBC1D5, TBC1D6, and TBC1D8B are more likely involved in the transport of GPCRs at the level of the Golgi (Figure 7G), as their expression caused GPCR accumulation in the Golgi without disrupting the ER exit and N-linked glycosylation of the receptors. These data clearly demonstrate that distinct TBC proteins regulate the cell surface transport of GPCRs at discrete steps (Figure 7G).

Another important finding is that we have identified TBC1D6 as a specific GAP for Rab26. First, TBC1D6, but not its catalytically inactive RQ-AA mutant and other TBC proteins involved in GPCR transport, markedly inhibited the Golgi localization of Rab26 in cell, indicative of Rab26 inactivation. Second, TBC1D6 also markedly inhibited the activation-dependent interaction of Rab26 with the receptor. These data imply a possible molecular mechanism underlying the function of Rab26 and TBC1D6 in regulating GPCR cell surface trafficking, in which TBC1D6 inactivates Rab26 and inhibits Rab26 interaction with GPCRs and subsequent formation of transport machinery (Figure 7G). Third, the direct evidence indicating that TBC1D6 is a GAP for Rab26 is that it significantly enhanced the GTP hydrolysis by Rab26. Fourth, our previous and current studies reveal that both TBC1D6 and Rab26 regulate the post-Golgi transport of GPCRs, but not other plasma membrane proteins (such as VSVG and EGFR). Fifth, TBC1D6 and Rab26 interact and the interaction is independent of their activation statuses. Interestingly, both the TBC domain and the C terminus are able to interact with Rab26 albeit with different binding affinities and the C-terminal interaction with Rab26 enhances the GAP activity of TBC1D6. Sixth, it is worthwhile to point out that the same effects produced by overexpression and depletion of TBC1D6 suggest that proper GDP/GTP cycling of its target Rabs is crucial for the promotion of membrane trafficking of GPCRs. Indeed, both Rab26 GTP- and GDP-bound mutants prevent GPCRs to transport to the cell surface. Nevertheless, these data strongly suggest that Rab26 and TBC1D6 are a pair of Rab and GAP and provide an important mechanism underlying their function in regulating the post-Golgi traffic of GPCRs (Figure 7G).

The functions of TBC proteins in regulating GPCR transport are most likely specific. The most important evidence to support this is that catalytically inactive mutants of the six TBC proteins completely reversed their inhibitory effects on the cell surface transport of α_{2B}-AR, indicating that their effects on GPCR trafficking were caused by catalytic inactivation of Rab GTPases. Indeed, previous studies have shown that TBC1D20 is a GAP for Rab1 and Rab2 (Haas et al., 2007; Ishida et al., 2012; Nachmias et al., 2012; Nevo-Yassaf et al., 2012; Sklan et al., 2007) and RN-tre is a GAP for Rab43 (Fuchs et al., 2007; Haas et al., 2005, 2007). All three Rabs modulate the transport of multiple GPCRs between the ER and the Golgi (Dong and Wu, 2007; Li et al., 2017; Wu et al., 2003). We have shown here that TBC1D6 is a GAP for Rab26 that we have previously demonstrated to regulate the post-Golgi transport of GPCRs as discussed above (Li et al., 2012). Furthermore, the TBC domain of TBC1D6 is able to produce the maximal inhibitory effect on GPCR cell surface export. It should be pointed out that Rab7 (Borg Distefano et al., 2018; Seaman et al., 2009) and Rab33 (Fuchs et al., 2007; Pan et al., 2006) were suggested to be target Rabs for TBC1D5 and TBC1D22, but they unlikely regulate the forward cell surface transport of GPCRs as demonstrated in our previous (Li et al., 2017) and current studies, suggesting that the function

of TBD1D5 and TBC1D22A in GPCR trafficking may not be mediated via the inactivation of these Rabs. This notion is also supported by the fact that the expression of TBC1D25 and RUTBC1, other two well-characterized Rab33 GAPs (Itoh et al., 2011; Nottingham et al., 2011), did not affect the cell surface transport of α_{2B}-AR in our initial screening assay (Figure 1). In addition, TBC1D20 was the only TBC protein to inhibit the cell surface transport of EGFR and VSVG, consistent with a previous report showing that TBC1D20 blocks VSVG export from the ER (Haas et al., 2007). Altogether, these data strongly suggest the specific regulatory function of TBC proteins in GPCR forward trafficking. However, we cannot completely exclude the possibility that the inhibitory effect of TBC1D22A on GPCR transport was caused by the disruption of the Golgi structure as suggested in a previous study for Shiga toxin uptake (Fuchs et al., 2007). Nevertheless, these data suggest that specific Rabs and TBC proteins may form pairs to coordinate the ER-to-Golgi (e.g., Rab1-TBC1D20 and Rab43-RN-tre) and the Golgi-to-cell surface transport (e.g., Rab26-TBC1D6) of GPCRs (Figure 7G). Future studies to identify Rab substrates of TBC1D5, TBC1D8B, and TBC1D22A and evaluate their effect on the cell surface transport of GPCRs will further demonstrate specific function of Rab-TBC pairs in GPCR anterograde trafficking.

As the most structurally diverse superfamily of membrane proteins, GPCR trafficking, particularly their targeting to the cell surface, has been well described to play a crucial role in regulating the functionality of the receptors and its dysregulation directly causes many human diseases (Conn et al., 2007; Morello and Bichet, 2001). It is also interesting to note that recent studies have shown that TBC proteins may be associated with a variety of human diseases (Liegel et al., 2013; Vizoso et al., 2015), but the exact mechanisms remain largely unknown. Emerging evidence from the past decade studies suggest that the cell surface transport of GPCRs is tightly controlled by many factors, including specific export motifs embedded within the receptors (Dong et al., 2012; Dong and Wu, 2006; Duvernay et al., 2009; Zhang et al., 2011), receptor activity modifying proteins, chaperones, escort proteins, gatekeepers, protein kinases, and small GTPases (Chen et al., 2009; Colley et al., 1991; Doly et al., 2016; Dwyer et al., 1998; Ferreira et al., 1996; Hay and Pioszak, 2016; McLatchie et al., 1998; Sauvageau et al., 2014; Shiwarski et al., 2017; Tai et al., 1999). As shown in the current study, GPCR trafficking is tightly controlled by specific TBC proteins. Therefore, to thoroughly elucidate regulatory mechanisms of GPCR export to the functional destinations may provide an important foundation for the development of new therapeutic strategies by targeting GPCR export trafficking.

In summary, our present study has identified multiple TBC proteins as crucial regulators for GPCR trafficking, specifically anterograde cell surface transport, *en route* from the ER through the Golgi. These functions of TBC proteins are mediated through their GAP activity to inactivate target Rab GTPases and inhibit Rab interaction with the receptors. Overall, our data presented in this paper demonstrate the functional importance of the TBC family proteins in GPCR trafficking and provide important insights into regulation of GPCR targeting to the functional destination, as well as regulation of general vesicle-mediated membrane trafficking, by the TBC family proteins as Rab-specific GAPs.

STAR★METHODS

Detailed methods are provided in the online version of this paper and include the following:

- KEY RESOURCES TABLE
- LEAD CONTACT AND MATERIALS AVAILABILITY
- EXPERIMENTAL MODELS AND SUBJECT DETAILS
  - Cell culture
- METHOD DETAILS
  - Plasmid constructions
  - Transient transfection
  - Generation of stable and inducible cell lines expressing α_{2A}-AR and α_{2B}-AR
  - Depletion of TBC1D6 by siRNA
  - Radioligand binding of intact live cells
  - Flow cytometry
  - Fluorescence microscopy
  - Deglycosylation
  - Measurement of VSVG transport from the ER through the Golgi to the plasma membrane
  - Measurement of ERK1/2 activation
  - GST fusion protein pulldown assays
  - *In vitro* GAP assays
  - BRET assays
- STATISTICAL ANALYSIS

SUPPLEMENTAL INFORMATION

Supplemental Information can be found online at https://doi.org/10.1016/j.celrep.2019.05.033.

ACKNOWLEDGMENTS

We are grateful to Jeffrey L. Benovic (Thomas Jefferson University) and Alan V. Smrcka (University of Michigan) for sharing reagents. This work was supported by the NIH (R01GM118915 to G.W.). Z.D. is a recipient of VA Senior Research Career Scientist award.

AUTHOR CONTRIBUTIONS

Z.W., M.Z., C.L., and G.W. conceived and designed the experiments. Z.W., M.Z., C.L., W.H., Y.F., J.G., M.K., and G.W. performed the experiments. Z.W., M.Z., C.L., W.H., Z.D., G.H., M.K., and G.W. analyzed the results. M.F. provided materials. Z.W., Z.D., G.H., and G.W. wrote the manuscript with the input of all authors.

DECLARATION OF INTERESTS

The authors declare no competing interests.

Received: July 9, 2018
Revised: November 14, 2018
Accepted: May 9, 2019
Published: July 9, 2019

REFERENCES

Albert, S., Will, E., and Gallwitz, D. (1999). Identification of the catalytic domains and their functionally critical arginine residues of two yeast GTPase-activating proteins specific for Ypt/Rab transport GTPases. EMBO J. *18*, 5216–5225.

Barr, F., and Lambright, D.G. (2010). Rab GEFs and GAPs. Curr. Opin. Cell Biol. *22*, 461–470.

Bhosle, V.K., Rivera, J.C., Zhou, T.E., Omri, S., Sanchez, M., Hamel, D., Zhu, T., Rouget, R., Rabea, A.A., Hou, X., et al. (2016). Nuclear localization of platelet-activating factor receptor controls retinal neovascularization. Cell Discov. *2*, 16017.

Borg Distefano, M., Hofstad Haugen, L., Wang, Y., Perdreau-Dahl, H., Kjos, I., Jia, D., Morth, J.P., Neefjes, J., Bakke, O., and Progida, C. (2018). TBC1D5 controls the GTPase cycle of Rab7b. J. Cell Sci. *131*, jcs216630.

Chen, Y., Chen, C., Kotsikorou, E., Lynch, D.L., Reggio, P.H., and Liu-Chen, L.Y. (2009). GEC1-kappa opioid receptor binding involves hydrophobic interactions: GEC1 has chaperone-like effect. J. Biol. Chem. *284*, 1673–1685.

Colley, N.J., Baker, E.K., Stamnes, M.A., and Zuker, C.S. (1991). The cyclophilin homolog ninaA is required in the secretory pathway. Cell *67*, 255–263.

Conn, P.M., Ulloa-Aguirre, A., Ito, J., and Janovick, J.A. (2007). G protein-coupled receptor trafficking in health and disease: lessons learned to prepare for therapeutic mutant rescue in vivo. Pharmacol. Rev. *59*, 225–250.

Dabbeekeh, J.T., Faitar, S.L., Dufresne, C.P., and Cowell, J.K. (2007). The EVI5 TBC domain provides the GTPase-activating protein motif for RAB11. Oncogene *26*, 2804–2808.

Dale, L.B., Seachrist, J.L., Babwah, A.V., and Ferguson, S.S. (2004). Regulation of angiotensin II type 1A receptor intracellular retention, degradation, and recycling by Rab5, Rab7, and Rab11 GTPases. J. Biol. Chem. *279*, 13110–13118.

Davey, J.R., Humphrey, S.J., Junutula, J.R., Mishra, A.K., Lambright, D.G., James, D.E., and Stöckli, J. (2012). TBC1D13 is a RAB35 specific GAP that plays an important role in GLUT4 trafficking in adipocytes. Traffic *13*, 1429–1441.

DeGraff, J.L., Gagnon, A.W., Benovic, J.L., and Orsini, M.J. (1999). Role of arrestins in endocytosis and signaling of alpha2-adrenergic receptor subtypes. J. Biol. Chem. *274*, 11253–11259.

Doly, S., Shirvani, H., Gäta, G., Meye, F.J., Emerit, M.B., Enslen, H., Achour, L., Pardo-Lopez, L., Yang, S.K., Armand, V., et al. (2016). GABAB receptor cell-surface export is controlled by an endoplasmic reticulum gatekeeper. Mol. Psychiatry *21*, 480–490.

Dong, C., and Wu, G. (2006). Regulation of anterograde transport of alpha2-adrenergic receptors by the N termini at multiple intracellular compartments. J. Biol. Chem. *281*, 38543–38554.

Dong, C., and Wu, G. (2007). Regulation of anterograde transport of adrenergic and angiotensin II receptors by Rab2 and Rab6 GTPases. Cell. Signal. *19*, 2388–2399.

Dong, C., Yang, L., Zhang, X., Gu, H., Lam, M.L., Claycomb, W.C., Xia, H., and Wu, G. (2010). Rab8 interacts with distinct motifs in alpha2B- and beta2-adrenergic receptors and differentially modulates their transport. J. Biol. Chem. *285*, 20369–20380.

Dong, C., Li, C., and Wu, G. (2011). Regulation of α(2B)-adrenergic receptor-mediated extracellular signal-regulated kinase 1/2 (ERK1/2) activation by ADP-ribosylation factor 1. J. Biol. Chem. *286*, 43361–43369.

Dong, C., Nichols, C.D., Guo, J., Huang, W., Lambert, N.A., and Wu, G. (2012). A triple arg motif mediates α(2B)-adrenergic receptor interaction with Sec24C/D and export. Traffic *13*, 857–868.

Du, L.L., and Novick, P. (2001). Yeast rab GTPase-activating protein Gyp1p localizes to the Golgi apparatus and is a negative regulator of Ypt1p. Mol. Biol. Cell *12*, 1215–1226.

Duvernay, M.T., Dong, C., Zhang, X., Robitaille, M., Hébert, T.E., and Wu, G. (2009). A single conserved leucine residue on the first intracellular loop regulates ER export of G protein-coupled receptors. Traffic *10*, 552–566.

Duvernay, M.T., Wang, H., Dong, C., Guidry, J.J., Sackett, D.L., and Wu, G. (2011). Alpha2B-adrenergic receptor interaction with tubulin controls its transport from the endoplasmic reticulum to the cell surface. J. Biol. Chem. *286*, 14080–14089.

Dwyer, N.D., Troemel, E.R., Sengupta, P., and Bargmann, C.I. (1998). Odorant receptor localization to olfactory cilia is mediated by ODR-4, a novel membrane-associated protein. Cell *93*, 455–466.

Fan, G.H., Lapierre, L.A., Goldenring, J.R., and Richmond, A. (2003). Differential regulation of CXCR2 trafficking by Rab GTPases. Blood *101*, 2115–2124.

Ferreira, P.A., Nakayama, T.A., Pak, W.L., and Travis, G.H. (1996). Cyclophilin-related protein RanBP2 acts as chaperone for red/green opsin. Nature *383*, 637–640.

Filipeanu, C.M., Zhou, F., Fugetta, E.K., and Wu, G. (2006). Differential regulation of the cell-surface targeting and function of beta- and alpha1-adrenergic receptors by Rab1 GTPase in cardiac myocytes. Mol. Pharmacol. *69*, 1571–1578.

Frasa, M.A., Koessmeier, K.T., Ahmadian, M.R., and Braga, V.M. (2012). Illuminating the functional and structural repertoire of human TBC/RABGAPs. Nat. Rev. Mol. Cell Biol. *13*, 67–73.

Fuchs, E., Haas, A.K., Spooner, R.A., Yoshimura, S., Lord, J.M., and Barr, F.A. (2007). Specific Rab GTPase-activating proteins define the Shiga toxin and epidermal growth factor uptake pathways. J. Cell Biol. *177*, 1133–1143.

Fukuda, M. (2011). TBC proteins: GAPs for mammalian small GTPase Rab? Biosci. Rep. *31*, 159–168.

Gallo, L.I., Liao, Y., Ruiz, W.G., Clayton, D.R., Li, M., Liu, Y.J., Jiang, Y., Fukuda, M., Apodaca, G., and Yin, X.M. (2014). TBC1D9B functions as a GTPase-activating protein for Rab11a in polarized MDCK cells. Mol. Biol. Cell *25*, 3779–3797.

Goueli, B.S., Powell, M.B., Finger, E.C., and Pfeffer, S.R. (2012). TBC1D16 is a Rab4A GTPase activating protein that regulates receptor recycling and EGF receptor signaling. Proc. Natl. Acad. Sci. USA *109*, 15787–15792.

Grosshans, B.L., Ortiz, D., and Novick, P. (2006). Rabs and their effectors: achieving specificity in membrane traffic. Proc. Natl. Acad. Sci. USA *103*, 11821–11827.

Haas, A.K., Fuchs, E., Kopajtich, R., and Barr, F.A. (2005). A GTPase-activating protein controls Rab5 function in endocytic trafficking. Nat. Cell Biol. *7*, 887–893.

Haas, A.K., Yoshimura, S., Stephens, D.J., Preisinger, C., Fuchs, E., and Barr, F.A. (2007). Analysis of GTPase-activating proteins: Rab1 and Rab43 are key Rabs required to maintain a functional Golgi complex in human cells. J. Cell Sci. *120*, 2997–3010.

Hanyaloglu, A.C., and von Zastrow, M. (2008). Regulation of GPCRs by endocytic membrane trafficking and its potential implications. Annu. Rev. Pharmacol. Toxicol. *48*, 537–568.

Hauser, A.S., Attwood, M.M., Rask-Andersen, M., Schiöth, H.B., and Gloriam, D.E. (2017). Trends in GPCR drug discovery: new agents, targets and indications. Nat. Rev. Drug Discov. *16*, 829–842.

Hay, D.L., and Pioszak, A.A. (2016). Receptor Activity-Modifying Proteins (RAMPs): New Insights and Roles. Annu. Rev. Pharmacol. Toxicol. *56*, 469–487.

Hilger, D., Masureel, M., and Kobilka, B.K. (2018). Structure and dynamics of GPCR signaling complexes. Nat. Struct. Mol. Biol. *25*, 4–12.

Hsu, C., Morohashi, Y., Yoshimura, S., Manrique-Hoyos, N., Jung, S., Lauterbach, M.A., Bakhti, M., Grønborg, M., Möbius, W., Rhee, J., et al. (2010). Regulation of exosome secretion by Rab35 and its GTPase-activating proteins TBC1D10A-C. J. Cell Biol. *189*, 223–232.

Ishibashi, K., Kanno, E., Itoh, T., and Fukuda, M. (2009). Identification and characterization of a novel Tre-2/Bub2/Cdc16 (TBC) protein that possesses Rab3A-GAP activity. Genes Cells *14*, 41–52.

Ishida, M., Ohbayashi, N., Maruta, Y., Ebata, Y., and Fukuda, M. (2012). Functional involvement of Rab1A in microtubule-dependent anterograde melanosome transport in melanocytes. J. Cell Sci. *125*, 5177–5187.

Itoh, T., and Fukuda, M. (2006). Identification of EPI64 as a GTPase-activating protein specific for Rab27A. J. Biol. Chem. *281*, 31823–31831.

Itoh, T., Satoh, M., Kanno, E., and Fukuda, M. (2006). Screening for target Rabs of TBC (Tre-2/Bub2/Cdc16) domain-containing proteins based on their Rab-binding activity. Genes Cells *11*, 1023–1037.

Itoh, T., Kanno, E., Uemura, T., Waguri, S., and Fukuda, M. (2011). OATL1, a novel autophagosome-resident Rab33B-GAP, regulates autophagosomal maturation. J. Cell Biol. *192*, 839–853.

Li, C., Fan, Y., Lan, T.H., Lambert, N.A., and Wu, G. (2012). Rab26 modulates the cell surface transport of α2-adrenergic receptors from the Golgi. J. Biol. Chem. *287*, 42784–42794.

Li, C., Wei, Z., Fan, Y., Huang, W., Su, Y., Li, H., Dong, Z., Fukuda, M., Khater, M., and Wu, G. (2017). The GTPase Rab43 controls the anterograde ER-Golgi trafficking and sorting of GPCRs. Cell Rep. *21*, 1089–1101.

Liegel, R.P., Handley, M.T., Ronchetti, A., Brown, S., Langemeyer, L., Linford, A., Chang, B., Morris-Rosendahl, D.J., Carpanini, S., Posmyk, R., et al. (2013). Loss-of-function mutations in TBC1D20 cause cataracts and male infertility in blind sterile mice and Warburg micro syndrome in humans. Am. J. Hum. Genet. *93*, 1001–1014.

Marchese, A., Paing, M.M., Temple, B.R., and Trejo, J. (2008). G protein-coupled receptor sorting to endosomes and lysosomes. Annu. Rev. Pharmacol. Toxicol. *48*, 601–629.

McLatchie, L.M., Fraser, N.J., Main, M.J., Wise, A., Brown, J., Thompson, N., Solari, R., Lee, M.G., and Foord, S.M. (1998). RAMPs regulate the transport and ligand specificity of the calcitonin-receptor-like receptor. Nature *393*, 333–339.

Morello, J.P., and Bichet, D.G. (2001). Nephrogenic diabetes insipidus. Annu. Rev. Physiol. *63*, 607–630.

Nachmias, D., Sklan, E.H., Ehrlich, M., and Bacharach, E. (2012). Human immunodeficiency virus type 1 envelope proteins traffic toward virion assembly sites via a TBC1D20/Rab1-regulated pathway. Retrovirology *9*, 7.

Nevo-Yassaf, I., Yaffe, Y., Asher, M., Ravid, O., Eizenberg, S., Henis, Y.I., Nahmias, Y., Hirschberg, K., and Sklan, E.H. (2012). Role for TBC1D20 and Rab1 in hepatitis C virus replication via interaction with lipid droplet-bound nonstructural protein 5A. J. Virol. *86*, 6491–6502.

Nottingham, R.M., Ganley, I.G., Barr, F.A., Lambright, D.G., and Pfeffer, S.R. (2011). RUTBC1 protein, a Rab9A effector that activates GTP hydrolysis by Rab32 and Rab33B proteins. J. Biol. Chem. *286*, 33213–33222.

Pan, X., Eathiraj, S., Munson, M., and Lambright, D.G. (2006). TBC-domain GAPs for Rab GTPases accelerate GTP hydrolysis by a dual-finger mechanism. Nature *442*, 303–306.

Peralta, E.R., Martin, B.C., and Edinger, A.L. (2010). Differential effects of TBC1D15 and mammalian Vps39 on Rab7 activation state, lysosomal morphology, and growth factor dependence. J. Biol. Chem. *285*, 16814–16821.

Pfeffer, S.R. (2017). Rab GTPases: master regulators that establish the secretory and endocytic pathways. Mol. Biol. Cell *28*, 712–715.

Pierce, K.L., Premont, R.T., and Lefkowitz, R.J. (2002). Seven-transmembrane receptors. Nat. Rev. Mol. Cell Biol. *3*, 639–650.

Presley, J.F., Cole, N.B., Schroer, T.A., Hirschberg, K., Zaal, K.J.M., and Lippincott-Schwartz, J. (1997). ER-to-Golgi transport visualized in living cells. Nature *389*, 81–85.

Sauvageau, E., Rochdi, M.D., Oueslati, M., Hamdan, F.F., Percherancier, Y., Simpson, J.C., Pepperkok, R., and Bouvier, M. (2014). CNIH4 interacts with newly synthesized GPCR and controls their export from the endoplasmic reticulum. Traffic *15*, 383–400.

Seaman, M.N., Harbour, M.E., Tattersall, D., Read, E., and Bright, N. (2009). Membrane recruitment of the cargo-selective retromer subcomplex is catalysed by the small GTPase Rab7 and inhibited by the Rab-GAP TBC1D5. J. Cell Sci. *122*, 2371–2382.

Shiwarski, D.J., Darr, M., Telmer, C.A., Bruchez, M.P., and Puthenveedu, M.A. (2017). PI3K class II α regulates δ-opioid receptor export from the *trans*-Golgi network. Mol. Biol. Cell *28*, 2202–2219.

Sklan, E.H., Serrano, R.L., Einav, S., Pfeffer, S.R., Lambright, D.G., and Glenn, J.S. (2007). TBC1D20 is a Rab1 GTPase-activating protein that mediates hepatitis C virus replication. J. Biol. Chem. *282*, 36354–36361.

Stenmark, H. (2009). Rab GTPases as coordinators of vesicle traffic. Nat. Rev. Mol. Cell Biol. *10*, 513–525.

Surve, C.R., Lehmann, D., and Smrcka, A.V. (2014). A chemical biology approach demonstrates G protein βγ subunits are sufficient to mediate directional neutrophil chemotaxis. J. Biol. Chem. *289*, 17791–17801.

Tai, A.W., Chuang, J.Z., Bode, C., Wolfrum, U., and Sung, C.H. (1999). Rhodopsin's carboxy-terminal cytoplasmic tail acts as a membrane receptor for cytoplasmic dynein by binding to the dynein light chain Tctex-1. Cell *97*, 877–887.

Vizoso, M., Ferreira, H.J., Lopez-Serra, P., Carmona, F.J., Martínez-Cardús, A., Girotti, M.R., Villanueva, A., Guil, S., Moutinho, C., Liz, J., et al. (2015). Epigenetic activation of a cryptic TBC1D16 transcript enhances melanoma progression by targeting EGFR. Nat. Med. *21*, 741–750.

Wang, G., and Wu, G. (2012). Small GTPase regulation of GPCR anterograde trafficking. Trends Pharmacol. Sci. *33*, 28–34.

Wu, G., Zhao, G., and He, Y. (2003). Distinct pathways for the trafficking of angiotensin II and adrenergic receptors from the endoplasmic reticulum to the cell surface: Rab1-independent transport of a G protein-coupled receptor. J. Biol. Chem. *278*, 47062–47069.

Yoshimura, S., Egerer, J., Fuchs, E., Haas, A.K., and Barr, F.A. (2007). Functional dissection of Rab GTPases involved in primary cilium formation. J. Cell Biol. *178*, 363–369.

Zhang, X., Dong, C., Wu, Q.J., Balch, W.E., and Wu, G. (2011). Di-acidic motifs in the membrane-distal C termini modulate the transport of angiotensin II receptors from the endoplasmic reticulum to the cell surface. J. Biol. Chem. *286*, 20525–20535.

Zhang, M., Davis, J.E., Li, C., Gao, J., Huang, W., Lambert, N.A., Terry, A.V., Jr., and Wu, G. (2016a). GGA3 Interacts with a G Protein-Coupled Receptor and Modulates Its Cell Surface Export. Mol. Cell. Biol. *36*, 1152–1163.

Zhang, M., Huang, W., Gao, J., Terry, A.V., and Wu, G. (2016b). Regulation of α2B-Adrenergic Receptor Cell Surface Transport by GGA1 and GGA2. Sci. Rep. *6*, 37921.

STAR★METHODS

KEY RESOURCES TABLE

REAGENT or RESOURCE	SOURCE	IDENTIFIER
Antibodies		
Mouse monoclonal anti-GFP (clone B-2)	Santa Cruz Biotechnology	sc-9996
Rabbit polyclonal anti-calregulin (clone H-170)	Santa Cruz Biotechnology	sc-11398
Mouse monoclonal anti-β-actin (clone C4)	Santa Cruz Biotechnology	sc-47778
Mouse monoclonal anti-p-ERK (clone E-4)	Santa Cruz Biotechnology	sc-7383
Mouse monoclonal anti-GM130 (clone 35)	BD Biosciences	610823
Rabbit polyclonal anti-p44/42 MAPK (Erk1/2)	Cell Signaling Technology	9102
Mouse monoclonal anti-HA (clone 12CA5)	Roche	11583816001
Rat monoclonal anti-HA-fluorescein (clone 3F10)	Roche	11988506001
Goat anti-mouse IgG (H+L), Alexa Fluor 647	Thermo Fisher Scientific	A-21235
Goat anti-rabbit IgG (H+L), Alexa Fluor 647	Thermo Fisher Scientific	A-21244
Chemicals, Peptides, and Recombinant Proteins		
UK14304	Sigma-Aldrich	U104
NSC12155	Sigma-Aldrich	S6951
Activated charcoal	Sigma-Aldrich	242276
IPTG	Sigma-Aldrich	I5502
Doxycycline hyclate	Sigma-Aldrich	D9891
$[^3H]$-RX821002	Perkin-Elmer	NET1153250UC
$[^3H]$-CGP12177	Perkin-Elmer	NET1061250UC
$[\gamma\text{-}^{32}P]$-GTP	Perkin-Elmer	BLU004Z250UC
Lipofectamine 2000	Thermo Fisher Scientific	11668019
GTP	Thermo Fisher Scientific	18332015
EGF	Thermo Fisher Scientific	PHG0311
MagneGST glutathione particles	Promega	V8611
Dulbecco's modified eagles medium	GE Healthcare Life Sciences	SH30243.01HI
Fetal bovine serum	GE Healthcare Life Sciences	SH30396.03HI
Endo H	New England Biolabs	P0702L
PNGase F	New England Biolabs	P0708S
Experimental Models: Cell Lines		
HEK293	ATCC	CRL-1573
HT-29	ATCC	HTB-38
MCF-7	ATCC	HTB-22
Oligonucleotides		
Human TBC1D6 Stealth siRNA #1	Thermo Fisher Scientific	1299001 ID:HSS128833
Human TBC1D6 Stealth siRNA #2	Thermo Fisher Scientific	1299001 ID:HSS128835
Stealth siRNA negative control, Med GC	Thermo Fisher Scientific	12935300
Primers (Table S1)	This paper	N/A
Recombinant DNA		
α_{2A}-AR-GFP	Li et al., 2017	N/A
HA-α_{2A}-AR	Li et al., 2017	N/A
α_{2B}-AR YS-GFP	Dong and Wu, 2006	N/A
α_{2B}-AR-GFP	Wu et al., 2003	N/A
HA-α_{2B}-AR	Li et al., 2017	N/A
α_{2B}-AR-Venus	Li et al., 2012	N/A
AT1R-GFP	Li et al., 2017	N/A

(Continued on next page)

Continued

REAGENT or RESOURCE	SOURCE	IDENTIFIER
β_2-AR-GFP	Wu et al., 2003	N/A
GFP-TBC	Itoh et al., 2011	N/A
DsRed-TBC	This paper	N/A
GFP-Rab3GAP	Itoh et al., 2011	N/A
EGFR-GFP	Li et al., 2017	N/A
VSVGtsO45-GFP	Addgene	11912
GST-Rab1	This paper	N/A
GST-Rab26	This paper	N/A
GST-TBC1D6	This paper	N/A
GST-α_{2B}-AR ICL3	Wu et al., 2003	N/A
GST-α_{2A}-AR ICL3	Zhang et al., 2016a	N/A
GST-AT1R CT	Duvernay et al., 2011	N/A
Rab26-Rluc8	Li et al., 2017	N/A
Software and Algorithms		
ImageJ	NIH	https://imagej.nih.gov/ij/

LEAD CONTACT AND MATERIALS AVAILABILITY

Further information and requests for resources and reagents should be directed to and will be fulfilled by the Lead Contact, Guangyu Wu (guwu@augusta.edu).

EXPERIMENTAL MODELS AND SUBJECT DETAILS

Cell culture

HEK293 cells, HT-29 cells which express endogenous α_{2A}-AR, and MCF-7 cells which express endogenous α_{2B}-AR were cultured AT 37°C HT-29 human colon cancer cells expressing endogenously α_{2A}-AR were cultured in Dulbecco's modified Eagle's medium (DMEM) with 10% fetal bovine serum, 100 units/ml penicillin and 100 μg/ml streptomycin.

METHOD DETAILS

Plasmid constructions

GPCRs tagged with GFP at their C-termini or HA at their N-termini were generated as described previously (Dong et al., 2010, 2012; Li et al., 2012). TBC proteins and Rab3GAP in the pEGFP-C1 vector were generated as described (Ishibashi et al., 2009; Itoh et al., 2011). TBC proteins were also cloned into the pDsRed-monomer-C1 vector at the BamH1 and XhoI (TBC1D5), EcoR1 and XhoI (TBC1D20), or EcoRI and BamHI (TBC1D6, TBC1D8B, TBC1D22A and RN-tre) restriction sites using primers (Table S1). The GST-Rab26 and GST-TBC1D6 constructs were generated in the pGEX-4T-1 vector using the primers (Table S1). TBC and Rab26 mutants were generated by using QuikChange site-directed mutagenesis. All constructs used in the present study were verified by nucleotide sequence analysis.

Transient transfection

Transient transfection of cells were carried out by using Lipofectamine 2000 as described previously (Wu et al., 2003). Transfection efficiency of the cells was estimated to be greater than 75% based on microscopy detecting the fluorescence of tagged proteins.

Generation of stable and inducible cell lines expressing α_{2A}-AR and α_{2B}-AR

HEK293 cell lines stably expressing HA-α_{2B}-AR were generated as described previously (Li et al., 2012). Stable HEK293 cell lines inducibly expressing HA-α_{2A}-AR and HA-α_{2B}-AR was generated using the Tet-On 3G Tetracycline Inducible Gene Expression System (Clontech Laboratories, Inc.) as described previously (Zhang et al., 2016a). The cell lines expressing 8.3 × 10^5 α_{2A}-AR and 8.5 × 10^5 α_{2B}-AR per cell were utilized in the current study.

Depletion of TBC1D6 by siRNA

Two Stealth RNAi duplexes (siRNA) targeting to human TBC1D6 (NCBI: AB449887; # 1, 5′-CCUGUACAAUGUGCUGCUGGCAUAU-3′; # 2, 5′-UCGUGAUGGAGUGUCACACGUUUAU-3′) and a negative control med GC duplex were purchased from Invitrogen. siRNAs

targeting to Rab26 and GGA3 were described (Li et al., 2012; Zhang et al., 2016a). siRNA-mediated knockdown of TBC1D6, Rab26 and GGA3 was carried out as described previously (Wu et al., 2003). All of the experiments were performed at 72 h after the first siRNA transfection.

Radioligand binding of intact live cells

The cell surface expression of α_2-AR and β_2-AR was measured by ligand binding of intact live cells as described previously (Dong et al., 2012; Li et al., 2012) using [^{3}H]-RX821002 and [^{3}H]-CGP12177, respectively. For measurement of endogenous α_2-AR, HT-29 and MCF-7 cells were cultured on 6-well dishes and transfected with TBC1D6 constructs for 36-48 h or siRNA targeting TBC1D6 for 72 h. All radioligand binding assays were performed in triplicate.

Flow cytometry

For measurement of receptor expression at the cell surface, HEK293 cells transfected with HA-tagged receptors were suspended in PBS containing 1% fetal bovine serum and incubated with high affinity anti-HA-fluorescein (3F10) at 2 μg/ml for 30 min at 4°C. For measurement of total receptor expression, HEK293 cells were permeabilized with 0.2% Triton X-100 in PBS for 5 min before incubation with anti-HA antibodies. The fluorescence was analyzed on a flow cytometer (Dickinson FACSCalibur) as described (Wu et al., 2003).

Fluorescence microscopy

Cells were fixed with 4% paraformaldehyde for 15 min, permeabilized with 0.25% Triton X-100 for 5 min, and blocked with normal donkey serum for 30 min. The cells were sequentially stained with primary antibodies (1:200 dilution for GM130 and 1:50 dilution for calregulin) and Alexa fluor-conjugated secondary antibodies (1:500 dilution). Images were captured using a Zeiss LSM780 confocal microscope equipped with a 63 × objective.

Deglycosylation

Deglycosylation of GPCRs by treatments with Endo H and PNGase F enzymes was performed following the manufacturer's instructions with modifications. Briefly, cell lysates prepared from HEK293 cells transfected with individual receptors with or without TBC proteins were incubated in denaturing buffer containing 0.5% SDS and 40 mM DTT for 30 min at 37°C and the denatured proteins were digested with 1-2 μL enzymes in GlycoBuffer for 1 h at 37°C.

Measurement of VSVG transport from the ER through the Golgi to the plasma membrane

The transport of VSVG was measured by using its temperature sensitive mutant (VSVGtsO45) which was misfolded and retained within the ER at the restrictive temperature 40°C and correctly delivered to the Golgi and plasma membrane at the permissive temperature 32°C (Presley et al., 1997). HEK293 cells grown on coverslips in 6-well dishes were transfected with 0.25 μg of VSVGtsO45-GFP constructs together with dsRed-tagged TBC proteins. The cells were cultured for 24 h at 40°C to induce the accumulation of VSVG in the ER and then transferred to 32°C for up to 180 min to allow VSVG to transport to the Golgi and the cell surface. After the cells were fixed VSVG expression at the Golgi and the plasma membrane were quantified by fluorescent intensity using the ImageJ software. A total of 25-30 cells were quantified in three experiments.

Measurement of ERK1/2 activation

HEK293 cells stably expressing α_{2B}-AR were cultured on 6-well dishes and transfected with TBC1D6 or siRNA targeting TBC1D6. The cells were starved for 3 h before stimulation with UK14304 at 1 μM for 5 min. Stimulation was terminated by the addition of 200 μL of ice-cold cell lysis buffer. ERK1/2 activation was determined by measuring ERK1/2 phosphorylation by immunoblotting.

GST fusion protein pulldown assays

GST fusion protein pulldown assays were carried out by using the MagneGST pulldown system as described previously (Li et al., 2012; Zhang et al., 2016a). Briefly, GST fusion proteins were incubated with total HEK293 cell homogenates in 500 μL of binding buffer containing 20 mM Tris-HCl, pH 7.5, 140 m NaCl, 1% Nonidet P-40 for 4 h at 4°C. The bound proteins were solubilized and detected by immunoblotting.

In vitro GAP assays

The GAP assays were performed as described previously (Fuchs et al., 2007; Haas et al., 2005) with modifications. Briefly, GST-Rab26 or GST-Rab1 (50 pmol) were incubated with 1 μl of [γ-^{32}P]GTP in 50 μl buffer (50 mM HEPES-NaOH, pH 6.8, 1 mM DTT, 1 mg/ml bovine serum albumin, 1 mM EDTA and 0.5 mM of an equal mixture of GTP and $MgCl_2$) for 15 min at 30°C. GST-TBC1D6 or its mutants (4.6 pmol) were then added to the reaction mixture and incubated at 30°C for 60 min. At each time point, 5 μl of the reaction was removed and mixed with 795 μl of activated charcoal. After centrifugation, 400 μl of the cleared supernatant was counted and the ^{32}Pi release was determined by the radioactivity difference at different time points.

BRET assays

The live cell-based BRET assay to measure the possible interaction between α_{2B}-AR and Rab26 was carried out as described previously (Li et al., 2012, 2017). HEK293 cells were cultured on 6-well dishes and transfected with 0.1 μg of Rab-Rluc8 and 1.5 μg of α_{2B}-AR-Venus plus 1.0 μg of the pCMV-myc vector or myc-TBC for 36 h.

STATISTICAL ANALYSIS

All data were calculated and presented as mean ± SE. The exact value of n is given in each figure legend, where n represents the number of independent repeats. Statistical analysis was performed using unpaired Student's t test. $p < 0.05$ was considered as statistically significant.

Cell Reports, Volume *28*

Supplemental Information

Specific TBC Domain-Containing Proteins Control the ER-Golgi-Plasma Membrane Trafficking of GPCRs

Zhe Wei, Maoxiang Zhang, Chunman Li, Wei Huang, Yi Fan, Jianhui Guo, Mostafa Khater, Mitsunori Fukuda, Zheng Dong, Gang Hu, and Guangyu Wu

Figure S1

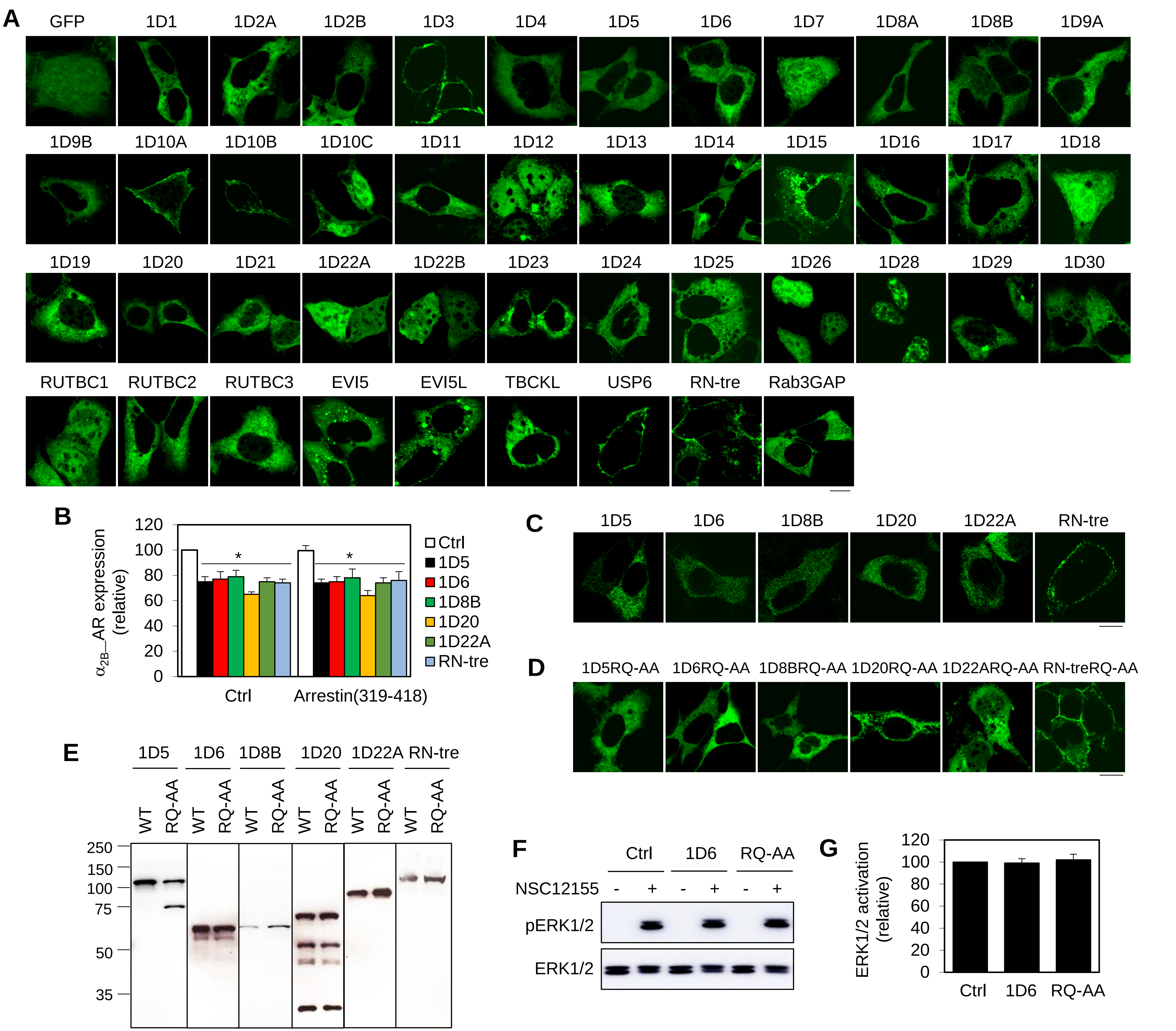
A
GFP
1D1
1D2A
1D2B
1D3
1D4
1D5
1D6
1D7
1D8A
1D8B
1D9A
1D9B
1D10A
1D10B
1D10C
1D11
1D12
1D13
1D14
1D15
1D16
1D17
1D18
1D19
1D20
1D21
1D22A
1D22B
1D23
1D24
1D25
1D26
1D28
1D29
1D30
RUTBC1
RUTBC2
RUTBC3
EVI5
EVI5L
TBCKL
USP6
RN-tre
Rab3GAP
B
α_{2B}-AR expression (relative)
120
100
80
60
40
20
0
*
*
Ctrl
Arrestin(319-418)
Ctrl
1D5
1D6
1D8B
1D20
1D22A
RN-tre
C
1D5
1D6
1D8B
1D20
1D22A
RN-tre
D
1D5RQ-AA
1D6RQ-AA
1D8BRQ-AA
1D20RQ-AA
1D22ARQ-AA
RN-treRQ-AA
E
1D5
1D6
1D8B
1D20
1D22A
RN-tre
WT
RQ-AA
250
150
100
75
50
35
F
Ctrl
1D6
RQ-AA
NSC12155
- + - + - +
pERK1/2
ERK1/2
G
ERK1/2 activation (relative)
120
100
80
60
40
20
0
Ctrl
1D6
RQ-AA

Figure S1. Screening for TBC proteins involved in the surface transport of α_{2B}-AR. Related to Figure 1.

(A) Expression and subcellular localization of TBC proteins. Individual GFP-tagged TBC proteins were transiently expressed in HEK293 cells and their subcellular localization was revealed by confocal microscopy.

(B) Effect of dominant negative arrestin-3 mutant on the cell surface expression of α_{2B}-AR. HEK293 cells were transfected with α_{2B}-AR and individual TBC proteins together with control vector or dominant negative arrestin-3(201-490) for 36 h and then the cell surface α_{2B}-AR expression was determined by intact cell ligand binding

(C) Expression of TBC proteins in MCF7 cells.

(D) Expression of TBC mutants in HEK239 cells.

(E) Western blots showing the expression of TBC proteins and their RQ-AA mutants.

(F) Effect of TBC1D6 on the activation of ERK1/2 by NSC12155. HEK293 cells were transfected with the pEGFP-C1 vector (Ctrl), GFP-TBC1D6 or GFP-TBC1D6 RQ-AA mutant and then stimulated with NSC12155 at a concentration of 10 μM for 5 min. The activation of ERK1/2 was measured by immunoblotting.

(G) Quantitative data shown in F

The data shown in B and G are mean ± SE (n=3). *$p<0.05$ versus Ctrl. The images are representative of at least 3 experiments. Scale bars, 10 μm.

Figure S2

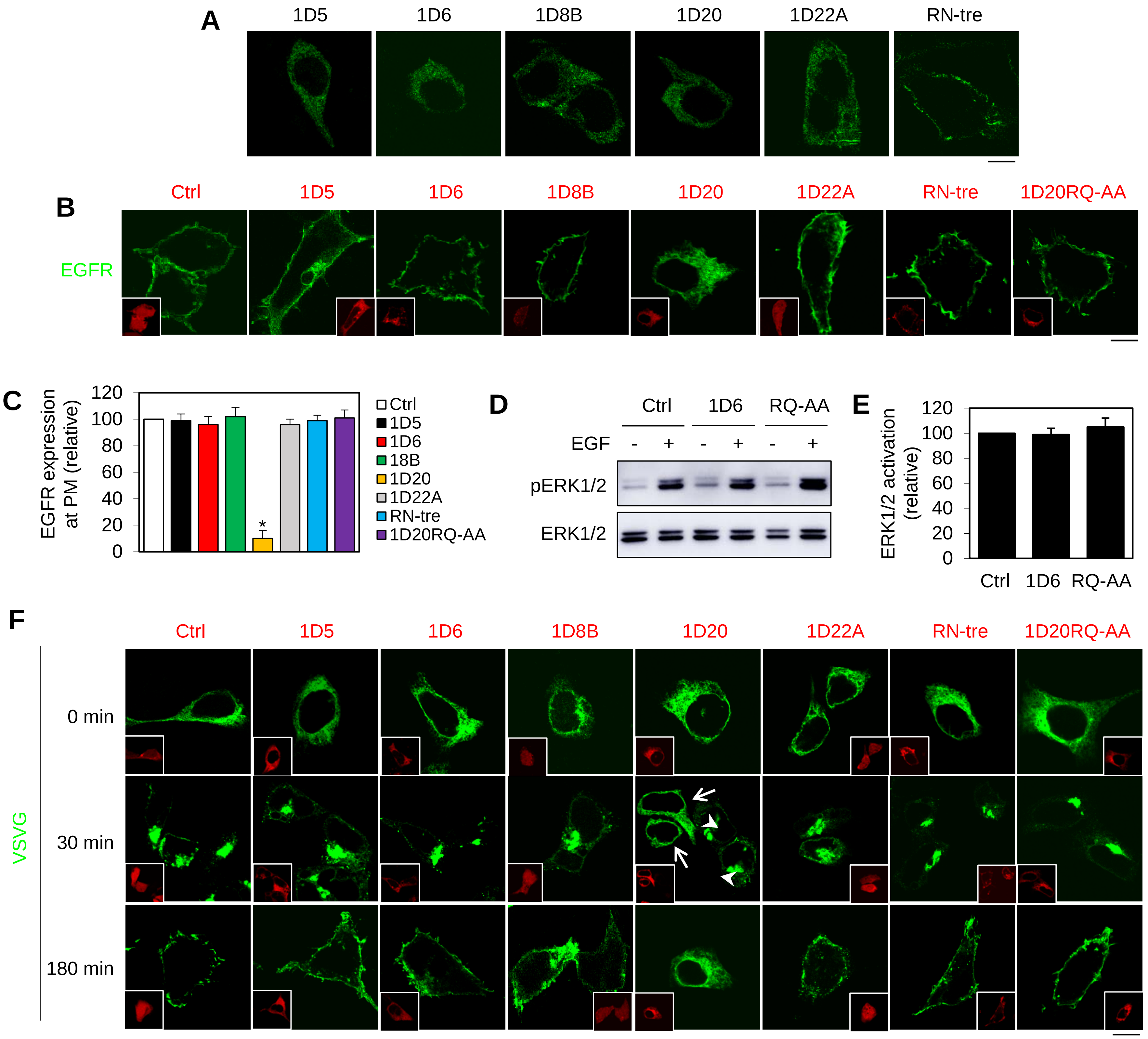

A
1D5
1D6
1D8B
1D20
1D22A
RN-tre
B
Ctrl
1D5
1D6
1D8B
1D20
1D22A
RN-tre
1D20RQ-AA
EGFR
C
EGFR expression at PM (relative)
120
100
80
60
40
20
0
*
Ctrl
1D5
1D6
18B
1D20
1D22A
RN-tre
1D20RQ-AA
D
Ctrl
1D6
RQ-AA
EGF
- + - + - +
pERK1/2
ERK1/2
E
ERK1/2 activation (relative)
120
100
80
60
40
20
0
Ctrl
1D6
RQ-AA
F
Ctrl
1D5
1D6
1D8B
1D20
1D22A
RN-tre
1D20RQ-AA
VSVG
0 min
30 min
180 min

Figure S2. Expression of TBC proteins and their effects on the cell surface transport of EGFR and VSVG. Related to Figure 3.

(A) Expression of TBC proteins in HT29 cells. The images are representatives of at least 3 experiments.

(B) Effect of TBC proteins on the cell surface expression of EGFR.

(C) Quantitative data shown in B. The cell surface expression of EGFR was determined by measuring the fluorescent intensity at the cell surface. The data shown are relative to the total expression of EGFR with a total of 25-30 cells quantified.

(D) Effect of TBC1D6 on the activation of ERK1/2 by EGF. HEK293 cells were transfected with the pEGF-C1 vector, GFP-TB1D6 or GFP-TBC1D6 RQ-AA mutant and then stimulated with EGF at 10 mg/ml for 10 min. ERK1/2 activation was determined by immunoblotting.

(E) Quantitative data shown in D.

(F) Effect of TBC proteins on the ER-Golgi-plasma membrane transport of VSVG. HEK293 cells were transfected with VSVGtsO45-GFP together with dsRed-C1 (Ctrl) or individual dsRed-TBC proteins. The cells were cultured at 40˚C for 24 h (0 min) and then shifted to 32˚C for different periods of time. The images shown are representative VSVG expression at 0, 30 and 180 min. Arrows indicate VSVG accumulation in TBC1D20-expressing cells and arrow heads indicate VSVG transport to the Golgi in cells without TBC1D20 transfection.

The data shown in C and E are mean ± SE (n=3). *$p < 0.05$ versus Ctrl. Scale bars, 10 μm.

Figure S3

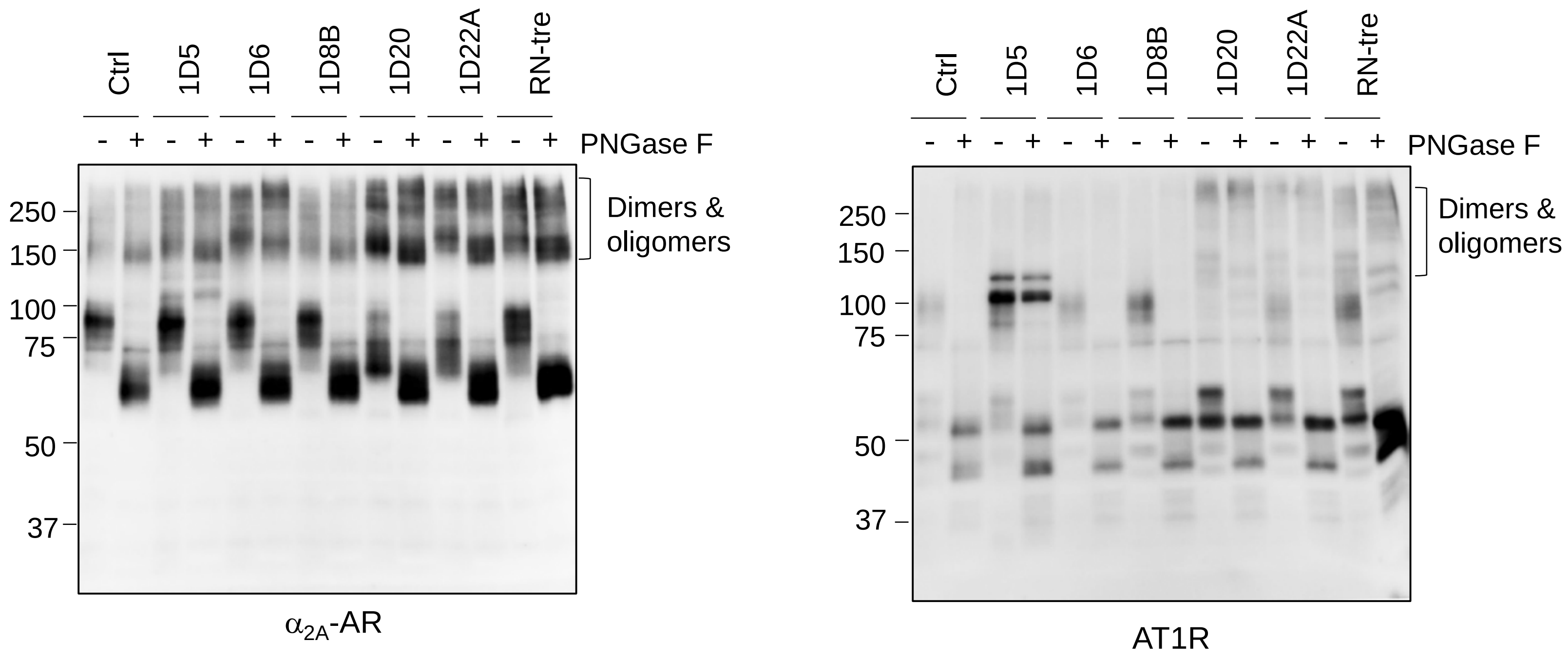

Figure S3. Effect of TBC proteins on the glycosylation of α_{2A}-AR (left panel) and AT1R (right panel). Related to Figure 4.
HEK293 cells were transfected with the receptors with or without TBC proteins. Total cell lysates were treated with PNGase F for 1 h at 37°C. Upper bands represent most likely dimers and oligomers of the receptors.

Figure S4

A

```
α2B-AR ICL3  205 KRSHCRGLGA KRGSGEGESK KPQPVAGGVP TSAKVPTLVS PLSSVGEANG HPKPPREKEE
                 GETPEDPEAR ALPPTWSALP RSGQGQKKGT SGATAEEGDEEDEEEVEECE PQTLPASPAS
                 VCNPPLQQPQ TSRVLATLRG QVLLGKNVGV ASGQWWRRRT QLSRE 369
α2A-AR ICL3  239 KRRTRVPPSR RGPDAVAAPP GGTERRPNGL GPERSAGPGG AEAEPLPTQL NGAPGEPAPA
                 GPRDTDALDL EESSSSDHAE RPPGPRRPER GPRGKGKARA SQVKPGDSLP RRGPGATGIG
                 TPAAGPGEER VGAAKASRWR GRQNRE 384

AT1R CT      303 GFLGKKFKKY FLQLLKYIPP KAKSHSSLST KMSTLSYRPS DNMSSSAKKP ASCFEVE 359
```

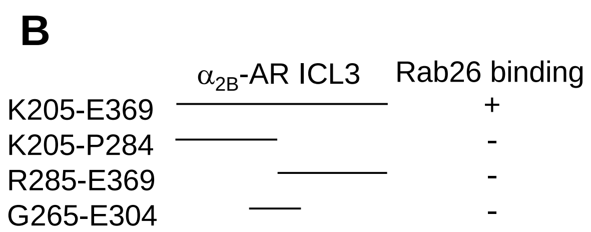

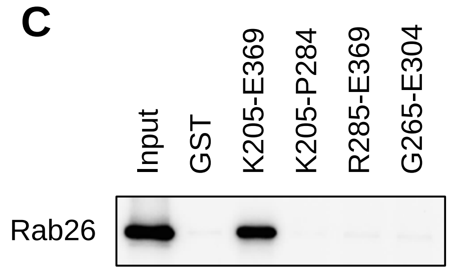

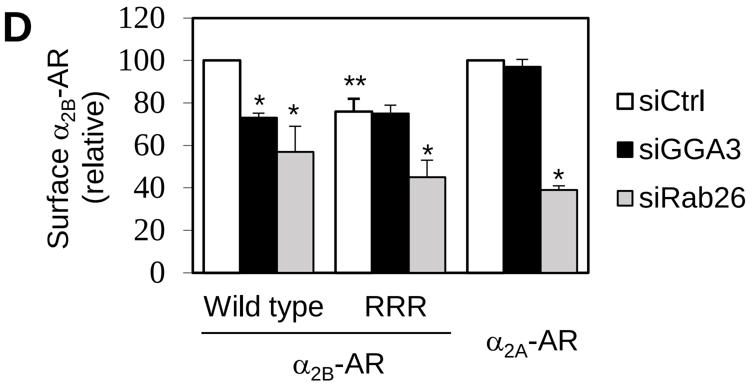

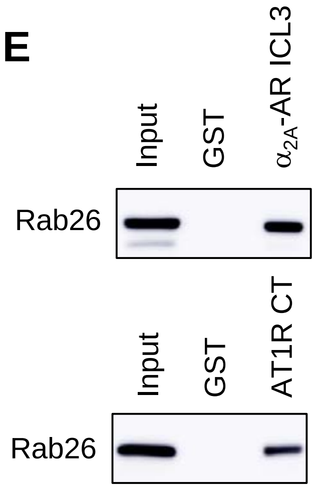

F

GPCRs		Rab26	GGA3
α_{2B}-AR	Interaction	Yes	Yes
	Interaction domain	ICL3	ICL3
	Interaction motif	No	RRR
	Cell surface transport	Yes	Yes
	Cell surface transport of RRR mutant	Yes	No
α_{2A}-AR	Interaction	Yes	No
	Interaction domain	ICL3	No
	Interaction motif	Unknown	No
	Cell surface transport	Yes	No
AT1R	Interaction	Yes	Unknown
	Interaction domain	C-terminus	Unknown
	Cell surface transport	Yes	Unknown

Figure S4. Regulation of α_{2B}-AR cell surface transport by Rab26 and GGA3 and Rab26 interaction with AT1R and α_{2A}-AR. Related to Figure 5.

(A) Sequences of the third intracellular loop (ICL3) of α_{2A}-AR and α_{2B}-AR and the C-terminus (CT) of AT1R. The GGA3-binding RRR motif is red colored.

(B) Summary of progressive deletion to identify the possible Rab26-binding domain in the ICL3 of α_{2B}-AR as shown in C.

(C) A representative blot showing the interaction of Rab26 with different ICL3 fragments. Similar results were obtained in at the least three experiments.

(D) Effect of siRNA-mediated depletion of GGA3 and Rab26 on the cell surface transport of α_{2B}-AR, the α_{2B}-AR RRR mutant in which the RRR motif was mutated to AAA, and α_{2A}-AR. The cell surface expression of the receptors was measured by intact cell ligand binding. The data shown are percentages of wild type receptor expression at the cell surface and presented as mean ± SE (n=3). *$p < 0.05$ versus respective siCtrl and **$p < 0.05$ versus wild type.

(E) The AT1R CT (upper panel) and the α_{2A}-AR ICL3 (lower panel) were generated as GST fusion proteins and their interaction with Rab26 was measured by GST fusion protein pulldown assays. Similar results were obtained in at least three separate experiments.

(F) Summary of differential regulation of GPCR transport by Rab26 and GGA3.

Figure S5

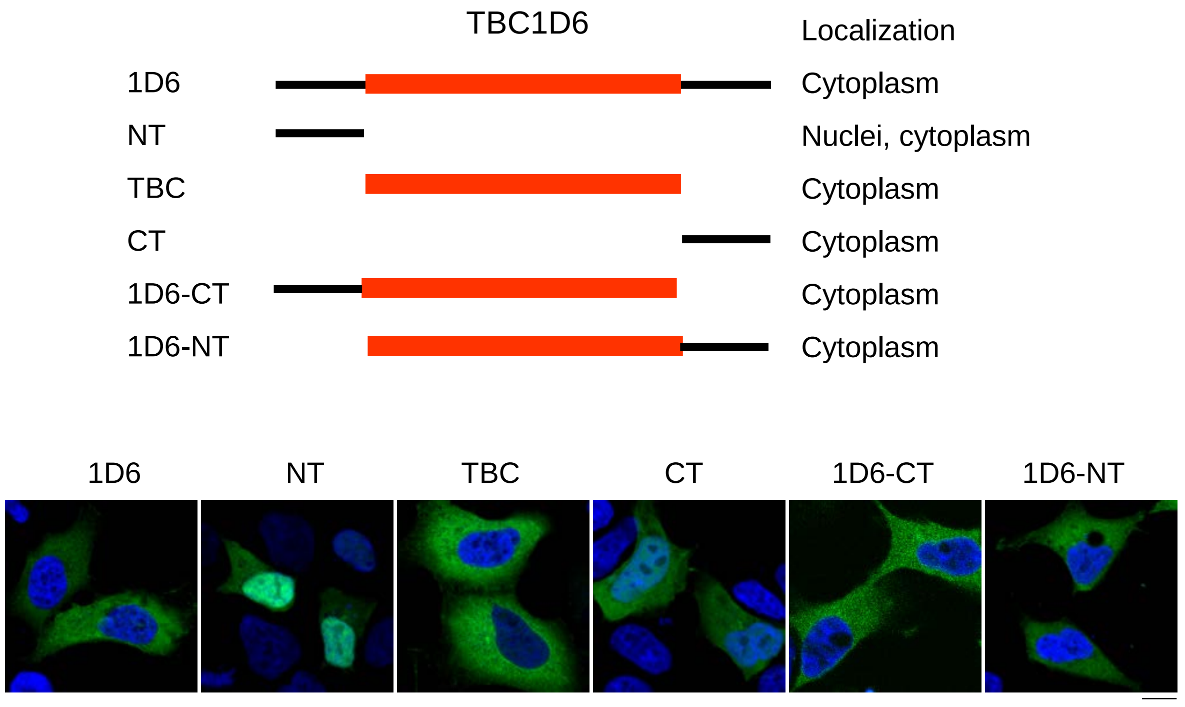

Figure S5. Expression and subcellular localization of TBC1D6 and its domains. Related to Figure 6.
TBC1D6 and its domains were tagged with GFP at their N-termini and transiently expressed in HEK293 cells. Their expression and subcellular distribution were visualized by confocal microscopy. Scale bar, 10 µm.

Figure S6

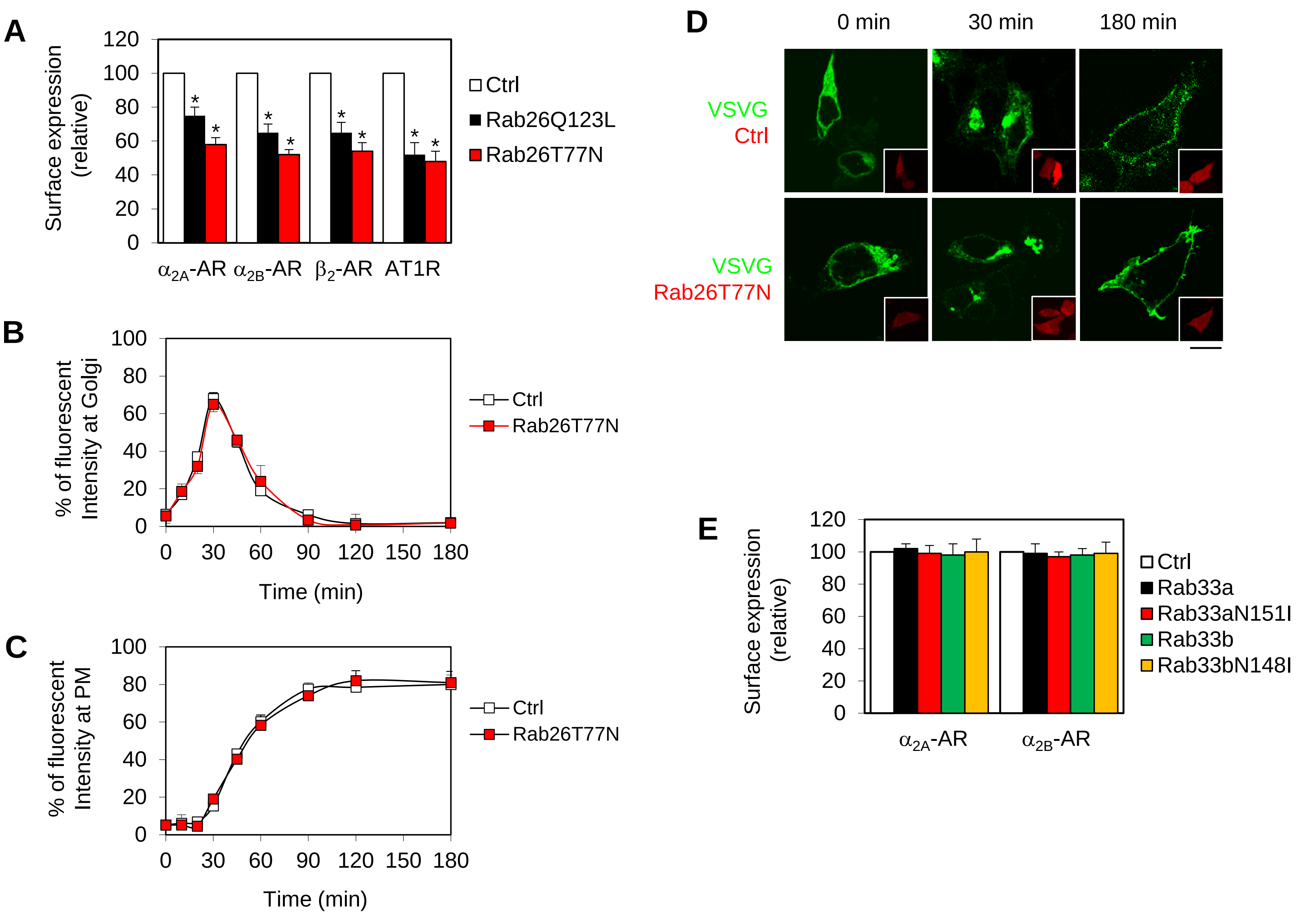

Figure S6. Effect of Rab26 and Rab33 mutants on the cell surface transport of GPCRs and VSVG. Related to Figure 7.

(A) Effect of Rab26 mutants on the cell surface expression of a group of GPCRs. HEK293 cells were transfected with individual receptors together with Rab26 mutants. The cell surface expression of the receptors was measured by intact cell ligand binding or flow cytometry.

(B) Effect of Rab26T77N on the expression of VSVG at the Golgi. HEK293 cells were transfected with VSVGtsO45-GFP together with dsRed-C1 (Ctrl) or dsRed-Rab26T77N. The cells were cultured at 40˚C for 24 h (0 min) and then shifted to 32˚C for up to 180 min. The expression of VSVG at the Golgi was measured by fluorescent intensity.

(C) Effect of Rab26T77N on the expression of VSVG at the plasma membrane (PM) as described in B.

(D) Representative images showing VSVG expression of at the Golgi after 30 min and at the plasma membrane after 180 min.

(E) Effect of Rab33 on the cell surface transport of GPCRs. HEK293 cells were transfected with α_{2A}-AR or α_{2A}-AR together with Rab33a, Rab33b or their mutants and the cell surface expression of the receptors were measured by intact cell ligand binding.

The data are presented as mean ± SE (n=3-4). *$p < 0.05$ versus Ctrl. Scale bar, 10 μm.

Table S1. Primers used in the manuscript. Related to Figures 1, 2 and 6.

TBC mutants	Sense (S) or antisence (A)	Sequences
TBC1D5 R-A	S	GAACAAGATGTCAAAGCAACGTTTCCTGAAATGC
	A	GCATTTCAGGAAACGTTGCTTTGACATCTTGTTC
TBC1D5 Q-A	S	CGAGCAGTTGCTTTATAAAGCGGGCATGCACGAACTGTTAG
	A	CTAACAGTTCGTGCATGCCCGCTTTATAAAGCAACTGCTCG
TBC1D6 R-A	S	ATCAGGACAGACCTGAACGCAACCTTCCCCGACAACGTG
	A	CACGTTGTCGGGGAAGGTTGCGTTCAGGTCTGTCCTGAT
TBC1D6 Q-A	S	CCAGGGAGTGGGCTACTGCGCGGGAATGAATTTTATAGCAG
	A	CTGCTATAAAATTCATTCCCGCGCAGTAGCCCACTCCCTGG
TBC1D8B R-A	S	GAAATTGAACGTGATTTACGTGCATCTCTGCCTGAGCACCCAGCC
	A	GGCTGGGTGCTCAGGCAGAGATGCACGTAAATCACGTTCAATTTC
TBC1D6B Q-A	S	CCAAAATTGGATACTGCGCGGCAATGAATATTTTGAC
	A	GTCAAAATATTCATTGCCGCGCAGTATCCAATTTTGG
TBC1D20 R-A	S	CAAGTGTTGCTGGACGTCCGGGCGTCATTGCGGCGGTTCCCTCCTG
	A	CAGGAGGGAACCGCCGCAATGACGCCCGGACGTCCAGCAACACTTG
TBC1D20 Q-A	S	CCTCAGCTGCACTACTACGCGGGCTACCATGACATTGTG
	A	CACAATGTCATGGTAGCCCGCGTAGTAGTGCAGCTGAGG
TBC1D22A R-A	S	CAGATCCACATAGACATCCCTGCCATGAGCCCTGAAGCGTTGATC
	A	GATCAACGCTTCAGGGCTCATGGCAGGGATGTCTATGTGGATCTG
TBC1D22A Q-A	S	CCAGCCAGTGGATACGTTGCGGGTATAAATGATCTCGTC
	A	GACGAGATCATTTATACCCGCAACGTATCCACTGGCTGG
RN-tre R-A	S	CAAATAGACCTGGATGTCAACGCCACATTTCGGGACCACATTATG
	A	CATAATGTGGTCCCGAAATGTGGCGTTGACATCCAGGTCTATTTG
RN-tre Q-A	S	CACGGAAGTCGGGTATTGTGCGGGGATGGACCAGATCACAG
	A	CTGTGATCTGGCTCATCCCCGCACAATACCCGACTTCCGTG
TBC proteins cloned into the dsRed monomer-C1 vector		
TBC1D5	S	ATGCCTCGAGCTTATCATTCCTTATCTGAAACTAGACATCC
	A	ATGCGGATCCTCAGATGTCCAGGGGACTCACAATGGT
TBC1D6	S	ATGCGAATTCTCAGCCCGCCGAGCGCTCGCGG
	A	ATGCGGATCCTCATGCCCACTGGTGCCCGTC
TBC1D8B	S	ATGCGAATTCTGCTACTAATCCTGACTATTATAC
	A	ATGCGGATCCTCAGCAGCCTCTGAACTGCTGGC
TBC1D20	S	ATGCCTCGAGCTGCCCTCCGGAGTGCGCAGGGC
	A	ATGCGAATTCTCAGGGAAACAGCTGCAGCTGAAA
TBC1D22A	S	GATCGAATTCTGCCAGCGACGGGGCCAGGAAG
	A	GATCGGATCCTCATTTCTTGTAGTGATTGGGGGC
RN-tre	S	ATGCGAATTCTAATTCAGACCAGGATGTAGCACTC
	A	ATGCGGATCCTCACAGCAACACTGACTCTTGGATGG
TBC1D6 domains cloned into the pEGFP-C1 vector		
N-terminus	S	ATGCGAATTCTCAGCCCGCCGAGCGCTCGCGG
	A	ATGCGGATCCTCAATAGCGCTTCACTGTCCGGCTC
TBC	S	ATGCGAATTCTGTCCGGAAAGGGGTCCCGC
	A	ATGCGGATCCTCACAAAATCAACTCCTGGTGCTGCT
C-terminus	S	ATGCGAATTCTGAAGCCACCAGCGTTCCAGAC
	A	ATGCGGATCCTCATGCCCACTGGTGCCCGTC
1D6-N-terminus	S	ATGCGAATTCTGTCCGGAAAGGGGTCCCGC
	A	ATGCGGATCCTCATGCCCACTGGTGCCCGTC
1D6-C-terminus	S	ATGCGAATTCTCAGCCCGCCGAGCGCTCGCGG
	A	ATGCGGATCCTCACAAAATCAACTCCTGGTGCTGCT

TBC and Rab proteins cloned into the pEGX-4T-1 vector		
TBC1D6	S	GATCGGATCCCAGCCCGCCGAGCGCTCGCGG
	A	GATCCTCGAGTGCCCACTGGTGCCCGTCCTG
1D6-C-terminus		GATCGGATCCCAGCCCGCCGAGCGCTCGCGG
		ATGCCTCGAGCAAAATCAACTCCTGGTGCTGCT
Rab26	S	ATGCGGATCCTCCAGGAAGAAGACCCCCAAGA
	A	ATGCGAATTCTCAAGGGCGGCAGCAG
Rab33 mutants		
Rab33aN151I	S	AAAGTGCTTGTGGGCATCAAGTGTGACTTGAGG
	A	CCTCAAGTCACACTTGATGCCCACAAGCACTTT
Rab33bN148I	S	CCTCGAATTCTGGTGGGAATTAAATGTGACTTGAGAAGC
	A	GCTTCTCAAGTCACATTTAATTCCCACCAGAATTCGAGG
